# The multifunctional protein E4F1 links P53 to lipid metabolism in adipocytes

**DOI:** 10.1038/s41467-021-27307-3

**Published:** 2021-12-02

**Authors:** Matthieu Lacroix, Laetitia K. Linares, Natalia Rueda-Rincon, Katarzyna Bloch, Michela Di Michele, Carlo De Blasio, Caroline Fau, Laurie Gayte, Emilie Blanchet, Aline Mairal, Rita Derua, Fernando Cardona, Diane Beuzelin, Jean-Sebastien Annicotte, Nelly Pirot, Adeline Torro, Francisco J. Tinahones, Florence Bernex, Justine Bertrand-Michel, Dominique Langin, Lluis Fajas, Johannes V. Swinnen, Laurent Le Cam

**Affiliations:** 1 IRCM, Institut de Recherche en Cancérologie de Montpellier, INSERM U1194, Univ Montpellier, Institut régional du Cancer de Montpellier, Montpellier, France; 2 Equipe labélisée Ligue Contre le Cancer, Paris, France; 3 KU Leuven–University of Leuven, Department of Oncology, Laboratory of Lipid Metabolism and Cancer, Leuven, Belgium; 4 I2MC, Institute of Metabolic and Cardiovascular Diseases, Université de Toulouse, INSERM, Université Toulouse III – Paul Sabatier (UPS), Toulouse, France; 5 KU Leuven–University of Leuven, Department of Cellular and Molecular Medicine, Leuven, Belgium; 6 Department of Surgical Specialties, Biochemistry and Immunology School of Medicine, University of Malaga, Malaga, Spain; 7 Univ. Lille, Inserm, CHU Lille, Institut Pasteur de Lille, CNRS, U1283 - UMR 8199 - EGID, Lille, France; 8 BioCampus, RHEM, Université de Montpellier, CNRS, INSERM, Montpellier, France; 9 CIBER of Physiopathology, Obesity and Nutrition (CIBEROBN), Málaga, Spain; Unidad de Gestion Clinica de Endocrinologia y Nutrición, Instituto de Investigación Biomédica de Málaga (IBIMA), Hospital Clinico Virgen de la Victoria, Málaga, Spain; 10 Toulouse University Hospitals, Department of Clinical Biochemistry, Toulouse, France; 11 Center for Integrative Genomics, University of Lausanne, Lausanne, Switzerland

**Keywords:** Transcription, Fat metabolism, Obesity

## Abstract

Growing evidence supports the importance of the p53 tumor suppressor in metabolism but the mechanisms underlying p53-mediated control of metabolism remain poorly understood. Here, we identify the multifunctional E4F1 protein as a key regulator of p53 metabolic functions in adipocytes. While E4F1 expression is upregulated during obesity, *E4f1* inactivation in mouse adipose tissue results in a lean phenotype associated with insulin resistance and protection against induced obesity. Adipocytes lacking E4F1 activate a p53-dependent transcriptional program involved in lipid metabolism. The direct interaction between E4F1 and p53 and their co-recruitment to the *Steaoryl-CoA Desaturase-1* locus play an important role to regulate monounsaturated fatty acids synthesis in adipocytes. Consistent with the role of this E4F1-p53-*Steaoryl-CoA Desaturase-1* axis in adipocytes, *p53* inactivation or diet complementation with oleate partly restore adiposity and improve insulin sensitivity in E4F1-deficient mice. Altogether, our findings identify a crosstalk between E4F1 and p53 in the control of lipid metabolism in adipocytes that is relevant to obesity and insulin resistance.

## Abstract (web summary)

The p53 tumor suppressor is also a regulator of metabolism, but the mechanisms controlling p53-associated metabolic activities remain poorly understood. Here the authors report that the deletion of the multifunctional protein E4F1 is protective against diet-induced obesity in mice, and E4F1 regulates adipocyte lipid metabolism through p53.

## Introduction

The *Tp53* tumor suppressor encodes a ubiquitously expressed transcription factor activated in response to numerous extrinsic and intrinsic challenges to the cell, including DNA damage, oxidative stress, nutrient deprivation, oncogene activation, or hypoxia. p53 promotes a variety of cellular responses that rely on the type of stress, its severity, and persistence, as well as the cell type in which it occurs. p53 controls an efficient safeguard mechanism that prevents the accumulation of abnormal cells and their transformation by regulating DNA repair, cell cycle progression, cell death, or senescence^1,2^. The diversity of cellular processes regulated by p53 was recently extended to the control of metabolism. Deregulation of p53-associated metabolic activities has been linked to tumor development, as well as to other pathophysiological conditions including aging, obesity, type 2 diabetes, and liver disease^3–7^.

p53 controls many metabolic pathways by activating or repressing genes implicated in glycolysis, glutaminolysis, oxidative phosphorylation, amino-acid metabolism, and the pentose phosphate pathway (PPP). Several reports have highlighted the importance of p53 in lipid metabolism through the direct transcriptional regulation of genes implicated in fatty acid degradation and lipid synthesis^8^, including *Lipin1 (Lpin)*^9^*, carnitine O-octanoyltransferase* (*CROT*)^10^, *carnitine palmitoyltransferase 1C (Cpt1c)*^11^*, Sterol Regulatory Element Binding Protein 1c (Srebp1)*^12^, *Acad11*^13^, and *Npc1L1*^14^. Although the importance of p53 in fatty acid metabolism is gaining momentum, the upstream mechanisms controlling p53 activities in lipid homeostasis remain poorly understood. Here, we identify the multifunctional protein E4F1 as a key regulator of p53-mediated control of lipid metabolism.

E4F1 was originally identified as a cellular target of the viral oncoprotein E1A and was characterized as a transcriptional regulator of the viral E4 promoter during adenoviral infection^15,16^. Since, mouse genetic studies indicated that *E4f1* is essential during early embryogenesis^17^, as well as in various adult tissues in which it controls stem cell maintenance^18,19^. Besides its intrinsic transcriptional activities, E4F1 displays an atypical E3 ligase function that targets the p53 tumor suppressor. Strikingly, E4F1-mediated ubiquitylation of p53 modulates its transcriptional activities but not its degradation^20^. The multifunctional protein E4F1 controls the balance between proliferation and cell survival through various mechanisms involving the p53 and pRB tumor suppressors^20–22^, the post-transcriptional stabilization of the cdk inhibitor p21^[WAF123^, the transcriptional repression of the *cyclin A2* promoter^24,25^, and the regulation of the DNA-damage response^26–28^. Interestingly, E4F1 was also shown to control metabolism through the transcriptional regulation of genes encoding essential components or regulators of the pyruvate dehydrogenase complex, a mitochondrial complex that fuels the tri-carboxylic acid (TCA) cycle by converting pyruvate into AcCoA^29,30^. Here we show that E4F1 is a key regulator of p53-associated metabolic functions in adipocytes that plays an important role in obesity and insulin sensitivity.

## Results

### E4F1 has a critical role in adipogenesis

**Fig. 1:**
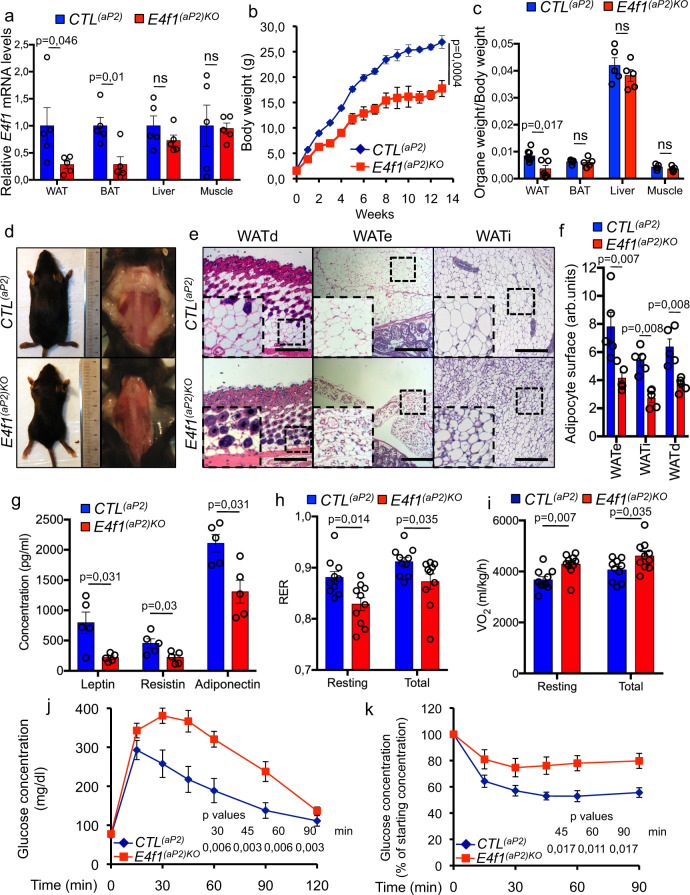
E4F1 plays a critical role in adipogenesis. **a** RT-qPCR analysis of *E4f1* mRNA levels in visceral white (WAT) and brown (BAT) adipose tissue, liver, and skeletal muscle of *E4f1*^(*aP2*)*KO*^ mice and control (*CTL*^*(aP2)*^) littermates (*n* = 5 animals/group). **b** Body weight of *E4f1*^(*aP2*)*KO*^ mice and *CTL*^*(aP2)*^ littermates under chow diet (*n* = 7 males/group). **c** Weight of visceral WAT, BAT, liver, and muscle (gastronecmius) of *E4f1*^(*aP2*)*KO*^ animals and *CTL*^*(aP2)*^ littermates (*n* = 5 males/group). Data were normalized to total body weight (BW) to account for the disparity in body size between the two groups. **d** Representative microphotographs of *E4f1*^(*aP2*)*KO*^ and *CTL*^*(aP2)*^ littermates (left panels) and of their interscapular fat depots (right panels) (analyses were performed on *n* = 10 animals/group). **e** Representative microphotographs of hematoxylin and eosin (H&E) -stained sections of subcutaneous (WATd), epidydymal (WATe), and inguinal (WATi) fat depots prepared from *E4f1*(*aP2*)^*KO*^ mice and *CTL*^*(aP2)*^ littermates (*n* = 5 males/group). Scale bars, 500 μm. **f** Adipocyte surface area (arb. units, arbitrary units) was determined from H&E-stained sections of WATd, WATe, and WATi fat depots of *E4f1*^(*aP2*)*KO*^ mice and *CTL*^*(aP2)*^ littermates (*n* = 5 males/group). **g** Circulating levels of leptin, resistin, and adiponectin in the plasma of 8–12-week-old *E4f1*^(*aP2*)*KO*^ mice and *CTL*^*(aP2)*^ littermates (*n* = 5 males/group). **h** Respiratory Exchange Ratio (RER) of *E4f1*^(*aP2*)*KO*^ mice and *CTL*^*(aP2)*^ littermates determined during the resting period (12 h light) or during 24 h (total) (n = 10 males/group). **i** Whole-body O_2_ consumption (VO_2_) in *E4f1*^(*aP2*)*KO*^ mice and *CTL*^*(aP2)*^ littermates during the same period as in **h** (*n* = 10 males/group). **j**, **k** Glucose tolerance test (IPGTT) (**j**) and insulin tolerance test (ITT) (**k**) performed on 8–12-week-old *E4f1*^(*aP2*)*KO*^ mice and *CTL*^*(aP2)*^ littermates (*n* = 6 males/group). Data are presented as mean ± standard error mean (SEM) from the indicated number of animals. Statistical analyses were performed using two-sided non-parametric Mann–Whitney *U* tests and the BiostaTGV software (ns, not significant). Source data are provided as a Source Data file.

To evaluate the physiological roles of *E4f1* in adult tissues, we crossed *E4f1* constitutive and conditional knock-out (KO) mice (hereafter referred to as *E4f1*^−^ and *E4f1*^flox^, respectively) with mice expressing the tamoxifen (tam)-inducible CreER^T2^ fusion protein under the control of the ubiquitously active *RNA polymerase II large subunit* promoter (*RERT*)^17,18^. Administration of tam to 8–12-week-old *E4f1*^-/flox^; *RERT* mice led to the Cre-mediated inactivation of the remaining *E4F1*^flox^ allele (hereafter referred to as *E4f1*^*(RERT)KO*^ mice) with high efficiency in most tissues except the central nervous system, including in white and brown adipose tissues (WAT and BAT, respectively). As soon as 15 days upon tam administration, we observed a 30% decrease of *E4f1*^*(RERT)KO*^ animals body weight and a severe loss of adiposity relative to control littermates (*CTL*^*(RERT)*^*)* (Fig. S1a–d). To confirm these adipose tissue defects, we crossed *E4f1*^-/flox^ mice with transgenic animals that expressed the Cre recombinase under the control of the *Fatty acid-binding protein 4* (*Fabp4/aP2*) promoter (*E4f1*^(aP2)KO^ mice)^31^. Efficient Cre-mediated inactivation of *E4f1* in all adipose tissue depots, including BAT, was confirmed by qPCR and RT-qPCR on genomic DNA and total RNAs, respectively (Fig. 1a and Supplementary Fig. S2a). Depletion of E4F1 was confirmed on isolated adipocytes prepared from epidydymal WAT (WATe) of *E4f1*^*(aP2)KO*^ mice (Supplementary Fig. S2b). Mice lacking E4F1 in adipocytes gained less weight than controls (*CTL*^(*aP2*)^) as they aged, although we observed no difference in food intake and locomotor activity (Fig. 1b and Supplementary Fig. S2c,d). Both *E4f1*^*(aP2)KO*^ males and females were lean under chow diet and showed little or no interscapular, inguinal, and subcutaneous fat depot accumulation over time (Fig. 1c, d and Supplementary Fig. S2e). Lean mass of *E4f1*^*(aP2)KO*^ mice remained macroscopically unaffected, as illustrated by the normal weight of muscles and liver (Fig. 1c). Histological analyses performed on hematoxylin and eosin (H/E)-stained tissue sections prepared from various adipose tissue depots of *E4f1*^*(RERT)KO*^ and *E4f1*^*(aP2)KO*^ mice indicated that WAT was not completely absent but was composed of smaller adipocytes with a lipid droplet of reduced surface area (Fig. 1e, f). In situ analysis of adipose tissue sections failed to detect evidence of cleaved caspase-3 staining, suggesting that the reduction of white fat mass in *E4f1*^*(RERT)KO*^ and *E4f1*^*(aP2)KO*^ animals were not the consequence of massive apoptosis (Supplementary Figs. S1e and S2f). Importantly, histological analyses and immunodetection of the Mac2 or F4/80 macrophage markers showed no infiltration by inflammatory cells in the WAT of *E4f1*^*(aP2)KO*^ mice (Fig. S3a). Moreover, the circulating levels of the pro-inflammatory cytokines Il6 and MCP1 were lower in *E4f1*^*(aP2)KO*^ mice relative to control littermates (Supplementary Fig. S3b), further supporting the notion that the lean phenotype observed upon E4F1 deficiency was not associated with adipose tissue inflammation.

To determine whether this severe phenotype resulted from defects in lipid storage, increased lipolysis, or enhanced lipid oxidation in WAT, we first evaluated the levels of circulating lipids and adipokines in *E4f1*^*(aP2)KO*^ mice and measured other metabolic parameters including their respiratory exchange ratio (RER). Free fatty acids (FFA), total cholesterol and high-density lipoprotein (HDL) levels were lower in the plasma of *E4f1*^*(aP2)KO*^ animals (Table 1). Moreover, the levels of circulating adipokines, including leptin, resistin, and adiponectin, decreased upon *E4f1* inactivation in adipocytes (Fig. 1g). In addition, animals lacking E4F1 in their adipose tissue did not display liver steatosis and even exhibited decreased lipid accumulation when compared to control littermates, as shown by reduced Oil Red O (ORO) staining of liver cryosections (Supplementary Fig. S3c). These data suggested that the lean phenotype of *E4f1*^*(aP2)KO*^ mice was neither the consequence of defects in lipid storage nor resulted from increased lipolysis in WAT, but rather reflected increased energy expenditure. Consistent with this notion, we found that the RER (vCO_2_/vO_2_, resting and total), measured either at room temperature (RT) or at thermoneutral temperature (29 °C), significantly decreased in *E4f1*^*(RERT)KO*^ and *E4f1*^*(aP2)KO*^ animals and this correlated with increased oxygen consumption (Fig. 1h, i and Supplementary Figs. S1f and S3d). Moreover, the levels of ketone bodies were higher in the plasma of *E4f1*^*(aP2)KO*^ animals than in control mice, indicating that E4F1 deficiency triggered increased lipid oxidation (Table 1).

**Table 1:** Metabolic characterization of mice lacking E4F1 in their adipose tissue.

Concentration (mM)	*CTL*^*(aP2)*^	*E4f1*^*(aP2)KO*^
Ketone bodies	0.14 ± 0.02	0.24 ± 0.02^*p* = 0.026^
Triglycerides	1.31 ± 0.12	1.06 ± 0.19 ^ns^
FFA	3.22 ± 0.57	1.61 ± 0.38^*p* = 0.011^
Cholesterol	4.68 ± 0.25	1.59 ± 0.51^*p* = 0.0079^
HDL	1.44 ± 0.2	0.39 ± 0.12^*p* = 0.016^
LDL	0.48 ± 0.07	0.43 ± 0.04 ^ns^

Circulating levels of ketone bodies, triglycerides, free fatty acids (FFA), total cholesterol, HDL, and LDL in the plasma of 8–12-week-old *E4f1*^(*aP2*)*KO*^ mice and *CTL*^*(aP2)*^ littermates (*n* = 5 males/group). Data were presented as mean ± standard error mean (SEM) from the indicated number of independent samples. Statistical analyses were performed using two-sided non-parametric Mann–Whitney *U* tests and the BiostaTGV software (ns, not significant). Source data are provided as a Source Data file.

Although lower body mass index is often associated with improved glucose tolerance and insulin sensitivity, mouse models in which adiposity is severely reduced such as lipodystrophic mice, can develop insulin resistance^32^. Insulin and glucose tolerance tests showed that *E4f1*^*(aP2)KO*^ mice were insulin resistant and glucose intolerant (Fig. 1j, k and Supplementary Fig. S3e). Taken together, these data indicate that *E4f1* inactivation in adipose tissue results in a lean phenotype associated with alterations of glucose homeostasis.

### *E4f1* inactivation protects against induced obesity and E4F1 expression is upregulated during obesity

**Fig. 2:**
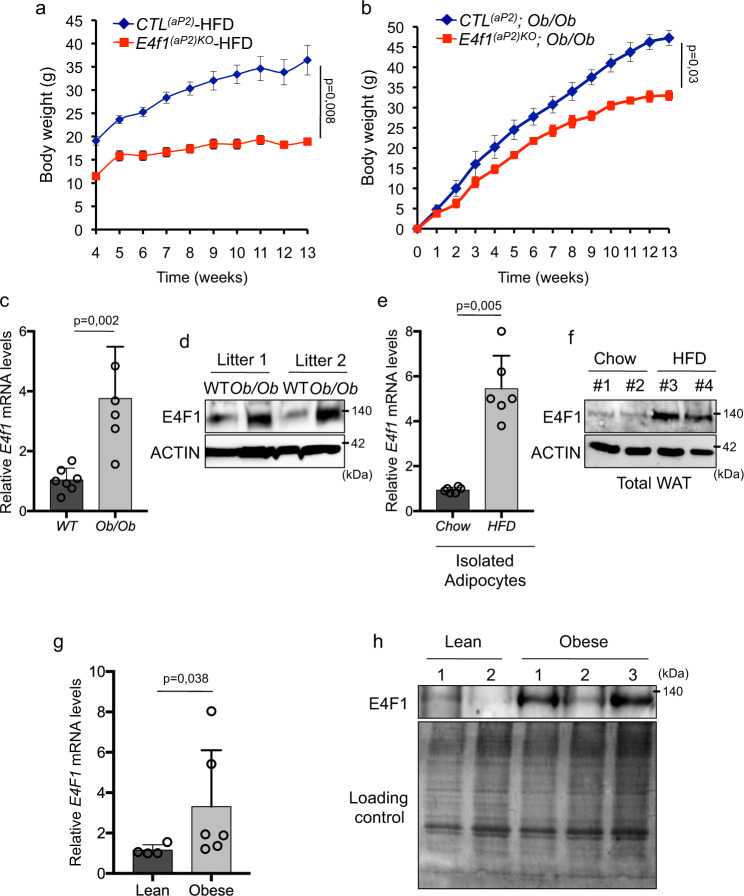
*E4f1* function in obesity. **a** Body weight of *E4f1*^(*aP2*)*KO*^ mice and *CTL*^*(aP2)*^ under high-fat diet (HFD) (*n* = 5 males/group). **b** Body weight of *E4f1*^(*aP2)KO*^*; Ob/Ob* mice and *CTL*^*(aP2)*^*; Ob/Ob* littermates (*n* = 4 males/group). **c** RT-qPCR analysis of *E4f1* mRNA levels in epidydymal white adipose tissue (WATe) of 12-week-old *Ob/Ob* mice and control littermates (*n* = 6 males/group). **d** Immunoblot analysis of E4F1 and ACTIN (loading control) protein levels in WATe of 12-week-old wild-type (WT) or *Ob/Ob* mice. **e** RT-qPCR analysis of *E4f1* mRNA levels in isolated adipocytes prepared from WATe of mice fed with normal chow or an HFD during 4 weeks (*n* = 6 males/group). **f** Immunoblot analysis of E4F1 protein levels in WATe of mice fed with normal chow or an HFD during 4 weeks. **g** RT-qPCR analysis of *E4F1* mRNA levels in visceral WAT from lean individuals (*n* = 4 individuals) or patients with obesity (*n* = 6 individuals). **h** Immunoblot analysis of E4F1 protein levels in visceral WAT from lean individuals or patients with obesity. Red Ponceau staining was performed to confirm equal loading of samples. Molecular weights  are indicated in kDa. Data  are presented as mean ±  standard error mean (SEM) from the indicated number of independent samples. Statistical analyses were performed using two-sided non-parametric Mann–Whitney *U* tests and the BiostaTGV software (ns, not significant). Source data are provided as a Source Data file.

The reduced adiposity of *E4f1*^*(aP2)KO*^ mice prompted us to determine their resistance to induced obesity. When challenged on a high-fat diet (HFD), *E4f1*^*(aP2)KO*^ mice displayed resistance to diet-induced obesity (Fig. 2a). Moreover, *E4f1*^*(aP2)KO*^ mice in the *leptin*-deficient KO (*Ob*) background also accumulated significantly less fat than their control *Ob/Ob* littermates (Fig. 2b). Next, we examined the expression of *E4f1* during obesity. Both *E4f1* protein and mRNA levels were significantly higher in epididymal fat of *Ob/Ob* mice, as well as in mice under HFD when compared to their respective control mice (Fig. 2c–f). Consistent with these findings, untreated patients with obesity displaying a body mass index (BMI) superior to 47 displayed higher *E4F1* mRNA and protein levels in visceral fat when compared to lean individuals (Fig. 2g, h and Table 2). Altogether, these results support an important role for E4F1 in obesity.

**Table 2:** E4F1 expression in patients with obesity.

Individuals	Sex	Age (y)	Weight (kg)	Size (cm)	BMI
Lean individual #1	F	45	59.0	172	19.9
Lean individual #2	F	53	59.0	168	20.9
Lean individual #3	F	52	63.0	162	24.0
Lean individual #4	M	73	65.0	166	23.6
Patient with obesity #1	F	42	137.0	154	57.8
Patient with obesity #2	M	42	150.0	171	51.3
Patient with obesity #3	F	43	135.0	155	56.2
Patient with obesity #4	M	52	189.2	170	65.5
Patient with obesity #5	M	33	164.0	186	47.4
Patient with obesity #6	M	38	155.3	180	47.9

Clinical data (sex, age (years), weight (kg), size (cm), and body mass index (BMI)) of patients with obesity and lean individuals were analyzed in this study.

### E4F1 dysfunction results in adipocyte differentiation and lipid accumulation defects

**Fig. 3:**
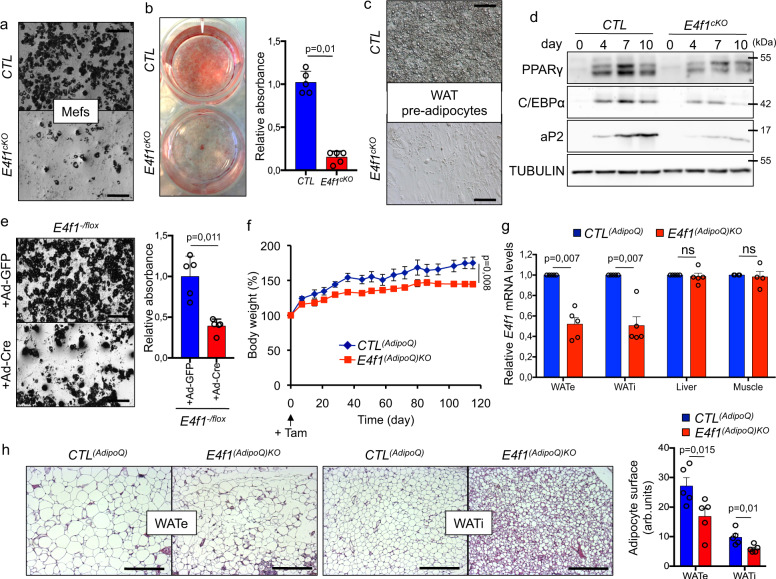
E4F1 dysfunction results in adipocyte differentiation and lipid metabolism defects. **a** Representative microphotographs of *E4f1*^*cKO*^ and CTL Mefs, 10 days after induction of adipocyte differentiation. Scale bars, 100 μm. **b** Representative microphotographs at low-magnification of the same cells than in **a** upon Oil Red O (ORO) staining. Histobars represent the quantification of triglycerides (TG) accumulation upon ORO staining (*n* = 7 independent populations of Mefs/group). **c** Representative microphotographs of *E4f1*^*cKO*^ and CTL adipocytes, 10 days after induction of adipocyte differentiation using primary pre-adipocytes isolated from WAT of adult *E4f1*^-/flox^ animals (*n* = 5 experiments performed with independent populations of cells). Scale bars, 200 μm. **d** Representative immunoblot analysis of *PPARγ*, *C/EBPα*, *FABP4/aP2*, and Tubulin (loading control) protein levels in *E4f1*^cKO^ or CTL Mefs at the indicated time points after induction of adipocyte differentiation (*n* = 5 experiments performed with independent populations of cells). For experiments described in **a** to **d**, cells were transduced with a Cre-encoding retrovirus 5 days prior to the induction of adipocyte differentiation. **e** Representative microphotographs at low-magnification of *E4f1*^*-/flox*^ differentiated Mefs transduced with Cre- or GFP- adenoviruses 7 days after induction of adipocyte differentiation. Histobars represent the quantification of TG accumulation upon ORO staining, 10 days after *E4f1* inactivation (*n* = 5 independent populations of Mefs/group). Scale bars, 100 μm. **f** Body weight of *E4f1*^(*AdipoQ*)*KO*^ mice and *CTL*^*(AdipoQ)*^ control littermates. Data are represented as percentages of the initial body weight at the time of the first tamoxifen (tam) administration (*n* = 5 males/group). **g** RT-qPCR analysis of *E4f1* mRNA levels in WATe, WATi, liver, and skeletal muscles of *E4f1*^(*AdipoQ*)*KO*^ mice and *CTL*^(*AdipoQ*)^ littermates after 16 weeks of repeated tam administration (n = 5 males/group). **h** Representative microphotographs of H&E-stained sections of WATe and WATi fat depots were prepared from the same animals as in **g**. Scale bars, 500 μm. Histobars represent adipocyte surface area (arb. units, arbitrary units, *n* = 5 males/group). Molecular weights are indicated in kDa. Data are presented as mean ± standard error mean (SEM) from the indicated number of independent samples. Statistical analyses were performed using two-sided non-parametric Mann–Whitney *U* tests and the BiostaTGV software (ns, not significant). Source data are provided as a Source Data file.

To further investigate *E4f1* function in adipocytes at the molecular and metabolic levels, we performed adipocyte differentiation assays in vitro using mouse embryonic fibroblasts (Mefs) prepared from *E4f1*^*-/flox*^ embryos, and primary pre-adipocytes isolated from WAT of *E4f1*^*-/flox*^ adult mice. We triggered *E4f1* inactivation in these cells upon transduction with a self-excising Cre-encoding retrovirus (*E4F1*^cKO^ cells) and then monitored adipogenic differentiation over a period of 10 days^33^. *E4f1*^cKO^ Mefs exhibited impaired lipid accumulation during adipocyte differentiation, as illustrated by reduced ORO staining of neutral lipids (Fig. 3a, b). Importantly, and consistent with our in situ analyses, comparable numbers of *E4f1*^cKO^ and *CTL* cells were present at day 10 (Supplementary Figure S4a), excluding the possibility that this phenotype resulted from increased cell death. Notably, accumulation of TG during in vitro differentiation was similarly impaired upon *E4f1* inactivation in primary pre-adipocytes isolated from adult WAT (Fig. 3c).

To gain further insights into the defects of E4F1-deficient adipocytes, we next measured the mRNA levels of the WAT differentiation markers *Fabp4/aP2, Peroxisome proliferator activated receptor gamma* (*Pparγ), Srebp1c* and the *CCAAT/enhancer binding protein alpha (C/ebpα*) during adipocyte differentiation (0, 4, 7, and 10 days after induction of adipocyte differentiation). At the transcriptional level, these adipogenic genes were similarly induced in *E4f1*^cKO^ and *CTL* cells, with the exception of *Fabp4,* which mRNA levels were reduced at days 4 and 7 (Supplementary Fig. S4be). However, the protein levels of FABP4/aP2, PPARg, and C/EBPα decreased in *E4f1*^cKO^ cells (Fig. 3d), indicating that *E4f1* inactivation impinged on the induction of these differentiation markers during in vitro adipocyte differentiation mainly through a post-transcriptional mechanism. Importantly, the mRNA levels of the brown fat-enriched genes *Prmd16, Elongation of Very Long chain fatty acids gene 3* (*Elovl3*), *Cell Death-Inducing DNA Fragmentation Factor-like Effector A* (*Cidea*), and the *β3 adrenergic receptor* (*AdRβ3*) were not significantly different between E4F1-proficient and deficient adipocytes, indicating that the latter did not transdifferentiate into brown adipocytes (Supplementary Fig. S4c).

To assess the metabolic consequences of E4F1 deficiency in differentiated adipocytes, we next used a Cre-encoding adenovirus to trigger *E4f1* inactivation in *E4f1*^-/flox^ Mefs 7 days after induction of adipocyte differentiation, a time point when PPARγ, C/EBPα, and the differentiated marker FABP4/aP2 were already fully induced. In these experimental conditions, *E4f1* inactivation also resulted in decreased TG accumulation 10 days after the addition of Cre-adenovirus, but failed to impact on *Pparg* and *Fabp4* mRNA levels (Fig. 3e and Supplementary Fig. S4d). To confirm the importance of E4F1 in fully differentiated adipocytes in vivo, we then crossed *E4f1*^*-/flox*^ animals expressing the tam-inducible CreER^T2^ recombinase under the control of the adipocyte-specific *Adiponectin* promoter (*AdipoQ* mice*)*^34^. Repeated tam administration to adult *E4f1*^*-/flox*^*; AdipoQCreER*^*T2*^ (*E4f1*^*(adipoQ)KO*^*)* during 12 weeks showed that these mice gained less weight over time than their control littermates (*CTL*^*(adipoQ)*^) (Fig. 3f). Histological and RT-qPCR analyses indicated that their reduced adiposity in different adipose tissue depots correlated with the efficiency of Cre-mediated recombination of the *E4f1*^flox^ allele, ranging from 40 to 60% (Fig. 3g, h). Similar to *E4f1*^*(aP2)KO*^ mice, *E4f1*^*(adipoQ)KO*^ animals exhibited insulin resistance and glucose intolerance (Supplementary Figure S4e, f). Thus, these results show that E4F1 deficiency affects both adipocyte differentiation and the ability of differentiated white adipocytes to store lipids.

### *E4f1*^cKO^ adipocytes display impaired de novo lipid synthesis and increased fatty acid oxidation

**Fig. 4:**
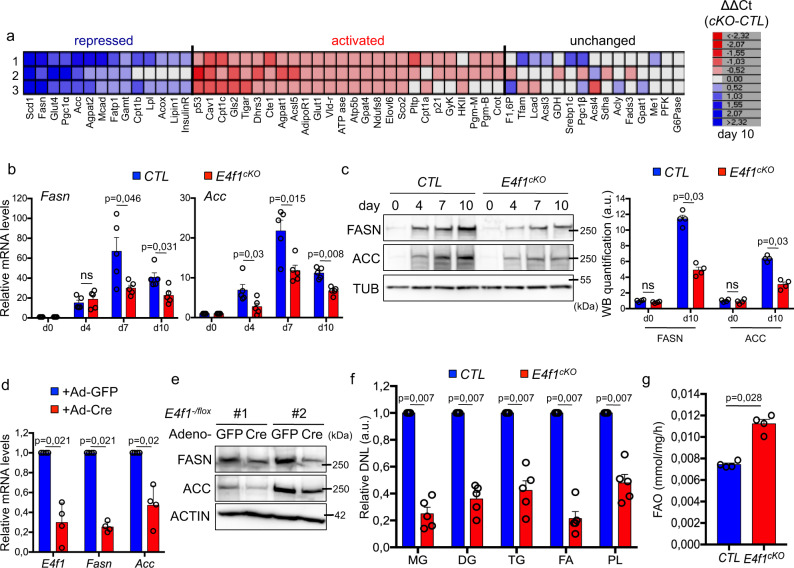
*E4f1* inactivation results in decreased fatty acid synthesis and increased fatty acid oxidation (FAO). **a** Gene expression profile of metabolic genes determined by a microfluidic RT-qPCR approach during adipocyte differentiation of *E4f1*^*cKO*^ and CTL primary pre-adipocytes. The heat maps represent the relative mRNA levels of the indicated genes, 10 days after induction of adipocyte differentiation, calculated using the ΔΔCt method between *E4f1*^*cKO*^ and CTL cells. Raw data were normalized by three independent housekeeping genes (β2m, 18 S, tubulinb5). Each line represents the results of one independent experiment performed on paired *E4f1*^*cKO*^ pre-adipocytes and CTL cells. **b** RT-qPCR analysis of *Fasn* (left panel) and *Acc* (right panel) mRNA levels in *E4f1*^cKO^ or CTL Mefs at the indicated time points after induction of adipocyte differentiation (*n* = 5 independent populations of cells/group). **c** Quantitative immunoblot analysis of FASN, ACC, and TUBULIN (loading control) protein levels in *E4f1*^cKO^ or CTL Mefs at the indicated time points after induction of adipocyte differentiation. Histobars represent the quantification of immunoblots (arb. units, arbitrary units, *n* = 4 independent populations of cells/group). For experiments described in **a**, **b**, and **c**, cells were transduced with a Cre-encoding retrovirus 5 days prior to the induction of adipocyte differentiation. **d** RT-qPCR analysis of *E4f1*, *Fasn,* and *Acc* mRNA levels upon *E4f1* inactivation in differentiated adipocytes. RNAs were prepared 10 days after transduction with a GFP- or a Cre- encoding adenovirus in cells that were previously committed to adipocyte differentiation for 7 days (*n* = 4 independent populations of cells/group). **e** Immunoblot analysis of FASN, ACC, and ACTIN (loading control) protein levels in the same cells than in **d**. **f** De novo lipogenesis (DNL), measured after incubation of *E4f1*^cKO^ or CTL Mefs with ^14^C-acetate, 10 days after induction of adipocyte differentiation. ^14^C incorporation in tri- di- and mono- acylglycerols (TG, DG, MG, respectively), free fatty acids (FA), and phospholipids (PL) was determined by thin-layer chromatography (arb. units, *n* = 5 independent populations of cells/group). **g** FAO (nmol/mg/h) in *E4f1*^*cKO*^ and CTL Mefs evaluated upon incubation with ^3^H-palmitate, 7 days after induction of adipocyte differentiation (*n* = 4 independent populations of cells/group). Molecular weights are indicated in kDa. Data  are presented as mean ± standard error mean (SEM) from the indicated number of independent samples. Statistical analyses were performed using two-sided non-parametric Mann–Whitney *U* tests and the BiostaTGV software (ns, not significant). Source data are provided as a Source Data file.

To further characterize the metabolic perturbations in *E4F1*-deficient adipocytes, we designed a microfluidic-based RT-qPCR approach to assess the expression profile of 67 genes implicated in lipid metabolism and transport, fatty acid oxidation (FAO), glycolysis, glutaminolysis, PPP, and mitochondrial biogenesis. *E4f1*^cKO^ adipocytes displayed decreased mRNA levels of genes encoding several key enzymes involved in de novo lipid synthesis, including *Acetyl-CoA-carboxylase* (*Acc*), *Fatty acid synthase* (*Fasn*), and *Steaoryl-Coenzyme A desaturase* 1 (*Scd1*). In contrast, the induction of other genes related to lipid metabolism during adipocyte differentiation, including *Acly* and *Lcad*, was comparable in *E4f1*^cKO^ and *CTL* cells (Fig. 4a). The decreased induction of *Acc* and *Fasn* in *E4f1*^cKO^ Mefs was confirmed by conventional RT-qPCR as well as by quantitative immunoblotting (Fig. 4b, c). Notably, the expression of these genes, but not that of *Pparγ* and *Fapb4*, was also affected when *E4f1* inactivation was triggered in fully differentiated Mefs (Fig.  4d, e and Supplementary Fig. S4d), indicating that their deregulation was not a consequence of impaired differentiation. Next, we evaluated whether these changes impacted de novo lipid synthesis. We performed in vitro radiotracer accumulation assays in *E4f1*^cKO^ Mefs using ^14^C-labeled acetate as a source of lipogenic AcCoA. *E4f1*^cKO^ differentiated adipocytes incorporated 50% less ^14^C into the major lipid fractions including mono-, di- and triglycerides (MG, DG, TG, respectively), FFA, and phospholipids (PL), indicating that *E4f1* inactivation impacted on de novo lipid synthesis (Fig. 4f). Moreover, and consistent with the increased mRNA levels of *Cpt1a/c*, two genes implicated in lipid oxidation, *E4f1*^cKO^ adipocytes displayed increased FAO, as measured by the production of ^3^H2O upon incubation with ^3^H-labeled palmitate (Fig. 4a, g). Hence, these data indicate that E4F1 deficiency in adipocytes impairs de novo lipid synthesis and concomitantly increases FAO, thereby impacting on lipid accumulation.

### Lipid metabolism defects in *E4f1*^cKO^ adipocytes are p53-dependent

**Fig. 5:**
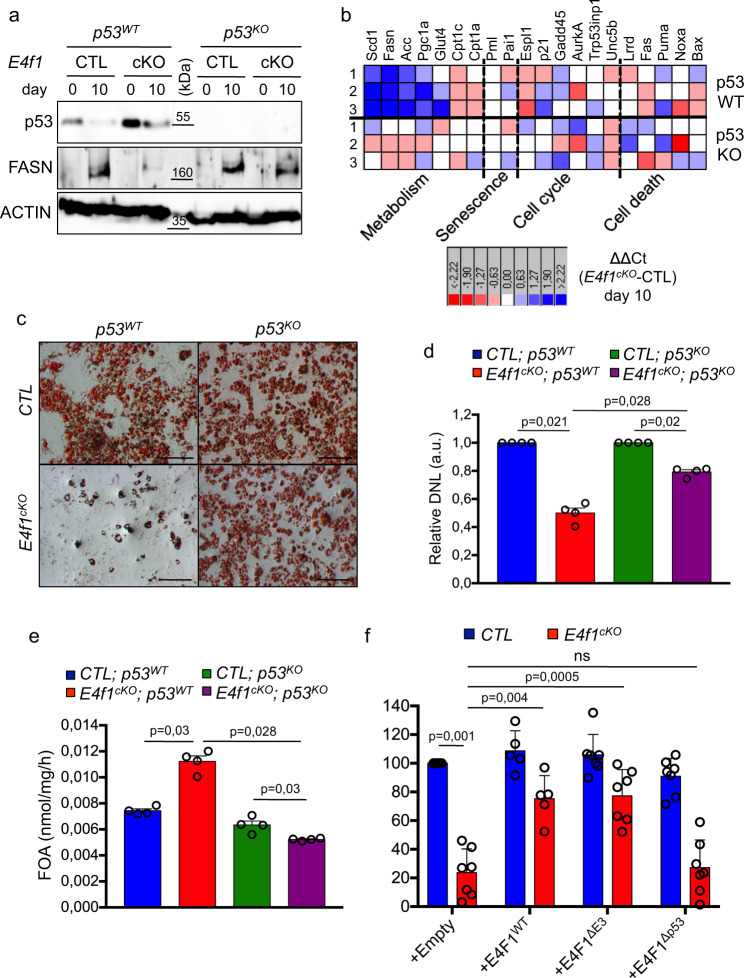
Lipid metabolism defects resulting from E4F1 deficiency are p53 dependent. **a** Immunoblot analysis of p53, FASN, and ACTIN (loading control) protein levels in total protein extracts prepared from *E4f1*^*cKO*^ and CTL Mefs, and their matched *p53*^KO^ counterparts, before or 10 days after induction of adipocyte differentiation (Data are representative of *n* = 4 independent populations/group). **b** mRNA levels of a subset of p53-target genes determined by a microfluidic RT-qPCR approach in *E4f1*^*cKO*^ and CTL Mefs, and their matched *p53*^KO^ counterparts. The heat map represents the relative mRNA levels of the indicated genes, 10 days after induction of adipocyte differentiation, calculated using the ΔΔCt method between *E4f1*^*cKO*^ and CTL Mefs. Each line represents an independent experiment performed on paired samples. **c** Representative microphotographs at low-magnification of *E4f1*^*cKO*^ and CTL Mefs, and their matched *p53*^KO^ counterparts, upon Oil Red O (ORO) staining, 10 days after adipocyte differentiation (*n* = 5 independent populations of Mefs/group). Scale bars, 200 μm. **d** De novo lipogenesis (DNL) was measured after incubation of *E4f1*^cKO^ or CTL Mefs, and their matched *p53*^KO^ counterparts with ^14^C-acetate. ^14^C incorporation in total lipids was determined 7 days after induction of adipocyte differentiation (arb. units, arbitrary units; *n* = 4 independent populations of Mefs/group). **e** Fatty acid oxidation (FAO, nmol/mg/h) upon incubation of *E4f1*^*cKO*^ and CTL Mefs, and their matched *p53*^KO^ counterparts, with ^14^C-palmitate, 7 days after induction of adipocyte differentiation (*n* = 4 independent populations of Mefs/group). **f** *E4f1*^cKO^ or CTL Mefs were transduced with retroviruses encoding wild-type E4F1 (E4F1^WT^) or E4F1 mutants deleted of its E3 ligase (E4F1^ΔE3^) or p53 interaction (E4F1^Δp53^) domains. Histobars represent neutral lipids accumulation in these complemented cells determined upon ORO staining, 10 days after induction of adipocyte differentiation (*n* = 5 independent populations of Mefs/group). Molecular weights are indicated in kDa. Data are presented as mean ± standard error mean (SEM) from the indicated number of independent samples. Statistical analyses were performed using two-sided non-parametric Mann–Whitney *U* tests and the BiostaTGV software (ns, not significant). Source data are provided as a Source Data file.

We noticed that the mRNA levels of *p53*, as well as those of several previously described p53 target genes linked to metabolism including *Fasn*, *Scd1*, *Glut4*, *Pgc1α,* and *Cpt1c*, were deregulated in *E4f1*^cKO^ adipocytes^11,13,35–38^. Given the multiple connections between E4F1 and the p53 pathway, we next investigated the potential implication of p53 in the metabolic phenotypes of *E4f1*^cKO^ adipocytes. Consistent with this hypothesis, we found that *E4f1*^cKO^ cells displayed an altered expression pattern of p53 protein. Indeed, while p53 protein levels declined at day 10 after adipocyte differentiation in *E4f1*^*CTL*^ cells, its expression was sustained in *E4f1*^*cKO*^ adipocytes (Fig. 5a). Importantly, in E4F1-deficient adipocytes, p53 expression profile did not correlate with increased levels of phosphorylated H2AX on serine 139 (γH2AX), a hallmark of DNA damage (Supplementary Fig. S5a, b). The absence of DNA damage in E4F1-deficient adipocytes was further confirmed on tissue sections prepared from *E4f1*^(adipoQ)KO^, *E4f1*^(aP2)KO^, and *E4f1*^(RERT)KO^ mice (Supplementary Fig. S5a, b). Furthermore, we detected no difference in the intracellular levels of reactive oxygen species (ROS), in the total amount of carbonylated-proteins (Oxyblot), or in the ROS-detoxifying enzyme catalase, between E4F1-proficient and -deficient Mefs during adipocyte differentiation (Supplementary Fig. S5c–e). These data supported the notion that the deregulation of p53 expression in *E4f1*^cKO^ adipocytes occurred independently of genotoxic insults or oxidative stress. To further characterize this p53 response, we measured the mRNA levels of 22 known p53 target genes involved in cell proliferation, cell death, senescence, and metabolism in *E4f1*^flox^*; p53*^WT^ Mefs and their matched *E4f1*^flox^*; p53*^KO^ counterparts upon transduction with control or Cre-encoding retrovirus. Strikingly, in p53-proficient cells, the mRNA levels of p53 target genes involved in metabolism (*Fasn*, *Scd1*, *Glut4*, *Pgc1α, Cpt1a/c*), but not those of genes falling into the senescence, cell proliferation or cell death categories, were deregulated upon *E4f1* inactivation (Fig. 5b). Their expression pattern was rescued in p53-deficient cells, irrespective of whether these genes were upregulated (*Cpt1a/c*) or downregulated (*Fasn*, *Scd1*, *Glut4*, *Pgc1α*) upon *E4f1* inactivation, confirming that their deregulation occurred in a p53-dependent manner (Fig. 5a, b).

Consistent with previous reports, p53 deficiency increased lipid accumulation during adipocyte differentiation in vitro^39,40^. More importantly, *p53* inactivation rescued TG accumulation in E4F1-deficient adipocytes to a level comparable to that of their matched control cells. p53 inactivation also restored significantly de novo lipid synthesis and normalized FAO in these cells (Fig. 5c–e). Because we previously showed that E4F1 regulates p53 at the post-translational level by controlling its ubiquitylation^20^, we next evaluated whether E4F1 and p53 physical interaction, as well as E4F1 E3 ligase activity, were required for lipid metabolism in adipocytes. We transduced *E4f1*^cKO^ Mefs with retroviruses encoding either full-length wild-type E4F1 (E4F1^WT^) or mutants lacking its E3 ligase (E4F1^ΔE3^) or p53 interaction (E4F1^Δp53^) domains prior to the induction of adipocyte differentiation^20,22^. The inability of the E4F1^Δp53^ mutant to interact with endogenous p53 in Mefs was verified by co-immunoprecipitation experiments (Supplementary Fig. S6a). Expression of ectopic E4F1^WT^ or E4F1^ΔE3^ proteins in *E4f1*^cKO^ cells restored TG accumulation, as shown by ORO staining performed 10 days after adipocyte differentiation, indicating that E4F1 E3 ligase activity is dispensable for proper lipid homeostasis in adipocytes. In the same experimental setting, the E4F1^Δp53^ mutant failed to rescue TG accumulation in *E4F1*^cKO^ adipocytes despite this mutant being expressed at higher levels than E4F1^WT^ or the E4F1^ΔE3^ proteins in these cells (Fig. 5f and Supplementary Fig. S6b). These data support the notion that p53 direct interaction with E4F1, but not its ubiquitylation, contributes to lipid metabolism in adipocytes.

### *p53* inactivation rescues the metabolic phenotypes of *E4f1*^(*aP2*)*KO*^ mice

**Fig. 6:**
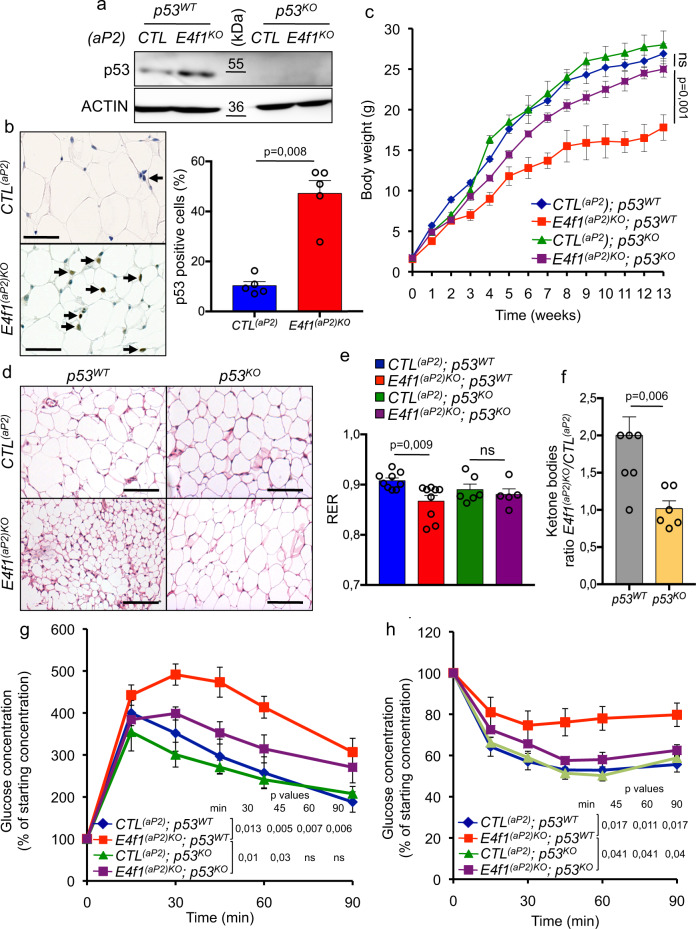
*p53* inactivation rescues adiposity and lipid metabolism defects of *E4f1*^(*aP2*)KO^ mice. **a** Immunoblot analysis of p53 and ACTIN (loading control) protein levels in epidydimal white adipose tissue (WATe) of 8-week-old *E4f1*^(*aP2*)KO^ and *CTL*^*(aP2)*^ mice, and their matched *p53*^KO^ littermates. Data are representative of five independent experiments using paired samples. **b** Representative microphotographs of immunohistochemistry (IHC) analysis of p53 protein levels in WATe of 8–12-week-old *E4f1*^(*aP2*)KO^ and *CTL*^*(aP2)*^ mice. Scale bar, 200 μm. Arrows indicate p53-positive cells. Histobars represent the percentage of p53-positive adipocytes assessed by IHC in WATe tissue sections prepared from *E4f1*^(*aP2*)KO^ and *CTL*^*(aP2)*^ mice (*n* = 5 animals/group, minimum of 100 cells counted per section). **c** Body weight of *E4f1*^(*aP2*)KO^ and *CTL*^*(aP2)*^ mice, and their matched *p53*^KO^ littermates, under normal chow diet (*n* = 8 males/group). **d** Representative microphotographs of hematoxylin and eosin (H&E)–stained sections of WATe prepared from *E4f1*^(*aP2*)KO^ and *CTL*^*(aP2)*^ mice, and their matched *p53*^KO^ littermates. Scale bars, 500 μm. **e** Respiratory exchange ratio (RER) of *E4f1*^(*aP2*)KO^ and *CTL*^*(aP2)*^ mice, and their matched *p53*^KO^ littermates, determined during 24 h (*n* = 5 males/group). **f** Levels of circulating ketone bodies in *E4f1*^(*aP2*)KO^ and *CTL*^*(aP2)*^ mice, and their matched *p53*^KO^ littermates (represented as the ratio between *E4f1*^(*aP2*)KO^ and *CTL*^*(aP2)*^ mice in *p53*^WT^ or *p53*^KO^ mice; *n* = 6 animals/group). **g**, **h** IPGTT (**g**) and ITT (**h**) performed on *E4f1*^(*aP2*)KO^ and *CTL*^*(aP2)*^ mice (*n* = 7 males/group) and their *p53*^KO^ counterparts (*n* = 5 males/group). Molecular weights are indicated in kDa. Data are presented as mean ± standard error mean (SEM) from the indicated number of independent samples. Statistical analyses were performed using two-sided non-parametric Mann–Whitney *U* tests and the BiostaTGV software (ns, not significant). Source data are provided as a Source Data file.

Consistent with the role of p53 in the metabolic defects observed in E4F1-deficient adipocytes, the protein levels of p53 increased in the WATe of *E4f1*^(*aP2*)*KO*^ and *E4f1*^(*adipoQ)KO*^ mice (Fig. 6a, b and supplementary Fig. S6d). Because of the mosaic recombination of the *E4f1*^flox^ allele in *E4f1*^(*adipoQ)KO*^ mice, we then mated *E4f1*^(*aP2*)*flox*^ and *p53*^*KO*^ mice to further confirm the role of *p53* in these metabolic phenotypes in vivo. RT-qPCR analyses showed that the mRNA levels of several lipogenic and FAO-related genes that were deregulated upon *E4f1* inactivation, including *Acc, Fasn*, *Scd1,* and *Cpt1a/c*, were comparable in WATe of *E4f1*^(*aP2*)*KO*^; *p53*^*KO*^ mice and *CTL*^(*aP2*)^; *p53*^*KO*^ control littermates (Supplementary Fig. S6e). Moreover, the inactivation of *p53* significantly restored adiposity and the weight of animals lacking E4F1 in their WAT (Fig. 6c). Histological analyses indicated that the mean size of white adipocytes was similar in *E4f1*^(*aP2*)*KO*^; *p53*^KO^ mice and *CTL*^(*aP2*)^; *p53*^KO^ control littermates (Fig. 6d). Moreover, animals from these two experimental groups displayed comparable RER and plasma levels of ketone bodies (Fig. 6e, f). Finally, *p53* inactivation significantly improved the insulin resistance and glucose intolerance observed in *E4f1*^(*aP2*)*KO*^ males (Fig. 6g, h). Taken together, these data demonstrate that E4F1 deficiency impacts on adipocyte functions in a p53-dependent manner both in vitro and in vivo.

### The E4F1-p53-SCD1 axis plays an important role in the generation of monounsaturated fatty acids (MUFAs) and WAT adipocyte functions

**Fig. 7:**
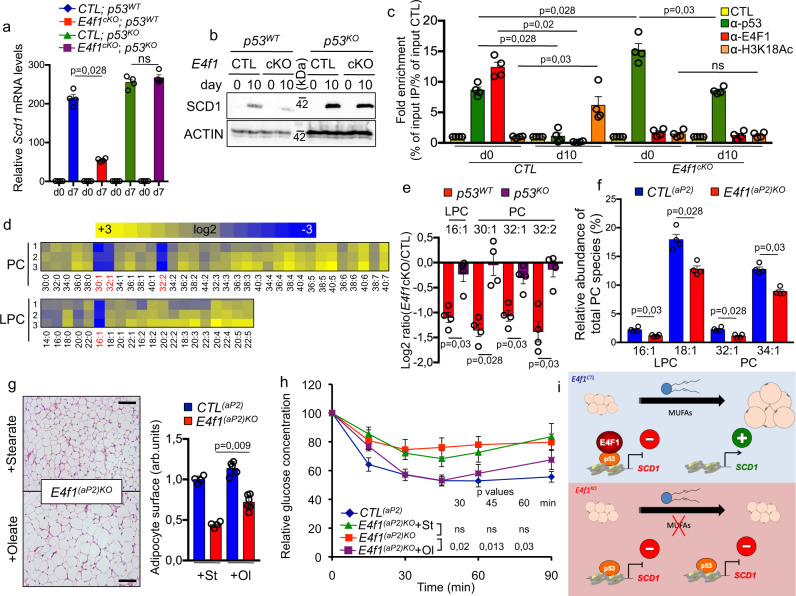
The E4F1-p53-SCD1 axis plays an important role in the generation of MUFAs and white adipocytes functions. **a** RT-qPCR analysis of *Scd1* mRNA levels in *E4f1*^cKO^ or CTL Mefs, and in their *p53*^KO^ counterparts, at the indicated time points after induction of adipocyte differentiation (*n* = 4 independent populations of cells/group). **b** Immunoblot analysis of SCD1 and ACTIN (loading control) protein levels in total protein extracts prepared from *E4f1*^cKO^ or CTL Mefs and their *p53*^KO^ counterparts, at the indicated time points after induction of adipocyte differentiation (data are representative of 3 independent experiments). **c** Quantitative chromatin immunoprecipitation (qChIP) experiments performed on the *Scd1* promoter with anti- E4F1, p53, H3K18ac or control antibodies and chromatin prepared from *E4f1*^cKO^ or CTL Mefs, at the indicated time points after induction of adipocyte differentiation. Results are represented as the relative ratio between the mean value of immunoprecipitated chromatin (calculated as a percentage of the input) with the indicated antibodies and the one obtained with a control irrelevant antibody (*n* = 4 independent populations of cells/group). **d** Semi-quantitative analysis of different lysophosphatidycholine (LPC) and phosphatidylcholine (PC) species by ESI-MS-MS in *E4f1*^*cKO*^ and CTL Mefs, 10 days after induction of adipocyte differentiation. The heat maps show the relative abundance of the indicated LPC and PC, expressed as log2 of the ratio of the percentages of total PC between *E4f1*^cKO^ and CTL cells. Each lane represents an independent experiment performed on paired samples (*n* = 3). **e** Histobars represent the relative abundance of the indicated PC relevant to SCD1 activity in *E4f1*^*cKO*^ and CTL Mefs, and their *p53*^KO^ counterparts (expressed as the log2 ratio of the percentage of total PC between *E4f1*^*CTR*^ and *E4f1*^*cKO*^ cells. *n* = 3 independent populations of cells/group). **f** Histobars represent the relative abundance of the indicated PC in BAT harvested from *E4f1*^*(aP2)KO*^ and *CTL*^*(aP2)*^ littermates (*n* = 4 animals/group). **g** Representative microphotographs of hematoxylin and eosin (H&E)-stained WATe sections prepared from *E4f1*^*(aP2)KO*^ and *CTL*^*(aP2)*^ littermates fed with a tristearin (St) or triolein (Ol)-complemented diet for 3 weeks. Scale bars, 500 μm. Histobars (right panel) represents the quantification of adipocyte surface area of WATe from these animals (arb. units, arbitrary units; *n* = 4 animals/group). **h** Insulin tolerance test performed on *E4f1*^*(aP2)KO*^ mice and *CTL*^*(aP2)*^ littermates, fed with a St or Ol -complemented diet (*n* = 7 males/group). **i** Schematic representation of the roles of E4F1 and p53 on *Scd1* regulation during adipocyte differentiation. Molecular weights are indicated in kDa. Data were presented as mean ± standard error mean (SEM) from the indicated number of independent samples. Statistical analyses were performed using two-sided non-parametric Mann–Whitney *U* tests and the BiostaTGV software (ns, not significant). Source data are provided as a Source Data file.

Interestingly, the *Scd1* gene, which encodes the SCD1 enzyme that catalyzes the synthesis of Δ9 MUFAs, was among the most strongly downregulated metabolic genes in *E4f1*^*cKO*^ adipocytes. Immunohistochemistry (IHC) analyses of WAT sections prepared from *E4f1*^*(adipoQ)KO*^ and *CTL*^*(adipoQ)*^ mice showed that *E4f1* deficiency in differentiated adult adipocytes resulted in a more patchy expression pattern of SCD1 protein (Supplementary Fig. S7a). Moreover, *E4f1*^*(adipoQ)KO*^ mice exhibited decreased *Scd1* mRNA levels (Supplementary Fig. S7b). Analysis of *Scd1* mRNA and protein levels in *E4f1*^*flox*^*; p53*^*KO*^ Mefs and matched control cells showed that the downregulation of *Scd1* in *E4f1*^*cKO*^ adipocytes occurred in a p53-dependent manner (Fig. 7a, b). As p53 was previously found to bind a CpG island located in the human *SCD1* locus^35^, we tested if both E4F1 and p53 contributed directly to the control of *Scd1* expression in adipocytes. To answer this question, we performed quantitative chromatin immunoprecipitation (qChIP) experiments using previously validated E4F1 and p53 antibodies^26,41^ and chromatin prepared from Mefs committed to adipocyte differentiation. The specificity of these E4F1 and p53 ChIP signals was confirmed in *E4f1*^*cKO*^ and *p53*^*KO*^ Mefs (Fig. 7c and Supplementary Fig. S7c). In *CTL* Mefs, endogenous E4F1 and p53 proteins were both detected on the promoter of the murine *Scd1* locus in undifferentiated Mefs but their binding was lost 10 days after the addition of the differentiating cocktail. Strikingly, p53 binding to the *Scd1* locus was maintained in *E4f1*^*cKO*^ Mefs 10 days after adipocyte differentiation was triggered, correlating with the impaired transcriptional activation of *Scd1* in E4F1-deficient adipocytes (Fig. 7c). A survey of several epigenetic marks (H3K4me3, H3K9me3, H3K27me3, H3K27Ac, H3K18Ac) by qChIP showed that the H3K4me3 and H3K18Ac marks were significantly enriched in this region of the *Scd1* locus in differentiated adipocytes (Supplementary Fig. S7d). These qChIP analyses also indicated that the binding of p53 to the *Scd1* locus inversely correlated with the acetylation profile of H3K18ac in E4F1-deficient adipocytes (Fig. 7c). Furthermore, and consistent with our previous findings, expression of ectopic E4F1^WT^ or E4F1^ΔE3^ proteins in *E4f1*^*cKO*^ adipocytes, but not of the E4F1^Δp53^ mutant, restored *Scd1* expression (Supplementary Fig. S7e). A similar rescue was also observed for *Cpt1c* and *InsR*, two other p53-target genes deregulated in E4F1-deficient adipocytes. Of note, expression of the E4F1-p53 interaction mutant in E4F1-deficient Mefs restored efficiently the expression of *Dnajc19*, a p53-independent target gene of E4F1, excluding the possibility that its inability to rescue *Scd1* mRNA levels in these cells resulted from impaired transcriptional activities (Supplementary Fig. S6c)^26^. Thus, these data support a model where E4F1 deficiency leads to the maintenance of p53 binding to the *Scd1* locus and impairs its epigenetic regulation during adipocyte differentiation.

These results prompted us to investigate the importance of deregulated expression of *Scd1* in E4F1-deficient adipocytes and its consequences on MUFAs synthesis. We transduced *E4f1*^cKO^ cells with adenoviral particles encoding *SCD1* or *GFP* as a control. Although the level of ectopic SCD1 protein was inferior to that of endogenous SCD1 in differentiated adipocytes, it partly rescued TG accumulation in E4F1-deficient adipocytes, as shown by ORO staining (Supplementary Fig. S7f, g). To confirm the role of SCD1 and MUFAs in these metabolic phenotypes, we next performed a detailed analysis of PL in *E4f1*^cKO^ and control adipocytes, 10 days after induction of differentiation, using a semi-quantitative lipidomic electrospray ionization tandem mass spectrometry (ESI-MS-MS) approach. These analyses revealed a significant shift in the composition of PL in *E4f1*^cKO^ cells with a decrease in the relative amounts of monounsaturated PL species relying on SCD1 activity (C16:1, C30:1, C32:1, C32:2). This effect was the strongest for phosphatidylcholine (PC) species (Fig. 7d), intermediate for phosphatidylethanolamine and phosphatidylinositol, and less pronounced for phosphatidylserine (Supplementary Fig. S8). Notably, *p53* inactivation in E4F1-deficient adipocytes restored the abundance of these MUFAs (Fig. 7e and Supplementary Fig. S7h). We next attempted to confirm the in vivo relevance of these observations. Because the limited amount of WAT tissue of *E4f1*^(*aP2*)KO^ was not amenable to ESI-MS-MS lipidomic profiling, we analyzed PC species in BAT harvested from these animals. Although the relative abundance of some PL species was different between in vitro differentiated adipocytes and BAT, a comparable decrease in the relative levels of monounsaturated PC (C16:1, C18:1, C32:1, C34:1) was detected in E4F1-deficient BAT (Fig. 7f). To further confirm the importance of MUFAs in the phenotypes of *E4f1*^(aP2)*KO*^ mice, we next complemented the diet of 3–4-week-old *E4f1*^(*aP2*)*KO*^ mice and CTL^(*aP2*)^ littermates with equal amounts of oleate (C18:1) or stearate (C18:0) for 3 weeks. Histological analyses of WAT sections prepared from these animals revealed that oleate, but not stearate, complementation increased the mean size of E4F1-deficient white adipocytes (Fig. 7g). Strikingly, the insulin sensitivity of oleate-complemented *E4f1*^(*aP2*)KO^ mice improved relative to that of stearate-complemented *E4f1*^(*aP2*)KO^ animals (Fig. 7h). Hence, these data highlight the importance of an E4F1-p53-SCD1 axis in the synthesis of MUFAs that contributes to WAT adipocyte functions (Fig. 7i).

## Discussion

Several oncogenes and tumor suppressors are now recognized as essential metabolic regulators. Although less documented than its activities in cell cycle control, apoptosis, and genome integrity, growing evidence supports the importance of the p53 tumor suppressor in metabolism. However, the mechanisms underlying p53-mediated metabolic responses remain poorly understood. Here, we identify a link between the multifunctional E4F1 protein and the p53 tumor suppressor in adipocyte differentiation and lipid metabolism that is essential for normal adipose tissue function. Moreover, our data suggest that perturbation of this regulatory mechanism of lipid homeostasis influences obesity and insulin resistance.

Using several genetically engineered mice lacking E4F1 in adipocytes, we show that *E4f1* inactivation induces a p53-mediated response implicated in lipid metabolism. The atypical metabolic profile of mice lacking E4F1 in adipose tissue was associated with insulin resistance despite these animals exhibited diminished adiposity, decreased circulating FFAs but no liver steatosis. Diabetes is a heterogeneous disease of various molecular etiologies. Although more work will be needed to identify the mechanisms by which E4F1 deficiency in adipose tissue results in insulin resistance, these mice represent an interesting animal model to study atypical forms of diabetes and highlight the importance of lipid metabolism in adipocytes in the systemic regulation of glucose homeostasis. Previous work reported that p53 induction by excessive caloric intake or shortening telomeres results in a pro-inflammatory response in adipose tissue that contributes to insulin resistance^42^. Notably, the in vivo perturbations of lipid metabolism observed upon *E4f1* inactivation in adipocytes appeared independently of an inflammatory response. In addition, we could recapitulate these defects in primary adipocytes cultured in vitro, demonstrating that they occurred in a cell-autonomous manner. Altogether, these results illustrate the important and complex roles of p53 in adipocytes.

Our extensive analysis of the p53 response in *E4f1*^*cKO*^ adipocytes indicates that, upon E4F1 deficiency, p53 specifically controls a transcriptional program implicated in metabolism but not those involved in the cell cycle, cell death, or senescence. This p53-mediated metabolic response was very specific since some, but not all, previously identified p53 target genes involved in metabolism were found to be deregulated in *E4f1*^*cKO*^ cells. This signature was concordant with the observed metabolic switch triggered by *E4f1* inactivation that included a decrease of de novo fatty acid synthesis and a concomitant increase of FAO. However, the deregulation of other p53-responsive genes implicated in glycolysis (*Glut4*), glutaminolysis (*Gls2*), PPP (*Tigar*), or OXPHOS (*Sco2*) also suggests that p53 induction in E4F1-deficient adipocytes may also influence other metabolic pathways. Interestingly, p53 was recently implicated in the regulation of the mevalonate pathway in hepatocytes through a mechanism implicating cholesterol transport by the ABCA1 transporter and SREBP2 maturation in the endoplasmic reticulum^43^. Although SREBP1c and SREBP2 can have overlapping functions in lipid metabolism, previous data suggest that they preferentially regulate different subsets of target genes involved in the fatty acid synthesis and cholesterol biosynthesis, respectively^44^. More work will be needed to evaluate whether the metabolic changes resulting from *E4f1* inactivation extend to genes involved in the mevalonate pathway in other cell types and whether the SREBP proteins contribute to these defects. Nonetheless, our data demonstrate that p53-mediated deregulation of lipid biosynthesis and FAO is central to the metabolic reprogramming of *E4f1*^cKO^ adipocytes.

Our data raise interesting questions regarding the defects leading to p53 deregulation in *E4f1*^*cKO*^ cells. Indeed, we failed to detect any classical hallmarks of DNA damage or oxidative stress. It is noteworthy that E4F1 regulates mitochondrial activities independently of p53^26,29^, suggesting that metabolic perturbations occurring upon E4F1 deficiency may represent the initial event that triggered this p53-dependent metabolic response. Our results suggest that the direct recruitment of E4F1 and p53 on the *Scd1* locus is required for its proper epigenetic regulation in adipocytes. Although this question extends beyond the scope of this manuscript, an interesting possibility is that E4F1 controls the recruitment of specific p53-transcriptional co-factors on some of its target genes to induce adaptive responses to metabolic challenges. This hypothesis is consistent with our previous data showing that the multifunctional protein E4F1 functions as a router for p53. However, the ubiquitylation of p53 on lysine K320 by E4F1, which we originally identified as one of the molecular mechanisms specifying p53-mediated cell cycle arrest^20^, appears dispensable for p53-mediated control of lipogenic genes. Nevertheless, our data indicate that the direct physical interaction between E4F1 and p53 is essential for the proper control of lipid metabolism in adipocytes. Interestingly, acetylation of lysines 98, 117, 136, 161, and 162 that are located in the DNA binding domain of p53, was previously shown to be important for the differential regulation of p53-target genes, including those involved in metabolism and mTOR signaling^45–47^. In view of these intriguing findings, it is tempting to speculate that the recruitment of E4F1 and p53 to some of these metabolic genes relies on specific, yet unidentified, post-translational modifications of p53.

Our in vitro and in vivo data support the notion that the imbalanced ratio between saturated and monounsaturated FAs that resulted from *Scd1* deregulation plays an important role in the metabolic phenotypes of E4F1-deficient adipocytes. However, the phenotypic differences between mice lacking *Scd1* and *E4f1* in adipose tissue and the expression profile of E4F1-deficient adipocytes indicate that the transcriptional program controlled by p53 upon *E4f1* inactivation extends beyond *Scd1*^48^. Altogether, our data highlight the importance of this E4F1-p53-SCD1 axis in adipose tissue function and its implication in obesity and insulin resistance. These observations pave the way for future studies aiming at further understanding the complex interplay between E4F1 and p53 in metabolic diseases and cancer progression.

## Methods

### Animal treatment, Oxylet, cold test, IPGTT, and GTT

The following strains were used in this study: E4F1 < tm1.1Llca > (MGI:4867860), E4F1 < tm1Pisc > (MGI:3050154), Tg(Adipoq-cre/ERT2)1Soff/J (MGI:5568125), Polr2a < tm1(cre/ERT2)Bbd > (MGI:3772332), Tg(Fabp4-cre)1Rev (MGI:2386686), Trp53 < tm1Tyj > (MGI:1857263), Lep<Ob > (MGI:1856424). These strains were interbred and maintained on a mix 129 Sv/J; C57Bl/6 background and housed in a pathogen-free barrier facility (room temperature 22 °C; relative humidity 55%, and a 12-h-light–dark cycle). All procedures were approved by the ethic committee for animal warefare of the region Languedoc Roussillon (Comité d’Ethique en Expérimentation Animal Languedoc Roussillon) which is an accredited institution of the french “MINISTERE DE L’ENSEIGNEMENT SUPERIEUR, DE LA RECHERCHE ET DE L’INNOVATION” (agreement numbers #CEEA-LR-12116 and #17078-20181009101330v2). Animal housing and euthanasia were performed in accordance with the 3 R rule and with recommendations of the Guide for the Care and Use of Laboratory Animals. Mice were maintained under chow (A03, Safe) containing 22 kcal% protein, 65 kcal% carbohydrates, and 13 kcal% fat, complemented with 20% tristearin or triolein (Safe) for rescue experiments; or high-fat diet (Van Heek DIO 60%, Genobios) containing 20 kcal% protein, 20 kcal% carbohydrates, and 60 kcal% fat. Tamoxifen was administered in 8 to 12-week-old adult *E4f1*^(*RERT*)*KO*^ and in 8-week-old *E4f1*^(*adipoQ*)*KO*^ mice and their respective control littermates by repeated topical skin applications (2 mg/day/mouse; Sigma-Aldrich, 2 to 4 administrations per week). Weight was measured weekly. Rates of O_2_ consumption and RER were determined during 24 h in Oxylet chambers (Columbus Instruments) after 8- to 12-week-old males acclimated overnight either at RT or at thermoneutral temperature (29°C). For insulin resistance (ITT) and glucose tolerance (IPGTT) tests, 8–12-week-old males were fasted 4hrs or overnight, respectively, and injected intraperitoneally with 0,75U/kg of insulin or with 2 g/kg of glucose. Glucose concentration was measured with a blood glucometer (Accu-Chek, Roche). Ketone bodies in the serum were measured using blood β-ketone strips (Optium, Abbott). The serum concentration of triglycerides, cholesterol, HDL, and LDL was measured on an automated biochemical apparatus (Pentra 400, HORIBA ABX), according to the manufacturer’s instructions.

### Genotyping

Mice were genotyped by PCR on tail genomic DNA using Red-N extract kit (Sigma), with the following primers:

*E4f1* WT and Flox alleles (*E4f1*^*flox*^): 5′-CCTTGAGCACGGAGGAGAGC-3′ and 5′-GCCCTAGCCTGCTC-TGCCATC-3′; *E4f1*^*KO*^ (E4F1^-^): 5′-CACTGCCTTGGAGGACTTTG-3′ and 5′-CCTCTGTTCCACATACACTTCATTC-3′.

*RERT* WT and knock-in alleles: 5′-GTCAGTACACATACAGACTT-3′, 5′-TGAGCGAACAGGGCGAA-3′ and 5′-TCCATGGAGCACCCAGTGAA-3′.

*aP2-Cre* allele: 5′-GCGGTCTGGCAGTAAAAACTATC-3′ and 5′-GTGAAACAGCATTGCTGTCACTT-3′.

*AdipoqCreER*^*T2*^ allele: 5′-TGGTGCATCTGAAGACACTACA-3′ and 5′-TGCTGTTGGATGGTCTTCACAG-3′.

*p53* ^WT^ and ^KO^ alleles: Fwd 5′-CCATGCAGGAGCTATTACACA-3′; Rev WT:5′-AGCGTGGTGGTACCTTATGAG-3′, Rev KO: 5′- GCTATCAGGACATAGCGTTGG-3′.

### Patients and clinical material

All patients underwent cholecystectomy at the Virgen de la Victoria Hospital (Malaga, Spain) between August and September 2014. Samples of visceral fat depots were collected from patients who signed a written informed consent authorizing their participation in the study and publication of potentially identifiable personal clinical information. This work was conducted according to the Declaration of Helsinki principles and was approved by the Research Ethics Committee of Malaga (agreement number PI 12/02355).

### ARN extraction, quantitative PCR, and microfluidic analysis

Total RNAs were isolated using TriZol Reagent (Invitrogen). cDNAs were synthesized from 500 ng of total RNA using SuperScript™ III Reverse Transcriptase (Invitrogen). Quantitative real-time PCR was performed on a LightCycler 480 (Roche). For microfluidic analyses, after a pre-amplification step, cDNA quantification was analyzed by PCR using the Fluidigm dynamic array on a BioMark (Fluidigm) according to the manufacturer’s recommendations. The relative mRNA copy number was calculated using Ct values and was normalized with *18* *S*, *β2microglobulin*, and *tubulin β5* RNA. Primers used for microfluidic analyses are provided in supplementary table 1.

### Protein extraction, immunoprecipitation, and western blotting

For immunoprecipitation experiments, cells were lysed in buffer (100 mM Tris-HCl pH 8, 100 mM NaCl, 1 mM EDTA, 1% NP-40, 1 mM DTT, protease inhibitors (Complete, Roche)). 1 mg of total cellular protein was incubated overnight at 4 °C with 1 μg of anti-p53 antibody (1C12, Cell Signalling) and protein G-Dynabeads (Life sciences). IP extracts were analyzed by immunoblotting with anti-p53 (1C12, Cell Signaling, 1/1000) and anti-E4F1 (1/2000) antibodies^21^. For total protein extracts, cells were lysed in Laemmli buffer (80 mM Tris pH=6,8, 2% SDS, 12% sucrose, 2% β-mercaptoethanol, bromophenol blue) and immunoblotting was performed using the following antibodies: anti- SCD1 (Cell Signaling, 1/1000), FASN (Santa Cruz, 1/1000), ACC (Cell Signaling, 1/1000), Catalase (Santa Cruz, 1/1000), PPARγ (Santa Cruz, 1/500), C/EBPα (Santa Cruz, 1/1000), aP2 (Santa Cruz, 1/1000), γH2AX (Millipore, 1/1000) and ACTIN (Sigma, 1/7000). Oxyblots were carried out according to the manufacturer’s instructions (Millipore). Adobe Photoshop CS4 was used to crop images from unprocessed images. Uncropped images are provided in the Source Data file.

### Cell culture, adipocyte differentiation, and ORO staining

Mefs were isolated from E13.5 *E4f1*^*-/flox*^ embryos and cultured in DMEM complemented with 10% of heat-inactivated fetal bovine serum (FBS, *Hyclone*). Primary pre-adipocytes were isolated from WAT from 8-week-old *E4f1*^*(RERT)*^ animals and cultured in DMEM/F12 + 10% FBS. Mefs and pre-adipocytes were differentiated into adipocytes with 1 mM dexamethasone, 1,7 mM insulin, 0.5 mM IBMX, and 1 mM rosiglitazone for 3 days, followed by supplemental 1.7 μM mg/ml insulin and 1 mM rosiglitazone for an additional 4–17 days^49^. Cells were then fixed in 4% paraformaldehyde for 15 min at RT and stained with ORO solution for 15 min at RT. Triglyceride content was quantified at day 10 upon extraction with isopropanol and measuring absorbance at 510 nm.

### De novo fatty acid synthesis, lipid profiling, and fatty acid oxidation

For de novo lipid synthesis, cells were serum-starved overnight and then incubated at 37 °C for 2 h with 5 mM ^14^C-labeled acetate (1 µCi/ml–Perkin Elmer). The incorporation of ^14^C in the total lipid fraction was measured after lipid extraction following the Folch method^50^. De novo incorporation of ^14^C in the major lipid species was analyzed after lipid separation by thin-layer chromatography on Silica Gel plates developed in Heptane:Isopropylether:Acetic acid mixture (60:40:4) for 1 h, visualized by iodine vapor, quantitatively scraped from the plate and analyzed by liquid scintillation counting. Results from metabolic measurements were normalized to the total protein content of cell extracts. Analyses of PL species were performed by ESI-MS-MS^51^. For measurement of FAO, Mefs were incubated with ^3^H-labeled palmitate (coupled to BSA) and 1 mM carnitine (Sigma) for 2 hr at 37 °C. ^3^H2O produced during FAO was purified on DOWEX columns (Sigma) after TCA extraction and NaOH neutralization. Radioactivity was measured with a wallac reader (Trilux).

### Quantitative chromatin immunoprecipitation (qChIP) assays

For qChIP assays in cells, Mefs were incubated with 1% formaldehyde/1% paraformaldehyde for 5 min followed by the addition of 125 mM Glycine to stop the reaction. Cells were then washed in phosphate-buffered saline, resuspended in lysis buffer (10 mM Tris pH 8, 140 mM NaCl, 0.1% SDS, 0.5% Triton X-100, 0.05% NaDoc, 1 mM EDTA, 0.5 mM EGTA, and protease inhibitors) and chromatin was sheared by sonication (epishear, Active motif). qChIPs were carried out by incubating chromatin (Input) with protein G-Dynabeads and the different antibodies: control (irrelevant IgG), affinity-purified rabbit anti-E4F1 polyclonal^21^, anti-p53 (1C12, Cell Signaling, 10 μl per IP), anti-H3K4me3 (Cell Signaling, 10 μl per IP), anti-H3H9me3 (Cell Signaling, 10 μl per IP), anti-H3K27me3 (Cell Signaling, 10 μl per IP), anti-H3K18ac (Cell Signaling, 10 μl per IP), anti-H3K27ac (Cell Signaling, 10 μl per IP) antibodies. After overnight incubation, washing, reverse cross-linking, and treatment with both RNase A and Proteinase K, proteins were removed with phenol/chloroform extraction, and DNA was recovered using the NucleoSpin Extract II kit. Input and immunoprecipitated DNA were then analyzed by QPCR using the SYBR Green Master mix on a LightCycler 480 SW 1.5 apparatus (Roche). Results are represented as a percentage of the input. Primers used for qChIP assays are provided in supplementary table 1.

### Retrovirus and lentivirus production

Retroviral particles encoding a self-excising Cre recombinase^33^, full-length E4F1^WT^, or its E3 ligase (Δ41–84, referred to as E4F1^ΔE3^) or p53 binding (Δ520–569, referred to as E4F1^Δp53^) mutants were produced in 293 T packaging cells by transient transfection using Jet-PEI reagent (Ozyme). In all, 72 hrs after transfection, viral supernatants were harvested and added on Mefs overnight in presence of polybrene (5 μg/ml, Sigma). Antibiotic selection was performed 48 h later after transduction with hygromycin (50 μg/mL, Invitrogen). SCD1 and GFP adenoviruses were purchased from Vector Biolabs. Adeno-Cre was purchased from the University of Iowa’s viral core facility.

### Immunohistochemistry, Immunofluorescence, and cell surface measurement

Tissue biopsies were either fixed in 4% neutral-buffered formalin (24 h) and paraffin-embedded or frozen in Tissue-Tek OCT (Sakura) for cryosectioning. Paraffin-embedded tissues were sectioned and processed for IHC or H&E stainings. IHC was performed on 4 μm sections using the following primary antibodies: anti- perilipin (ABR, 1/500), Caspase-3 (Cell signaling, 1/500), F4/80 (eBioscience, 1/1000), Mac2 (eBioscience, 1/1000). The revelation was performed with secondary biotinylated anti-rat or anti-rabbit antibodies and streptavidin-peroxidase complex (ABC Vectastain kit, Vector Laboratories), and the peroxidase substrate DAB (Vector Laboratories). For immunofluorescence, Alexa488-coupled secondary antibodies (anti-mouse #A21202, anti-rabbit #A21206) were diluted at 1/1000 (Invitrogen). Adipocyte surface on H/E-stained sections of WAT was quantified using ImageJ 1.43 u software.

### Statistical analysis and reproducibility

In vivo studies were performed on a sufficient number of animals per genotype with a minimum of five animals per experimental group. These data were reproducible and were represented as mean ± SEM. The in vitro data were obtained from three to five independent experiments as indicated in the figure legends.

Statistical significance was evaluated using two-sided non-parametric Mann–Whitney *U* tests with the BiostaTGV software from Sorbonne-Paris University (https://biostatg.sentiweb.fr). *P* values inferior to 0.05 were considered statistically significant.

### Reporting summary

Further information on research design is available in the Nature Research Reporting Summary linked to this article.

## Supplementary information

Supplementary Information

Reporting Summary

## Data Availability

Uncropped gels are available in the Supplementary Information file. The clinical data from patients with obesity and lean individuals are provided in Table 2. Primers list is provided in Supplementary Table 1 (in the Supplementary Information file). All data that support the findings of this study are available within the article, its Supplementary Information, or from the corresponding author upon reasonable request. A reporting summary for this article is available as a Supplementary Information file. Source data are provided with this paper.

## Source data

Source Data
