# Supplementary material for: The multifunctional protein E4F1 links P53 to lipid metabolism in adipocytes
Source: Nat Commun. 2021 Dec 2;12:7037. doi: 10.1038/s41467-021-27307-3 (PMC8639890; doi:10.1038/s41467-021-27307-3)

Supplementary figure 1

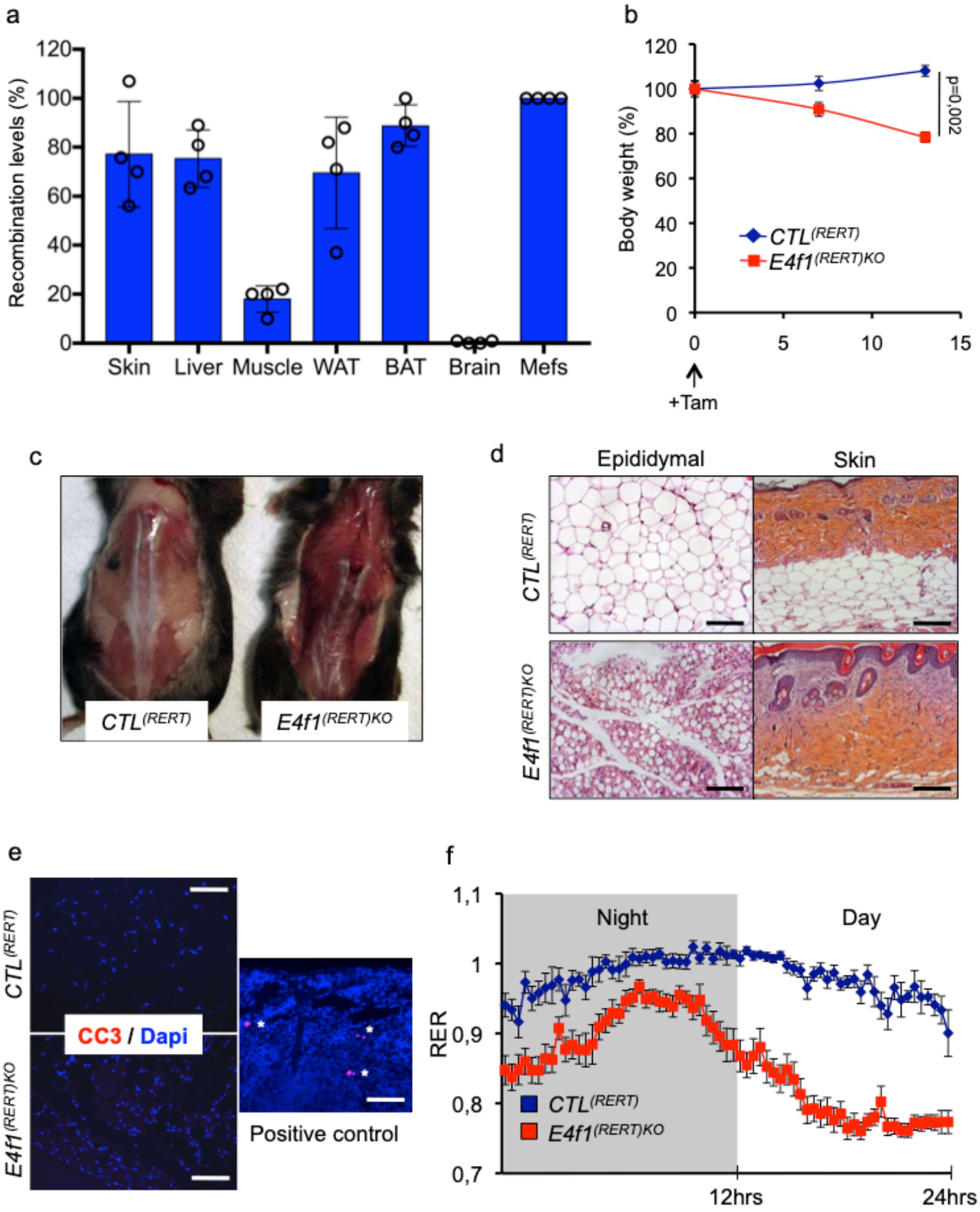

Supplementary figure 1: Characterization of *E4f1*$^{(RERT)KO}$ mice. (**a**) Efficiency of Cre-mediated recombination of the *E4f1*^{flox} allele in different organs of *E4f1*$^{(RERT)KO}$ mice, determined by qPCR on genomic DNA prepared from the indicated tissues, 72h after Tamoxifen (Tam) administration. The values were normalized to the level of recombination evaluated in *E4f1*^{cKO} Mefs (considered as 100%) (n=5 animals/group). (**b**) Body weight after Tam administration of 12-week-old *E4f1*$^{(RERT)KO}$ animals and control *CTL*$^{(RERT)}$ littermates (n=5 males/group). (**c**) Representative microphotographs of *E4f1*$^{(RERT)KO}$ and *CTL*$^{(RERT)}$ males, 13 days after Tam administration showing a strong reduction of adiposity upon acute inactivation of *E4f1* in adult animals (n=10 animals/group). (**d**) Microphotographs of hematoxylin and eosin (H&E) -stained tissue sections prepared from epididymal white adipose tissue (WATe) and subcutaneous fat (WATd) depots prepared from 8- to 12-week-old *E4f1*$^{(RERT)KO}$ males and *CTL*$^{(RERT)}$ littermates, 13 days after Tam administration (n=10 animals/group). Scale bars, 200 µm. (**e**) Immunofluorescence (IF) analysis of cleaved caspase 3 (CC3) in WATe sections prepared from 8-to-12-week-old *E4f1*$^{(RERT)KO}$ and *CTL*$^{(RERT)}$ males (n=3 animals/group). A thymus tissue section prepared from wild-type mice was used as a positive control for CC3 staining. Nuclei were counterstained with DAPI. Scale bars=500µm. (**f**) RER measured over 24 hrs in 8- to 12-week-old *E4f1*$^{(RERT)KO}$ and *CTL*$^{(RERT)}$ males, 13 days after tam administration (n=7 males/group). Data were presented as mean ± standard error mean (SEM) from the indicated number of animals. Statistical analyses were performed using two-sided non-parametric Mann-Whitney U tests and the BiostaTGV software (ns, not significant). Source data are provided as a Source Data file.

Supplementary figure 2

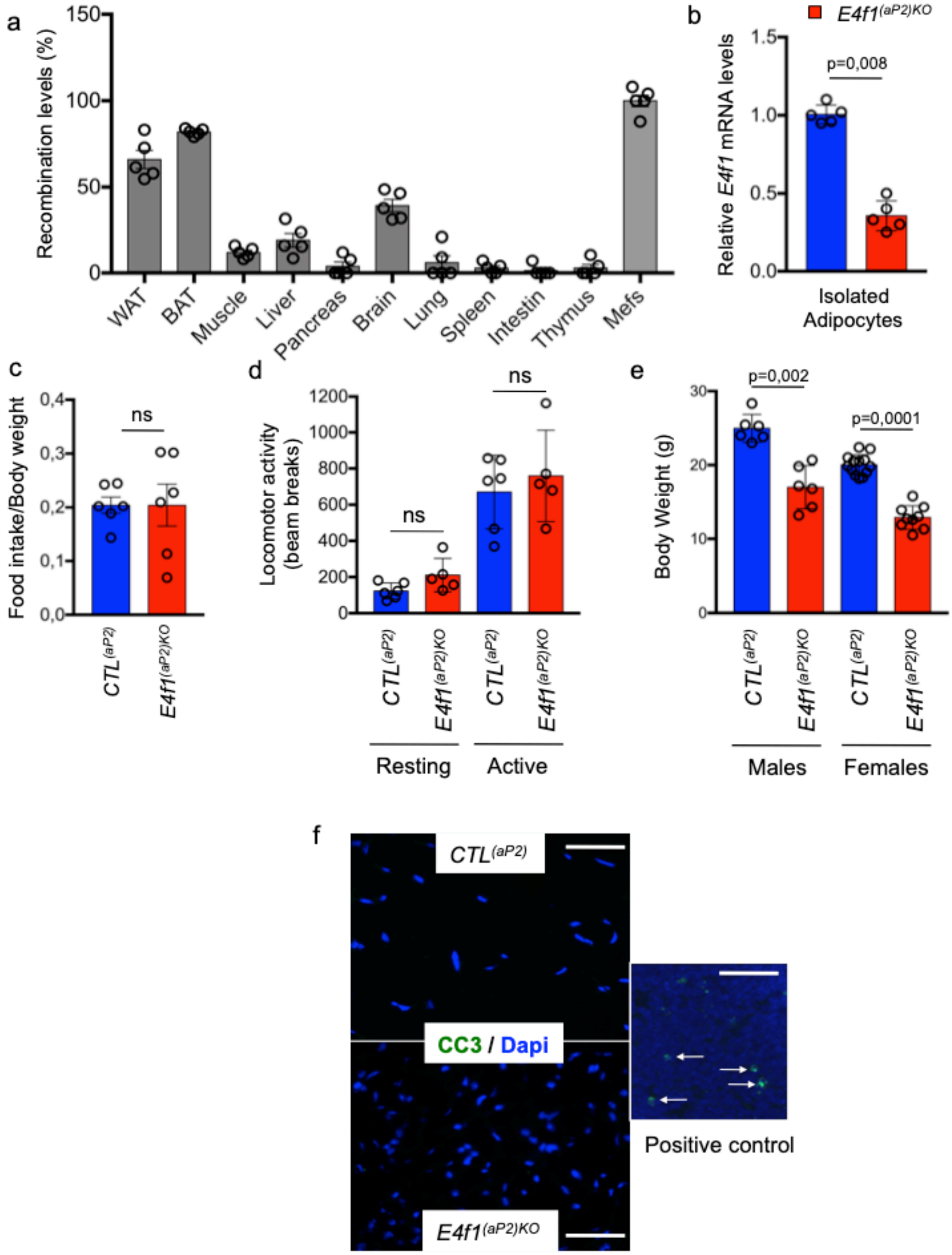

Supplementary figure 2: Characterization of *E4f1*$^{(aP2)KO}$ mice. (**a**) Efficiency of Cre-mediated recombination of the *E4f1*^{flox} allele in different organs of *E4f1*$^{(aP2)KO}$ mice, determined by qPCR on genomic DNA prepared from the indicated tissues. The values were normalized to the level of recombination evaluated in *E4f1*^{cKO} Mefs (considered as 100%) (n=5 animals/group). (**b**) RT-qPCR analysis of *E4f1* mRNA levels in isolated adipocytes prepared from epididymal white adipose tissue (WATe) of *E4f1*$^{(aP2)KO}$ and *CTL*$^{(aP2)}$ littermates (n=5 animals/group). (**c**) Food intake of 8- to 12-week-old *E4f1*$^{(aP2)KO}$ males and *CTL*$^{(aP2)}$ littermates, measured during 96 hrs (n=10 animals/group). (**d**) Spontaneous locomotor activity of *E4f1*$^{(aP2)KO}$ males and *CTL*$^{(aP2)}$ littermates, measured over 24hrs (active, 12 hrs night; resting, 12 hrs light) (n=10 animals/group). (**e**) Body weight of 8- to 12-week-old *E4f1*$^{(aP2)KO}$ and *CTL*$^{(aP2)}$ males and females under chow diet (n=7 animals/group). (**f**) IF analysis of CC3 in WATe sections prepared from 8- to 12-week-old *E4f1*$^{(aP2)KO}$ and *CTL*$^{(aP2)}$ males. A thymus tissue section prepared from wild-type mice was used as a positive control for CC3 staining. Nuclei were counterstained with DAPI. Scale bars= 500µm (left panels) and 100µm (control panel). Data were presented as mean ± standard error mean (SEM) from the indicated number of animals. Statistical analyses were performed using two-sided non-parametric Mann-Whitney U tests and the BiostaTGV software (ns, not significant). Source data are provided as a Source Data file.

Supplementary figure 3

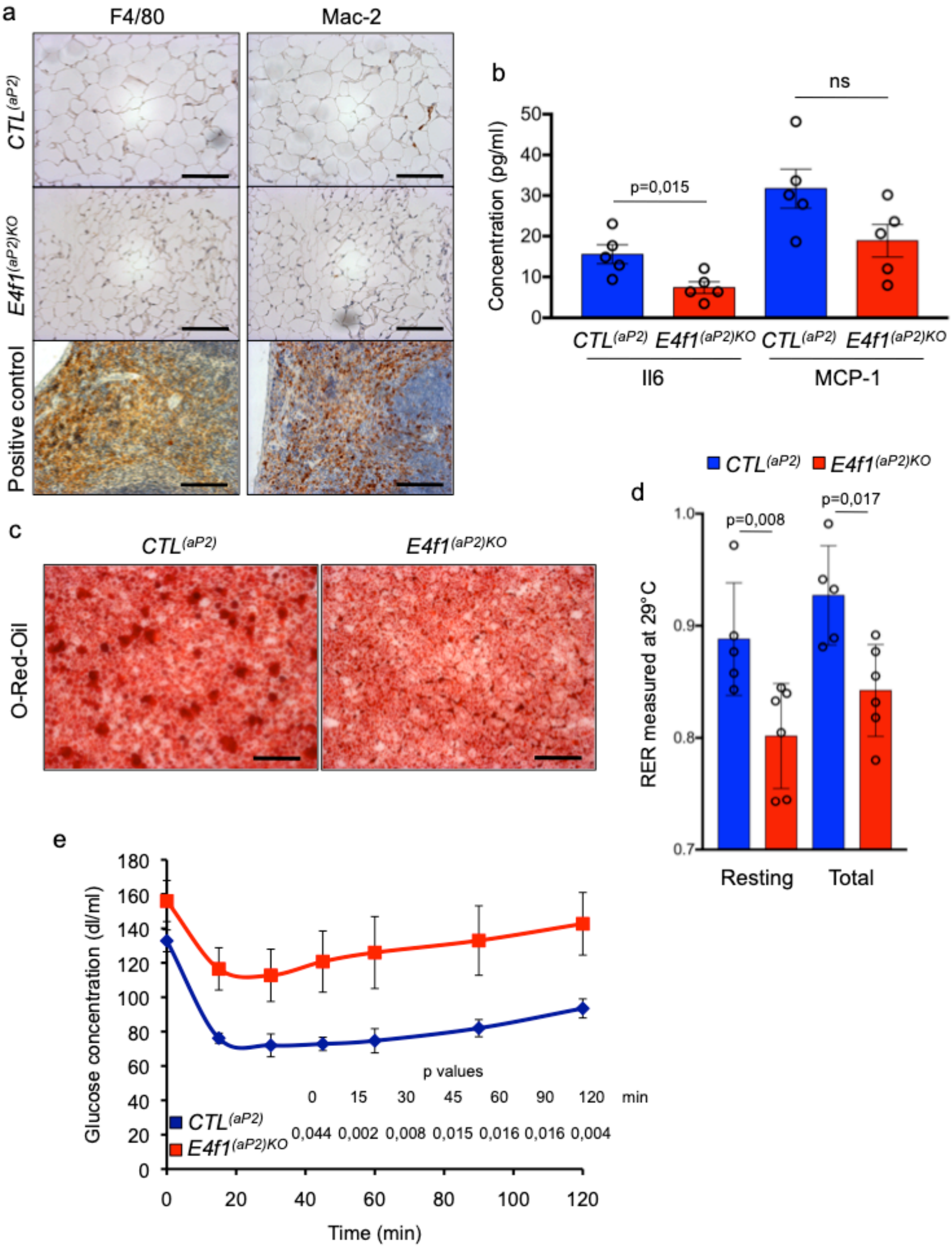

Supplementary figure 3: E4F1-deficient mice exhibit decreased adiposity. (**a**) Immunohistochemistry (IHC) analysis of the macrophage markers MAC2 and F4/80 in epididymal white adipose tissue (WATe) sections prepared from 8-to-12-week-old *E4f1*$^{(Ap2)KO}$ mice and *CTL*$^{(Ap2)}$ littermates (data are representative of independent experiments performed on n=5 animals/group). Spleen sections prepared from wild-type mice were used as positive controls. Scale bars=500µm. (**b**) Circulating levels of the pro-inflammatory cytokines Il6 and MCP-1 in the plasma of 8- to 12-week-old *E4f1*$^{(aP2)KO}$ animals and *CTL*$^{(aP2)}$ littermates (n=5 animals/group). (**c**) Representative microphotographs of Oil red O (ORO) -stained liver cryosections prepared from 8- to 12-week-old *E4f1*$^{(RERT)KO}$ mice and *CTL*$^{(RERT)}$ littermates, 13 days after Tam administration (data are representative of experiments performed on n=3 animals/group). Scale bars, 200 µm. (**d**) Respiratory exchange ratio (RER) measured at thermoneutral temperature (29°C) during the 12 hrs light period (Resting) or during 24 hrs (Total) in 8- to 12-week-old *E4f1*$^{(aP2)KO}$ and *CTL*$^{(aP2)}$ males (n=5 males/group). (**e**) Insulin tolerance test (ITT) performed on *E4f1*$^{(aP2)KO}$ mice and *CTL*$^{(aP2)}$ littermates (n=7 males/group). Data were presented as mean ± standard error mean (SEM) from the indicated number of animals. Statistical analyses were performed using two-sided non-parametric Mann-Whitney U tests and the BiostaTGV software (ns, not significant). Source data are provided as a Source Data file.

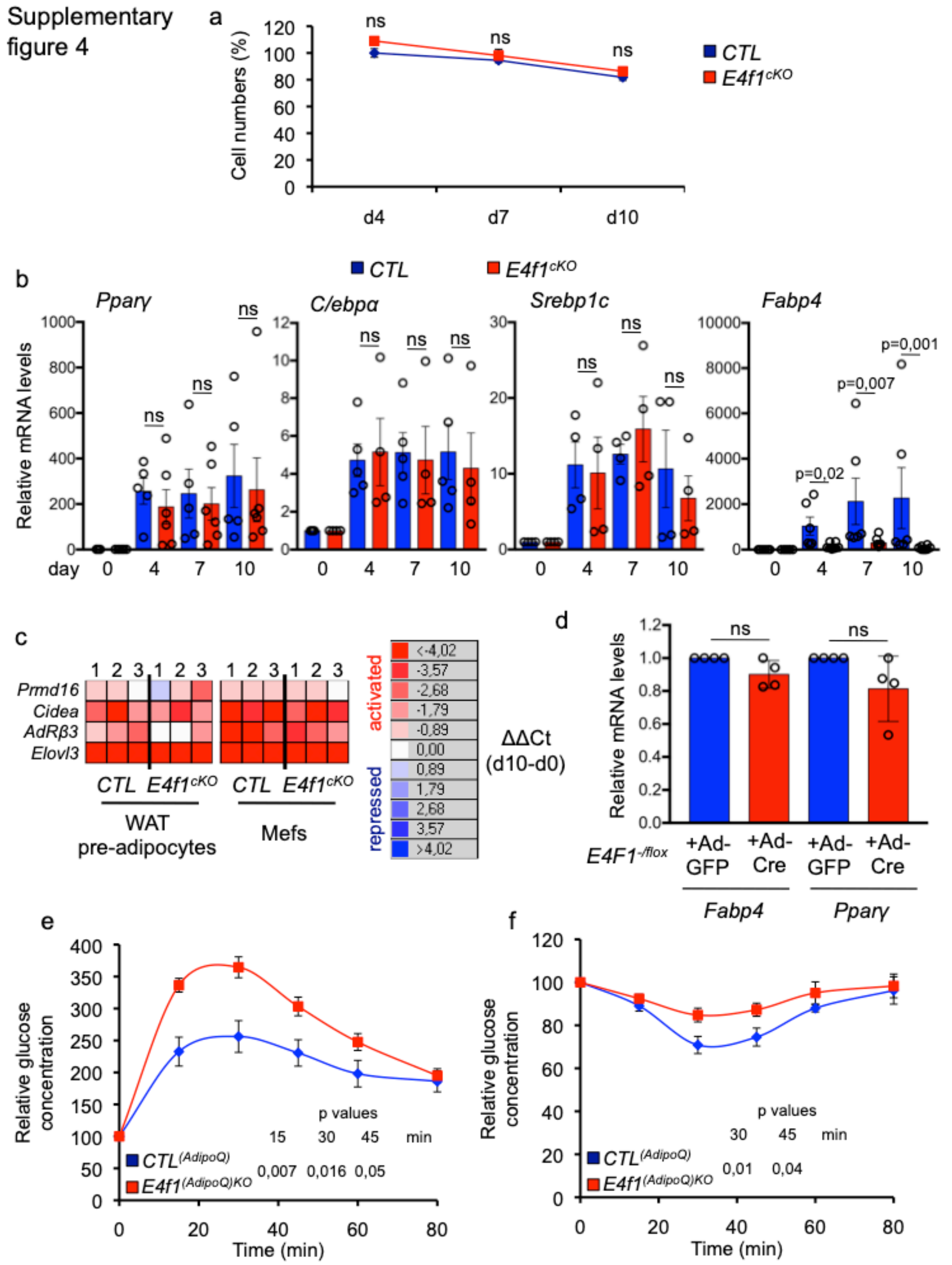

Supplementary figure 4: E4F1 deficiency impairs lipid accumulation during in vitro adipocyte differentiation. (**a**) Cell numbers of *E4f1*^{cKO} or *CTL* Mefs during in vitro adipocyte differentiation (represented as the percentage of the initial number of cells counted at day 4 after the addition of the differentiation cocktail, n=4 independent populations of cells/group). (**b**) RT-qPCR analysis of *Pparg*, *C/ebpa*, *Srebp1c* and *Fabp4/aP2* mRNA levels in *E4f1*^{cKO} or CTL Mefs at the indicated time points after induction of adipocyte differentiation (n=4 independent populations of cells/group). (**c**) RT-qPCR analysis of the brown adipocyte markers *Prdm16*, *Cidea*, *Adrb3*, and *Elovl3* mRNA levels in *E4f1*^{cKO} adipocytes and their matched *CTL* cells prepared either from primary white pre-adipocytes or Mefs, as indicated. The heat map represents the relative mRNA levels of the indicated genes determined by a microfluidic RT-qPCR approach, calculated using the ΔΔCt method, between day 0 and day 10 after induction of adipocyte differentiation. Each column represents an independent experiment performed on paired samples. Raw data were normalized by 3 independent housekeeping genes (18S, β2microglobulin and tubulin β5). For experiments described in (a) to (c), cells were transduced with a Cre-encoding retrovirus 5 days prior to the induction of adipocyte differentiation. (**d**) RT-qPCR analysis of *Fabp4* and *Ppary* mRNA levels in *E4f1*$^{-/flox}$ differentiated Mefs transduced with GFP- (Ad-GFP) or Cre- (Ad-Cre) encoding adenoviruses 7 days after induction of adipocyte differentiation (n=4 independent populations of cells/group). (**e-f**) Glucose tolerance test (IPGTT) (**e**) and insulin tolerance test (ITT) (**f**) performed on 8- to 12-week-old *E4f1*$^{(AdipoQ)KO}$ mice and *CTL*$^{(AdipoQ)}$ littermates (n=5 males/group). Data were presented as mean ± standard error mean (SEM) from the indicated number of animals. Statistical analyses were performed using two-sided non-parametric Mann-Whitney U tests and the BiostaTGV software (ns, not significant). Source data are provided as a Source Data file.

Supplementary figure 5

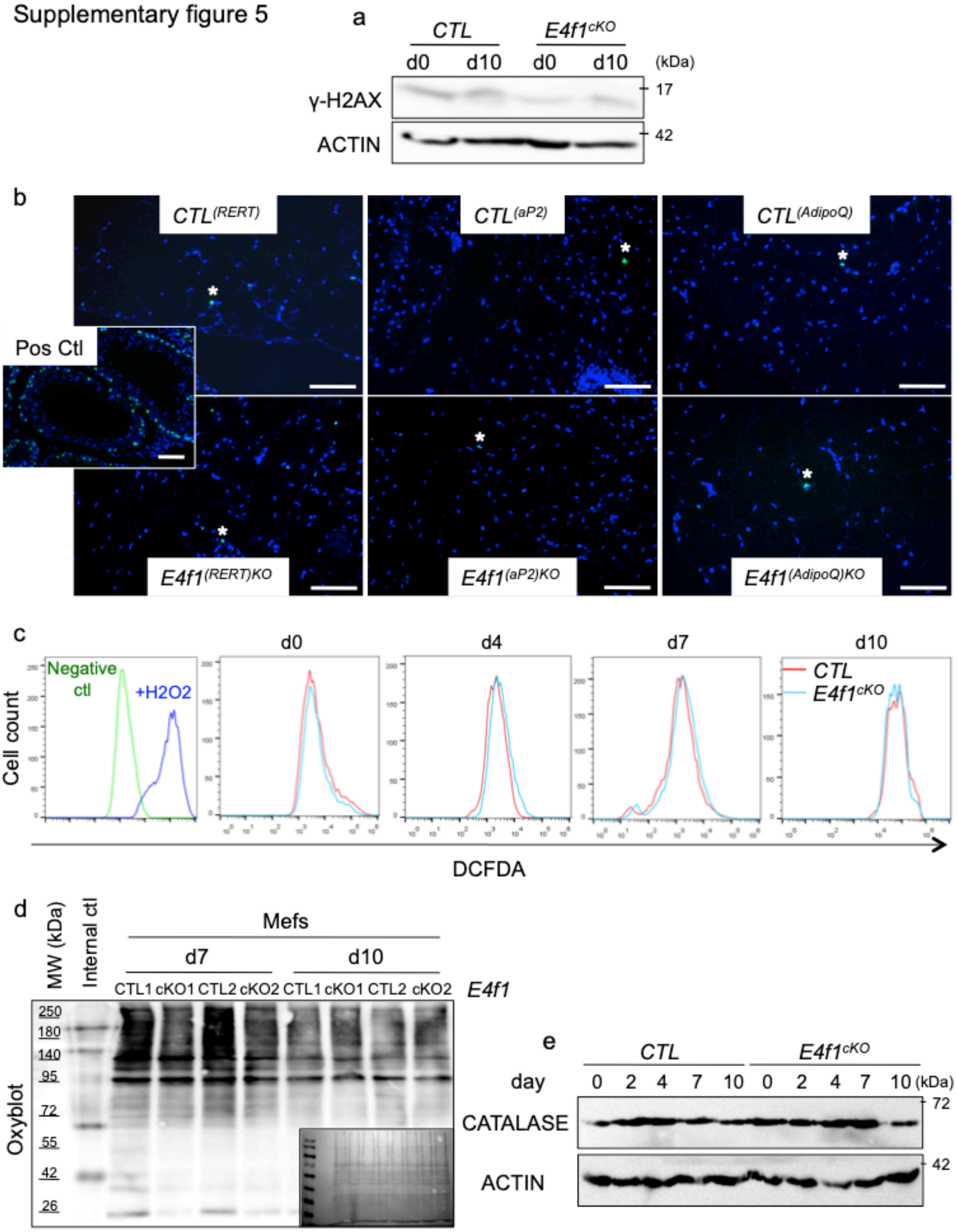

Supplementary figure 5: E4F1-deficient adipocytes does not result from increased DNA damage or oxidative stress. (**a**) Immunoblot analysis of γ-H2AX and ACTIN (loading control) protein levels in *E4f1*^{cKO} and *CTL* Mefs, 10 days after in vitro adipocyte differentiation (representative data of experiments performed on n=3 independent populations of cells/group). (**b**) IF analysis of γ-H2AX expression on epididymal white adipose tissue (WATe) sections prepared from *E4f1*$^{(RERT)KO}$, *E4f1*$^{(aP2)KO}$ or *E4f1*$^{(AdipoQ)KO}$ mice and their respective *CTL* littermates (representative data of experiments performed on n=3 males/group). Stars indicate γ-H2AX positive cells. A testis tissue section prepared from wild-type mice was used as a positive control for γ-H2AX staining. Scale bars= 500μm (right panels) and 100μm (positive control). (**c**) Reactive oxygen species (ROS) levels in *E4f1*^{cKO} and CTL Mefs were determined at the indicated time points during adipocyte differentiation by flow cytometry using the ROS-sensitive DCFDA probe. Mefs treated with H2O2 (1mM) for 20min were used as a positive control for DCFDA staining (Data are representative of n=5 independent experiments). (**d**) Immunoblots showing no significant difference in the total amount of carbonylated-proteins (Oxyblot) between *E4f1*^{cKO} and CTL Mefs, 7 or 10 days after adipocyte differentiation. The inset shows the same membrane after red ponceau staining to ensure equal loading. (**e**) Immunoblot analysis of CATALASE and ACTIN (loading control) protein levels in *E4f1*^{cKO} and CTL Mefs during adipocyte differentiation. Molecular weight (MW) is indicated in kDa.

Supplementary figure 6

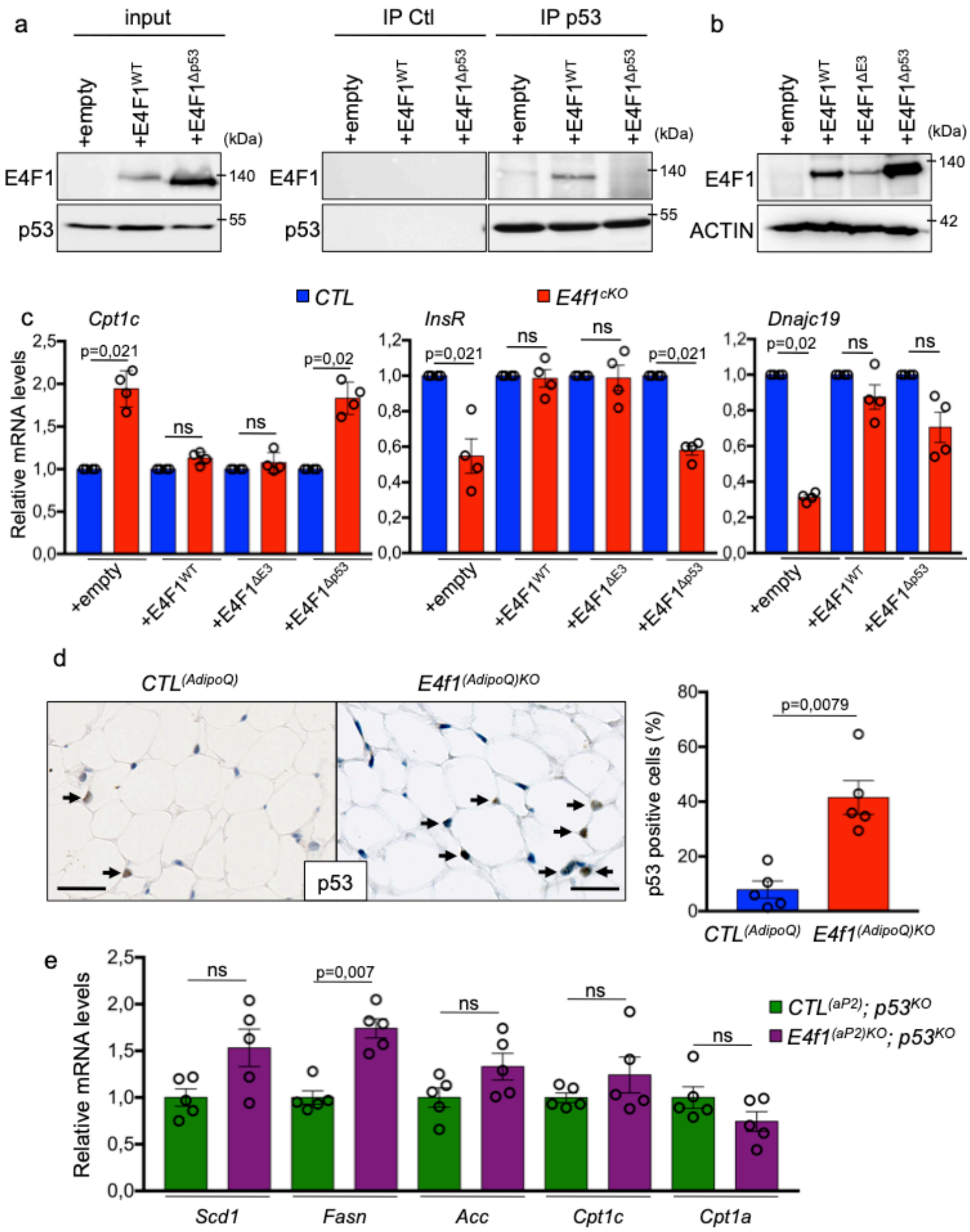

Supplementary figure 6: Lipid metabolism defects in E4F1-deficient adipocytes are p53-dependent. (**a**) Co-immunoprecipitation experiments showing physical interaction of E4F1^{WT}, but not the E4F1$^{\Delta p53}$ mutant lacking the p53 interaction domain, with endogenous p53 protein in primary Mefs. To avoid interference with endogenous E4F1 protein, ectopic E4F1 proteins were expressed upon viral transduction of *E4f1^{cKO}* cells with retroviruses encoding full length E4F1^{WT} or E4F1$^{\Delta p53}$. The antibodies used for immunoprecipitation and immunoblotting are indicated. Data are representative of 3 independent experiments. (**b**) Immunoblot analysis of ectopic E4F1^{WT}, E4F1$^{\Delta E3}$ and E4F1$^{\Delta p53}$ and ACTIN (loading control) protein levels in total protein extracts prepared from *E4f1^{cKO}* Mefs transduced with retroviruses encoding E4F1^{WT} or mutants before induction of adipocyte differentiation (data are representative of 3 experiments on independent populations of cells). (**c**) RT-qPCR analysis of *Cpt1c*, *InsR* and of the E4F1-direct target gene *Dnajc19* mRNA levels in CTL or *E4f1*^{cKO} Mefs transduced with retroviruses encoding ectopic E4F1^{WT}, E4F1$^{\Delta E3}$ or E4F1$^{\Delta p53}$ proteins, as indicated (n=5 independent populations of cells/group). (**d**) Representative immunohistochemistry (IHC) analysis of p53 protein levels in WATe tissue sections prepared from *E4f1$^{(AdipoQ)KO}$* adult mice and *CTL$^{(AdipoQ)}$* littermates, 12- weeks after Tamoxifen (Tam) administration. Arrows indicate p53-positive cells. Scale bars= 200µm. Histobars represent the percentages of p53 positive cells in each experimental group (n=5 animals/group). (**e**) Relative mRNA levels of *Scd1*, *Fasn*, *Acc*, *Cpt1c* and *Cpt1a* in WATe of 8- to 12-week-old *E4f1$^{(aP2)KO}$; p53^{KO}* mice and control *p53^{KO}* animals determined by a microfluidic RT-qPCR approach. Raw data were normalized by the β2m housekeeping gene. Histobars represent the relative mRNA levels of the indicated genes shown as the ratio between *E4F1$^{(aP2)KO}$; p53^{KO}* and *E4F1$^{(aP2)WT}$; p53^{KO}* animals (n=5 males/group). Molecular weight is indicated in kDa. Data were presented as mean ± standard error mean (SEM) from the indicated number of independent samples. Statistical analyses were performed using two-sided non-parametric Mann-Whitney U tests and the BiostaTGV software (ns, not significant). Source data are provided as a Source Data file.

Supplementary figure 7

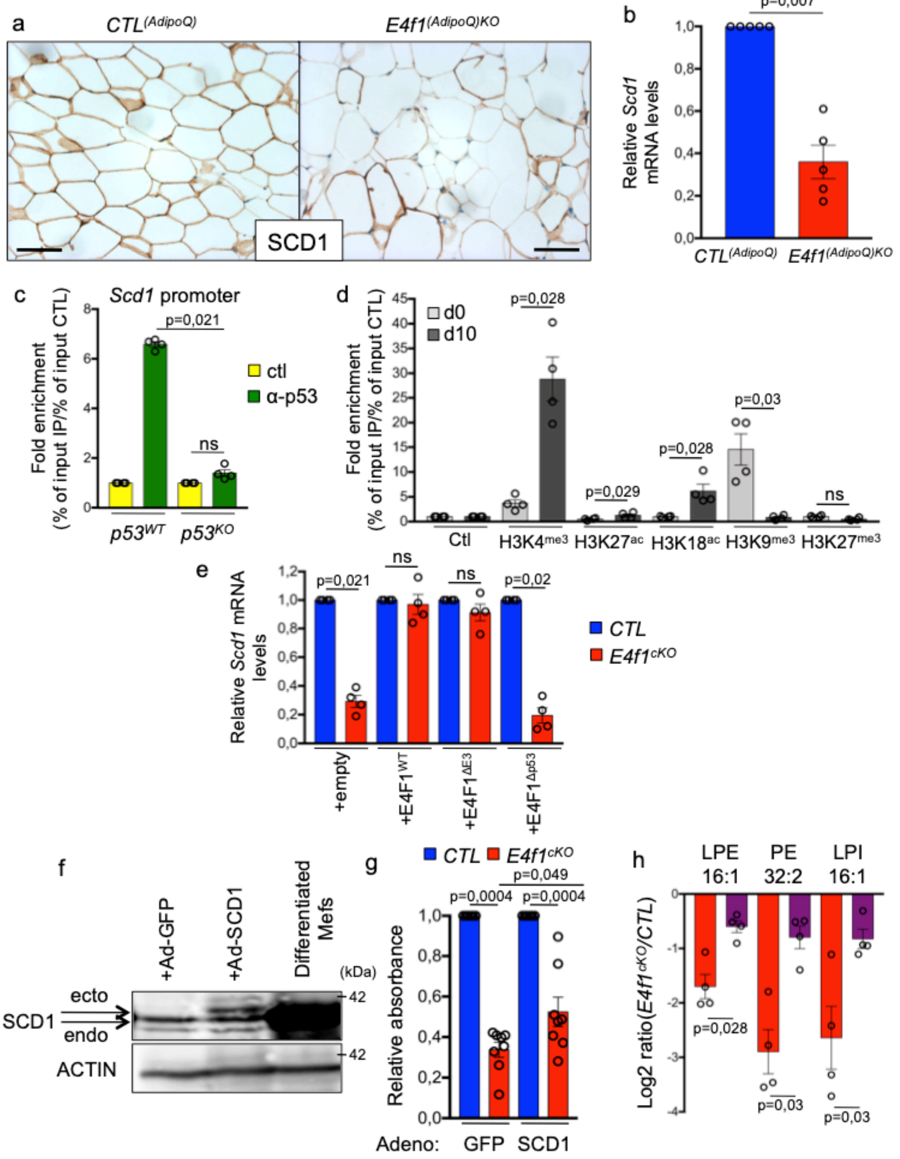

Supplementary figure 7: E4F1 deficiency impacts on *Scd1* expression (**a**) Immunohistochemistry (IHC) analysis of SCD1 protein levels in epididymal white adipose tissue (WATe) prepared from $E4f1^{(AdipoQ)KO}$ and $CTL^{(AdipoQ)}$ littermates, 12 weeks after Tamoxifen (Tam) administration (n=3 males/group). Scale bars= 200µm (**b**) RT-qPCR analysis of *Scd1* mRNA levels in WATe of $E4f1^{(AdipoQ)KO}$ mice and $CTL^{(AdipoQ)}$ littermates, 12 weeks after Tam administration (n=5 males/ group). (**c**) p53 qChIP on *Scd1* promoter in $p53^{KO}$ or $p53^{WT}$ control Mefs. Histobars represent the relative ratio between the mean value of immunoprecipitated chromatin (calculated as a percentage of the input) with the p53 antibody and the one obtained with a control antibody (n=4 independent experiments). (**d**) qChIP analysis of epigenetic marks at the p53 binding site of the *Scd1* promoter during adipocyte differentiation. Tri-methylation on lysine 9 (H3K9me3), lysine 4 (H3K4me3) and lysine 27 (H3K27me3), or acetylation on lysine 18 (H3K18ac) and lysine 27 (H3K27ac) of histone H3 were determined at the indicated time points during adipocyte differentiation (n=4 independent experiments). (**e**) RT-qPCR analysis of *Scd1* mRNA levels in $E4f1^{cKO}$ or CTL Mefs transduced with retroviruses encoding wild type E4F1 (E4F1WT), the E4F1DE3 or the E4F1Dp53 mutants (n=4 independent populations of cells/group). (**f**) Representative immunoblot analysis of SCD1 and ACTIN (loading control) protein levels in undifferentiated Mefs transduced with adenoviruses encoding GFP or SCD1. Ectopic SCD1 protein levels were compared to those of endogenous SCD1 in differentiated adipocytes (n=3 independent experiments). (**g**) Quantification of TG accumulation in $E4f1^{cKO}$ or CTL Mefs transduced with GFP- or SCD1- encoding adenoviruses, determined by Oil Red O (ORO) staining (n=6 populations of cells/group). (**h**) ESI-MS-MS phospholipid (PL) profiling of $E4f1^{cKO}$ and CTL Mefs, in p53 WT or KO backgrounds, 10 days after induction of adipocyte differentiation. Histobars represent the relative levels of C16:1 lysophospatidylethanolamine (LPE), C32:2 phosphatidylethanolamine (PE), and C16:1 lysophosphatidylinositol (LPI), shown as the ratio (expressed as log2) of the percentage of the total PL between $E4f1^{cKO}$ and CTL Mefs (n=3 independent populations of Mefs/group). Molecular weight is indicated in kDa. Data were presented as mean ± standard error mean (SEM) from the indicated number of independent samples. Statistical analyses were performed using two-sided non-parametric Mann-Whitney U tests and the BiostaTGV software (ns, not significant). Source data are provided as a Source Data file.

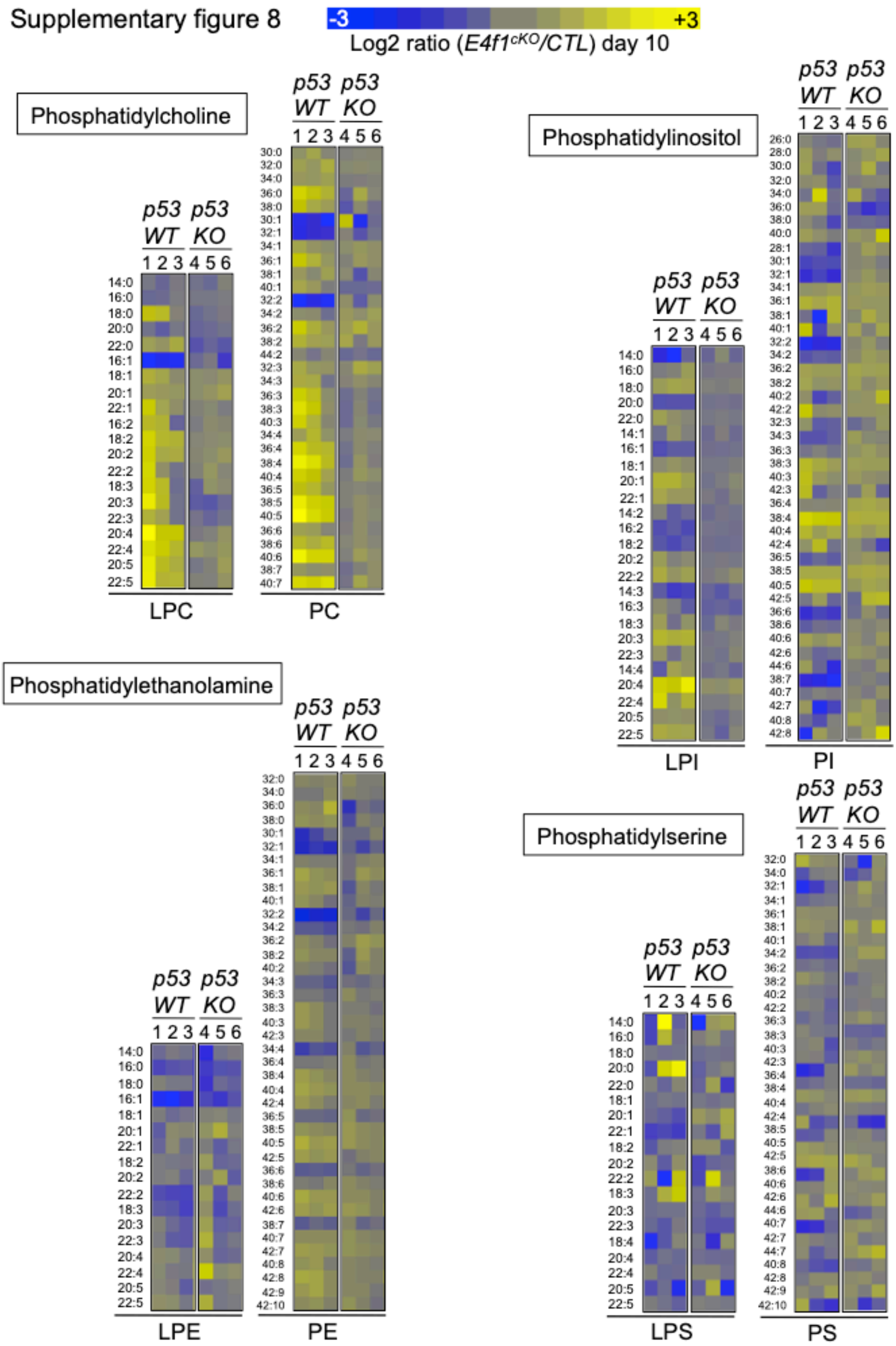

Supplementary figure 8: E4F1 deficiency impairs MUFAs production. Phospholipid (PL) profiling was performed by ESI-MS-MS on three independent pairs of matched samples prepared from *E4f1*^{cKO} and CTL Mefs, and their *p53*^{KO} counterparts, 10 days after induction of adipocyte differentiation in vitro. The heat maps show the ratio (expressed as log2 of the percentage of total PL) for the indicated PL species between *E4f1*^{cKO} and CTL cells, including lysophosphatidylcholine (LPC), phosphatidylcholine (PC), lysophosphatidylinositol (LPI), phosphatidylinositol (PI), lysophosphatidylethanolamine (LPE), phosphatidylethanolamine (PE), lysophosphatidylserine (LPS) and phosphatidylserine (PS). The genotype of the cells according to the *p53* locus is indicated.

qPCR primer

LIPID METABOLISM

Fatty acid oxidation			
Cpt1B	mouse	CGA GGA TTC TCT GGA ACT GC	GGT CGC TTC TTC AAG GTC TG
Cpt1A	mouse	TCCACCCTGAGGCATCTATT	ATGACCTCCTGGCATTCTCC
Cpt1C	mouse	TCTTCACTGAGTTCCGATGGG	ACGCCAGAGATGCCTTTTCC
Acsl3	mouse	CAATTACAGAAGTGTGGGACT	CACCTTCCTCCCAGTTCTTT
Acsl4	mouse	TGCCAAAAGTGACCAGTCCTATG	TGTTACCAAACCAGTCTCGGGG
Acsl5	mouse	TCCCAGCCCACCTCTGATGATGTG	ACAAACTGTCCCGCCGAATG
Cte1 (acot1)	mouse	GCAGCCACCCCGAGGTAAA	GCCACGGAGCCATTGATG
Acox	mouse	GGTGGACCTCTGTCTTGTTCA	AAACCTTCAGGCCCAAGTGAG
Fads3	mouse	GTG ATC CAC ACG AAC CAG TG	TCC CGC TTT TTC TTG TCC TA
GyK	mouse	CCGCGAAGAAAGCAGTTCTG	CAAAAAACGTGTCGAGCTGGTA
Mcad	mouse	ACTTCGTGGCTTCGTCTAGA	GAGCAGGTTTCAAGATCGCA
Cidea	mouse	ATCACAACTGGCCTGGTTACG	TACTACCCGGTGTCCATTTCT
Lipin1	mouse	GTCTGCAGTGCTCCCCTTTC	ATCCACGGACTCCCCATT
Gamt	mouse	GGCAGCCACATAAGGTTGTT	CGTGAGGTTGCAGTAGGTGA
Crot	mouse	GAACGGACATTTCAGTACCAGG	CTTCATTTGCGAATGGTTTCACT
Dhrs3	mouse	CAGAACGGCCATATTGTGTGC	CGAAGGCTGACGCTTTTGA
Pltp	mouse	CGCAAAGGGCCACTTTTACTA	GCCCCCATCATATAAGAACCAG
Lipid transport			
Lpl	mouse	GCTGGAAAGTGCCTCCATTG	CTGCTGGCGTAGCAGGAAGT
Vldl-r	mouse	TGACGCAGACTGTTCAGACC	GCCGTGGATACAGCTACCAT
Fatp1 (Slc27a1)	mouse	TCTGTTCTGATTCGTGTTCGG	CAGCATATACCACTACTGGCG
Lipid synthesis			
Ap2 (fatty acid binding protein 4)	mouse	CAGAAGTGGGATGGAAAGTCG	CGACTGACTATTGTAGTGTTTGA
Adipor1	mouse	GCATCTCTGCCATCATTGTG	TGGACACACCATAGAAGTGGAC
Srebp1c	mouse	CACTTCTGGAGACATCGCAAAC	TGGTAGACAACAGCCGCATC
Fatty acid Syntase	mouse	TGCTCCCAGCTGCAGGC	GCCCGGTAGCTCTGGGTGTA
Stearoyl CoA desaturase 1	mouse	AAAGAGAAGGGCGGAAAACT	GCGTTGAGCACCAGAGTGTA
Agpat1	mouse	CACCCAGGATGTGAGAGTCTG	CTGACAACGTCCAGGCGAGG
Agpat2	mouse	GCAACGACAATGGGGACCTG	ACAGCATCCAGCACTTGTACC
Gpat1	mouse	CTCGTGTGGGTGATTGTGAC	AGCAAGTCCTGCGCTATCAT
Gpat4 (Agpat6)	mouse	AGCTTGATTGTCAACCTCCTG	CCGTTGGTGTAGGGCTTGT
Cav1	mouse	ATGTCTGGGGGCAAATACGTG	CGCGTCATACACTTGCTTCT
Elovl3	mouse	TCC GCG TTC TCA TGT AGG TCT	GGA CCT GAT GCA ACC CTA TGA
Elovl6	mouse	TGC TGC ATC CAG TTG AAG AC	TGC CAT GTT CAT CAC CTT GT
ATP citrate lyase (ACLY)	mouse	ACCCTTTCACTGGGGATCACA	GACAGGGATCAGGATTTCCTTG
Acc	mouse	CCCAGCAGAATAAAGCTACTTTGG	TCCTTTTGTGCAACTAGGAACGT
GLYCOLYSIS/NEOGLUCO			
Glut1	mouse	GGCCGCCTCATGTTGGCTGT	TGGGCTCTCCGTAGCGGTGG
Glut4	mouse	GTGACTGGAACACTGGTCCTA	CCAGCCACGTTGCATTGTAG
Insulin Receptor	mouse	TTTGTCATGGATGGAGGCTA	CCTCATCTTGGGGTTGAACT
G6Pase	mouse	CTGAGCGCGGGCATCATAAT	GATTCTTAGGATCGCCCAGAAAG
Fructose1,6P	mouse	CACCGCGATCAAAGCCATCT	AGGTAGCGTAGGACGACTTCA
Phosphofructokinase	mouse	GAAACATGAGGCGTTCTGTGT	CCCGGCACATTGTTGGAGA
Tigar	mouse	ATGGCGGTGAAGTACGACTC	GGGGAGGATTAGCTGACAAA
Hexokinase II	mouse	TGATCGCCTGCTTATTCACGG	AACCGCCTAGAAATCTCCAGA
Pgm-M	mouse	GAACCGCTTCAGCGGCTGGT	AGCCAGCATCTCGCAACGCC
Pgm-B	mouse	TGCCCGTCGCCATGGCTGCC	CGGTGGGACATCATAAGATC
GLUTAMINOLYSIS			
Glutamate DH	mouse	CCCAACTTCTTCAAGATGGTGG	AGAGGCTCAACACATGGTTGC
Gls2	mouse	GGAGCGTATCCCTATCCACA	CTTCTTTCGGAATGCCTGAG

MITOCHONDRIA BIOGENESIS / OXPHOS			
Atp5b	mouse	GTCCCGGGCTATTGCTGAGTTG	TCCCCATGTGACCCGTGAAGA
Sco2	mouse	CAGCCTGTCTTCATCACTGTGGA	GACACTGTGGAAGGCAGCTATGTGCC
Tfam	mouse	GCTCTACACGCCCCTGGTTTCTGG	TCGCTGTAGTGCCTGCTGCTCCTG
Ndufs8	mouse	TGGCGGCAACGTACAAGTAT	CCTCGGATGAGTTCTGTCCA
ATP synthase	mouse	GCCCGGGTAGCTCTGACTGGTTTG	GTCGGTGGCTAGGGTGGGCTGGTA
Pgc1a	mouse	CCCTGCCATTGTTAAGACC	TGCTGCTGTTCCTGTTTTC
Pgc1b	mouse	GGACGCCAGTGACTTTGACT	TTCATCCAGTTCTGGGAAGG
Sdha	mouse	GGAACACTCCAAAAACAGACCT	CCACCACTGGGTATTGAGTAGAA
Dnajc19	mouse	GTAGCAGTCGGGTTGACCAT	TTGGCAGTAGGGCTTACACC
DIFFERENTIATION			
Pparg	mouse	GCAAGACATAGACAAAACACCAGTGTGA	AGCAACCATTGGGTCAGCTCTTGTGA
CAAT-enhancer binding protein alpha	mouse	GAACAGCAACGAGTACCGGGTA	GCCATGGCCTTGACCAAGGAG
Prdm16	mouse	CAGCACGGTGAAGCCATTC	GCGTGCATCCGCTTGTG
AdRβ3	mouse	AGCAGACAGGGACAGAGGGGTTGCCT	TGGAGGGTGGAGAGGGGCGTCCT
TRANSCRIPTION FACTOR			
p53	mouse	GTGTTTCATTAGTTCCCCACCTTGAC	ATGGGAGGCTGCCAGTCCTAACCC
E4f1	mouse	CTGGGCATTCTTGGTTTTGT	CCAAAGCCTACCTGCTCAAG
E4F1	human	GAAAGAGCTTCCGGGAGTCG	CCACGTCCTTGCTCACACTG
CELL DEATH			
Bax	mouse	GGA GCA GCT TGG GAG CG	AAA AGGCCCCTG TCT TCATGA
Noxa	mouse	GAAGTCGCAAAAGAGCAGGATGAG	TGCCGTAAATTCACTTTGTCTCCA
Puma	mouse	GCGGCGGAGACAAGAAGA	AGTCCCATGAAGAGATTGTACATGAC
Fas	mouse	AAACAAACTGCACCCTGACC	CAACCATAGGCGATTTCTGG
Lrdd	mouse	TCGCTGTCGTGAGGTAGTTG	GAGAAGTGCTCCCTCTGGTG
Unc5b	mouse	CGACACCGAGCCTAGCAG	CCAATAGGAAGTACGGCAGC
CELL PROLIFERATION			
p21	mouse	TCTATCACTCCAAGCGCAGA	CACACAGAGTGAGGGCTAAGGC
Trp53inp1	mouse	CTACCTCAGCACCCGCAG	GCCCAATATCACAGACGAGA
Aurka	mouse	TCTAGAATATGCGCCCCTTG	AGCGTTTGCCAACTCAGTG
Gadd45a	mouse	AGACCGAAAGGATGGACACG	TGACTCCGAGCCTTGCTGA
Espl1	mouse	CTAGCGGCCGTCTATCAGTT	CGTGGTCCTGCAGTCTCTTAG
SENESCENCE			
PAI1	mouse	CTCCGAGAATCCCACACAG	ACTTTGAATCCCATAGCATC
PML	mouse	AGCAGGAGGCTTCTCAGACAGT	CTTGATGATCTTCCTGGAGCAA
ME1 v1	mouse	TCAACAAGGACTTGGCTTTTACT	TGCAGGTCCATTAACAGGAGAT
18S	mouse/human	GTAACCCGTTGAACCCCATT	CCATCCAATCGGTAGTAGCG
Beta2m	mouse	TTCTGGTGCTTGTCTCACTG	TATGTTCGGCTTCCCATTCT
Tubulin beta 5	mouse		
qChIP Primers			
SCD1	mouse	GCTATCTCTGCGCTCTTTAC	CCGGGCTGAACACCCATCC

Figure 2d

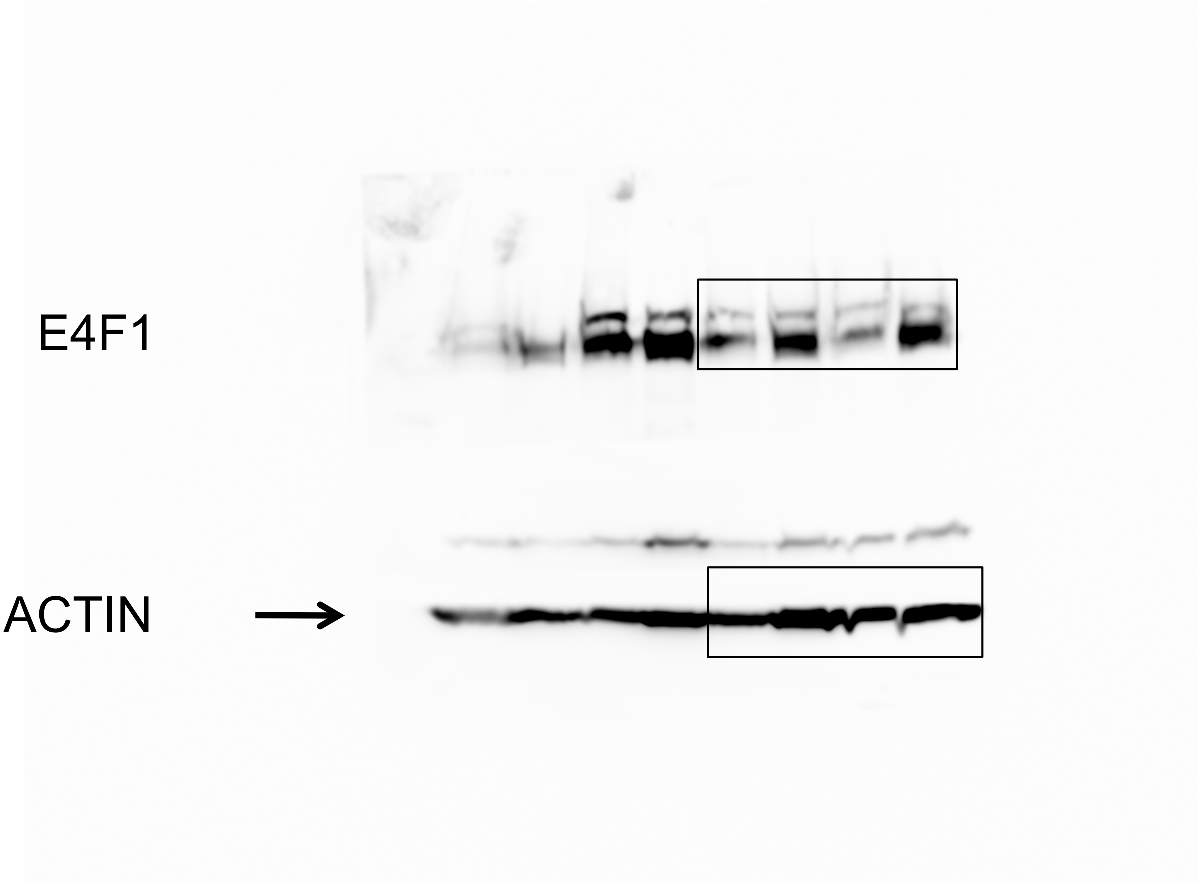

Figure 2f

E4F1

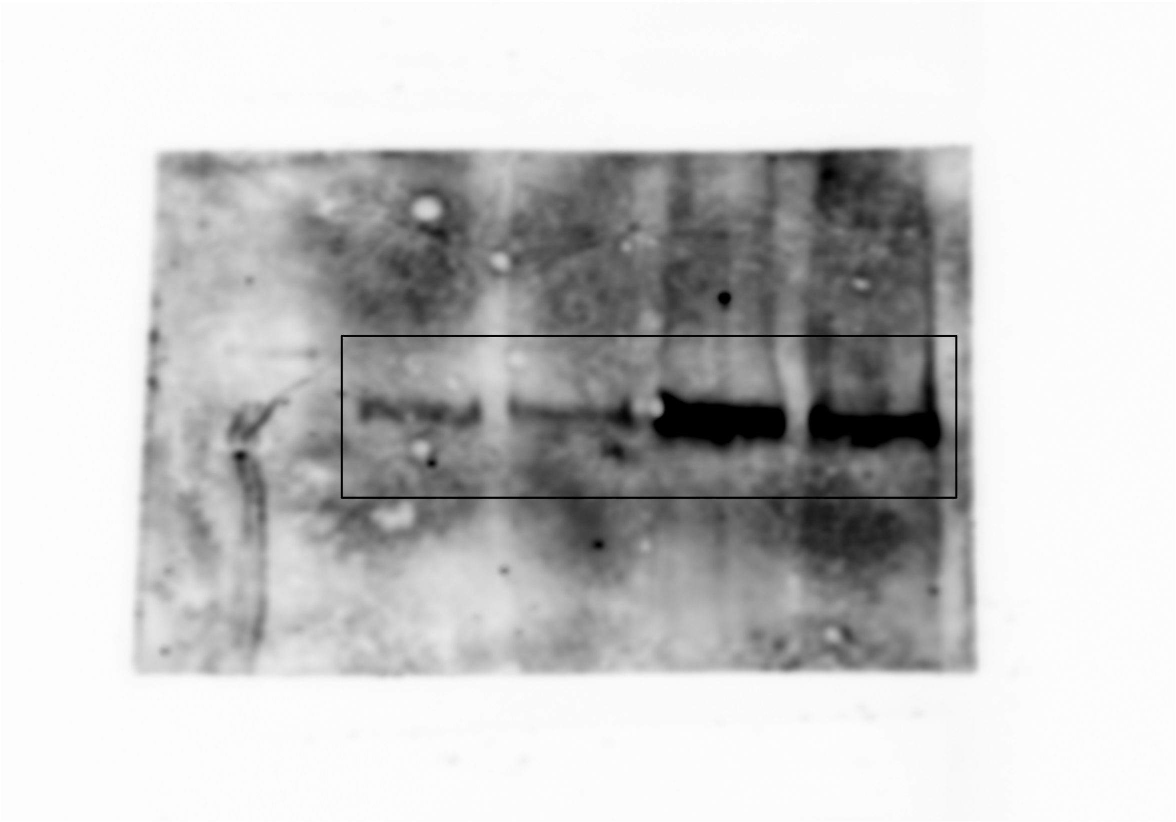

ACTIN

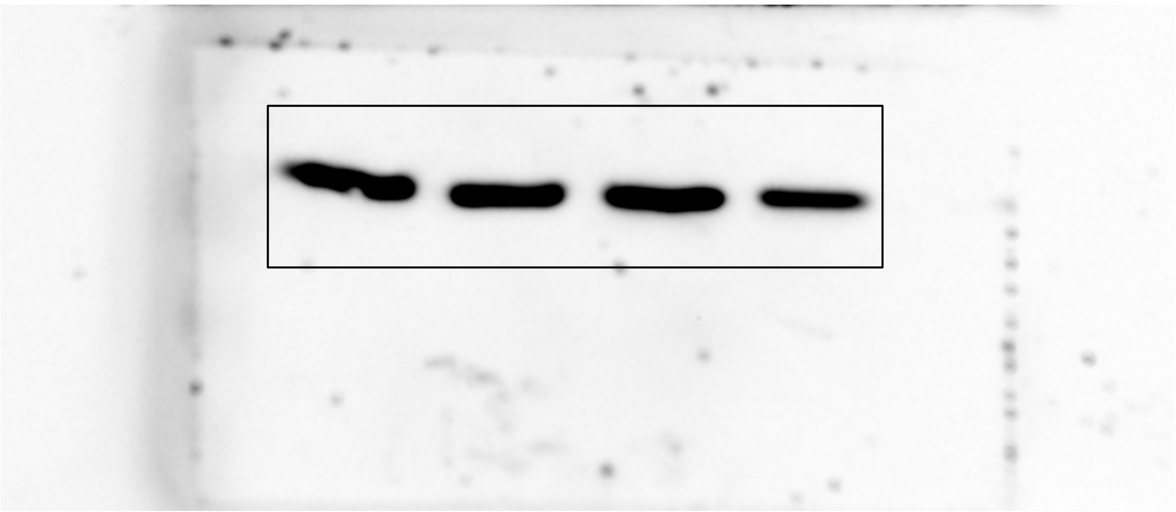

Figure 2h

E4F1

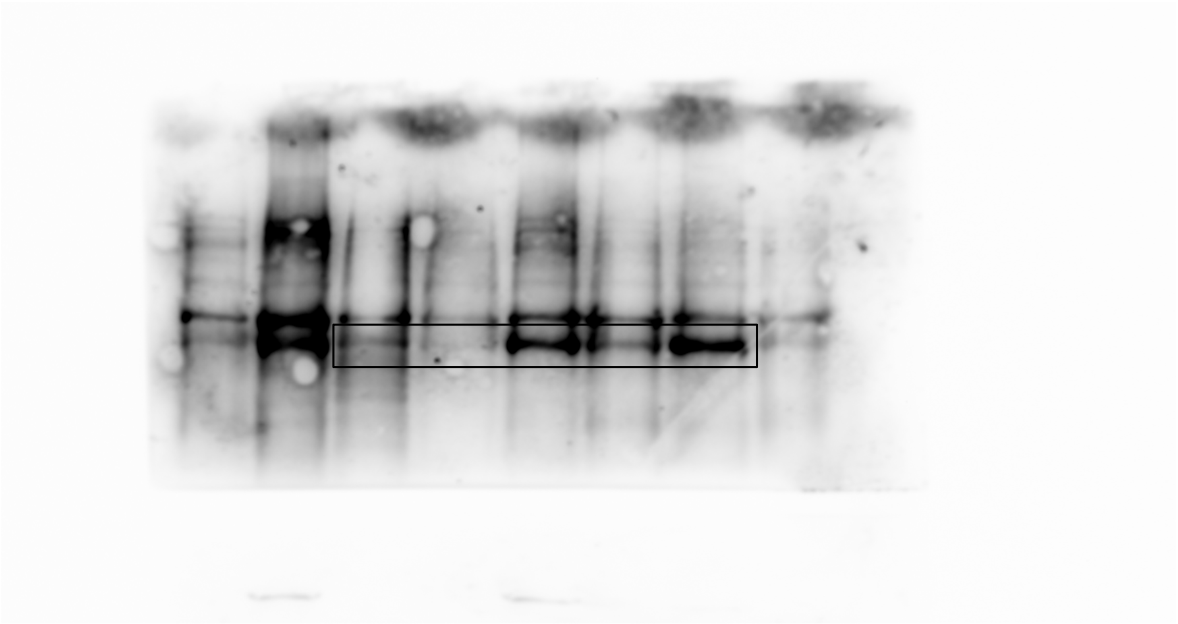

Figure 3d

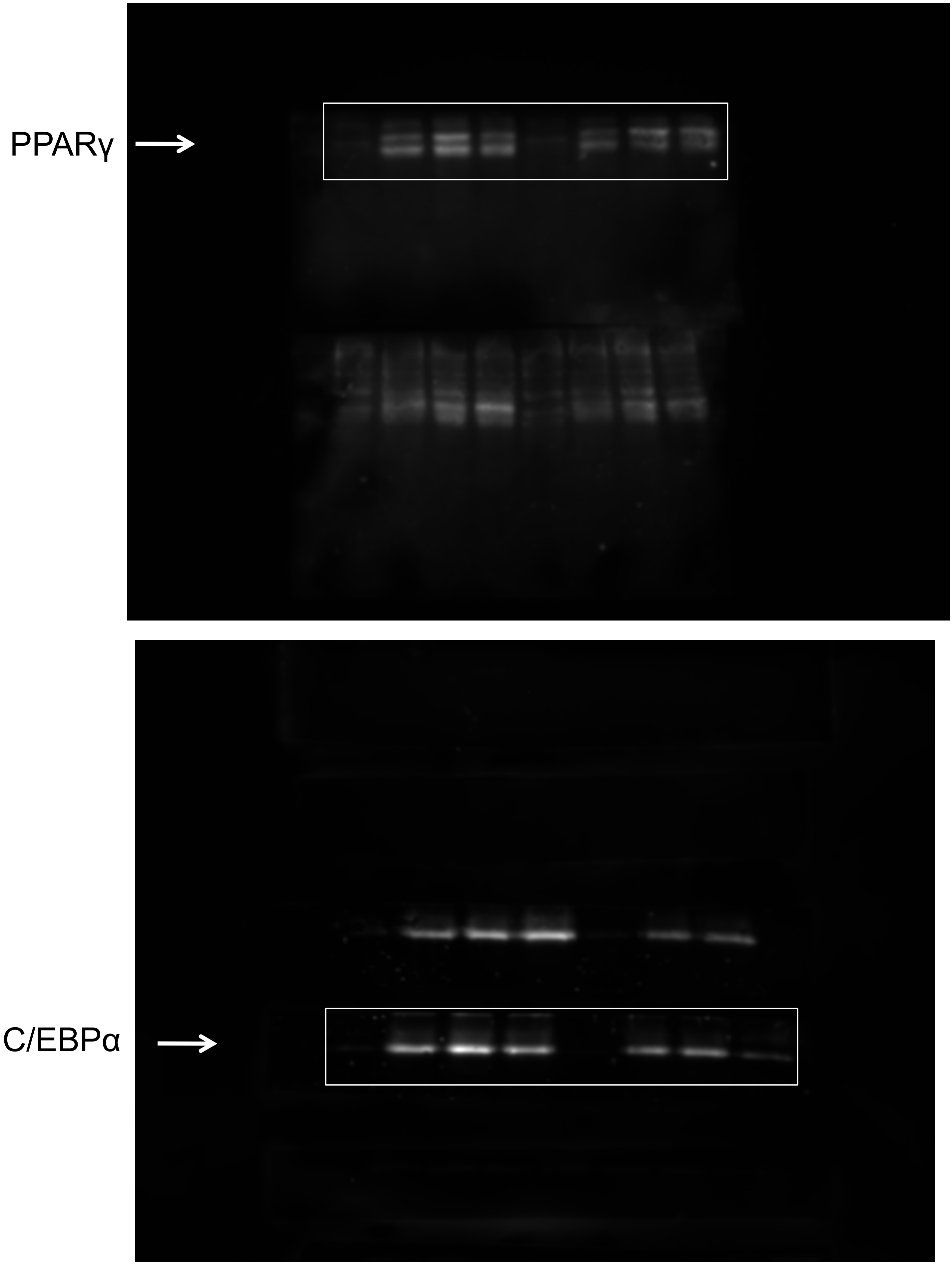

Figure 3d

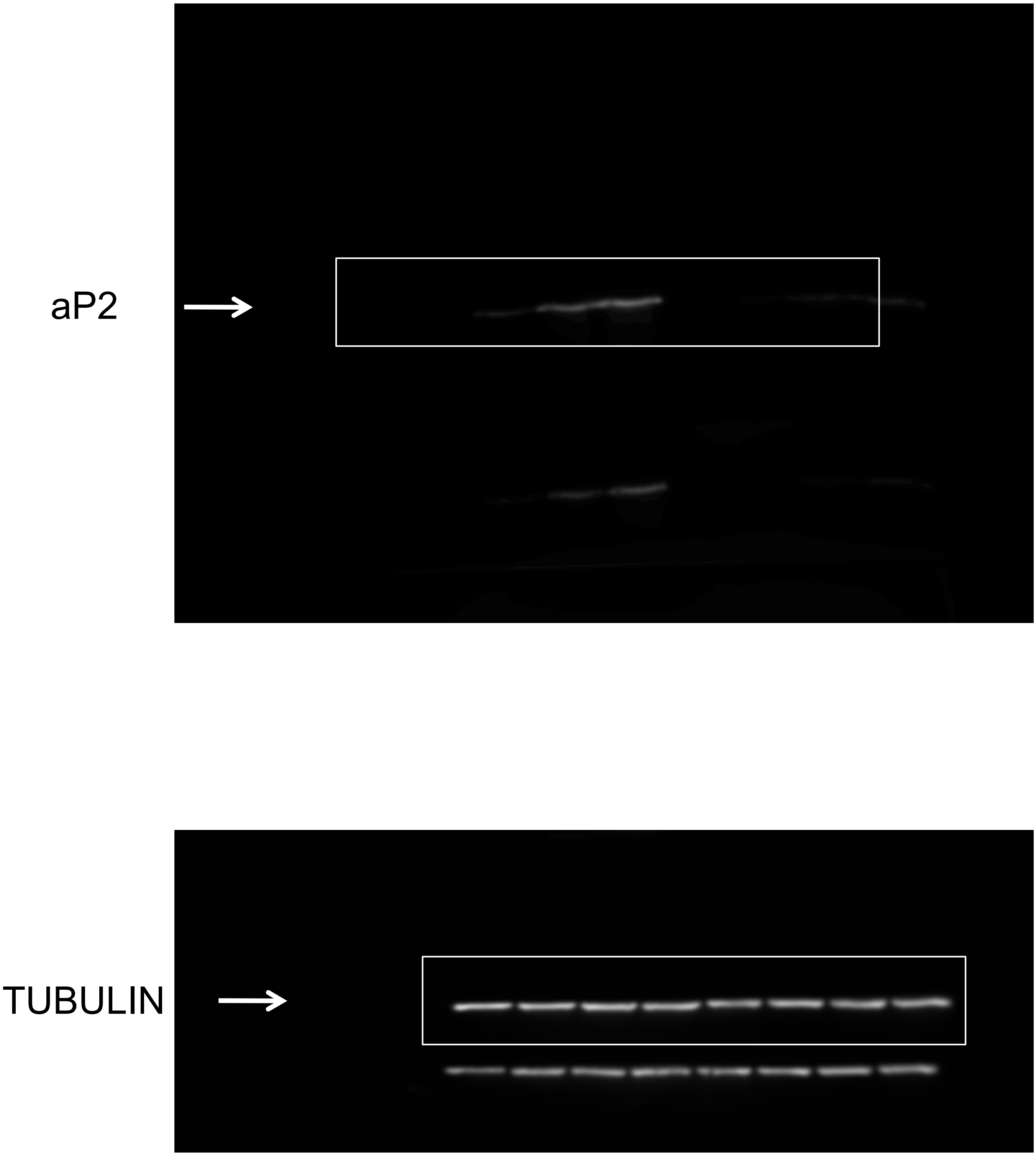

Figure 4c

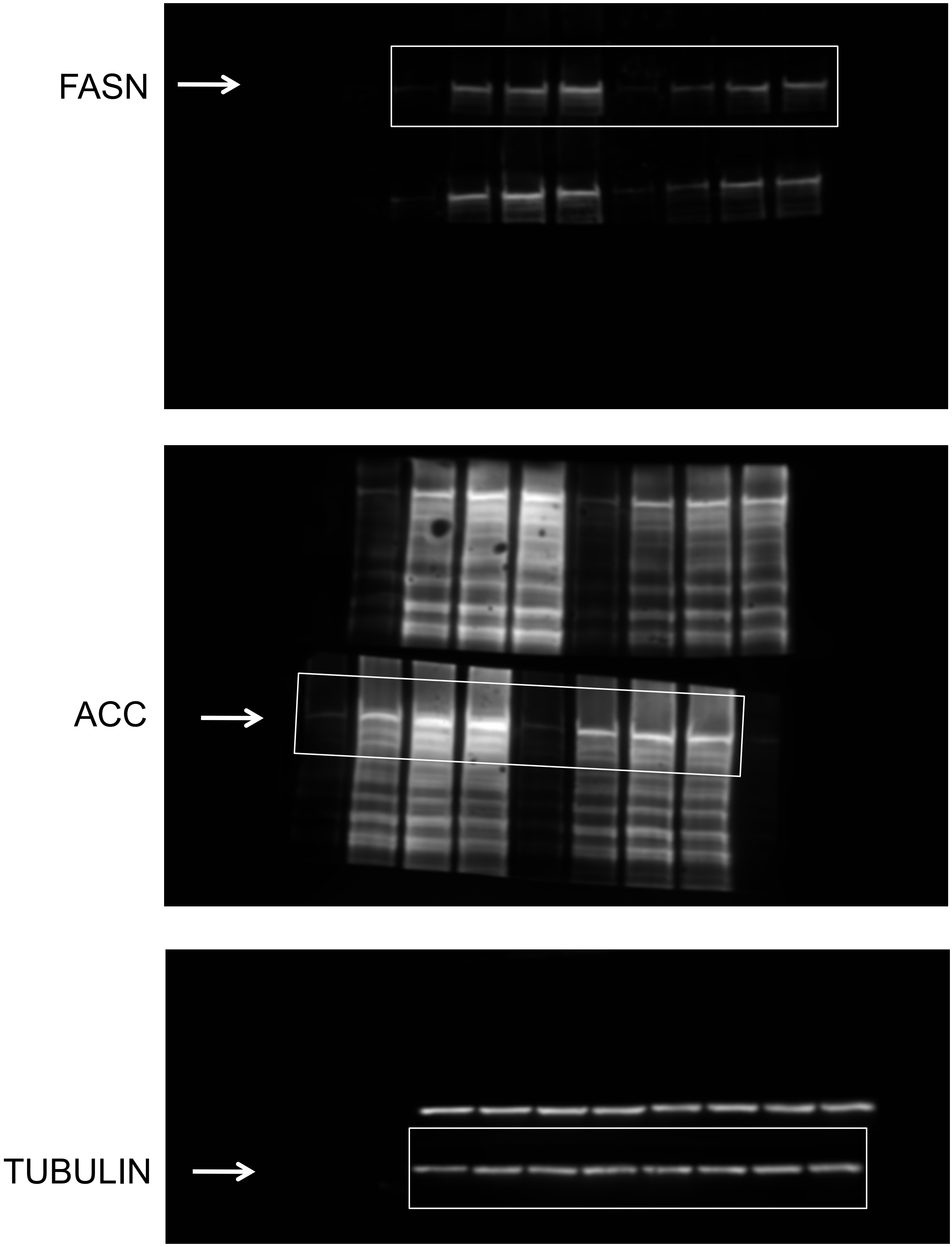

Figure 4e

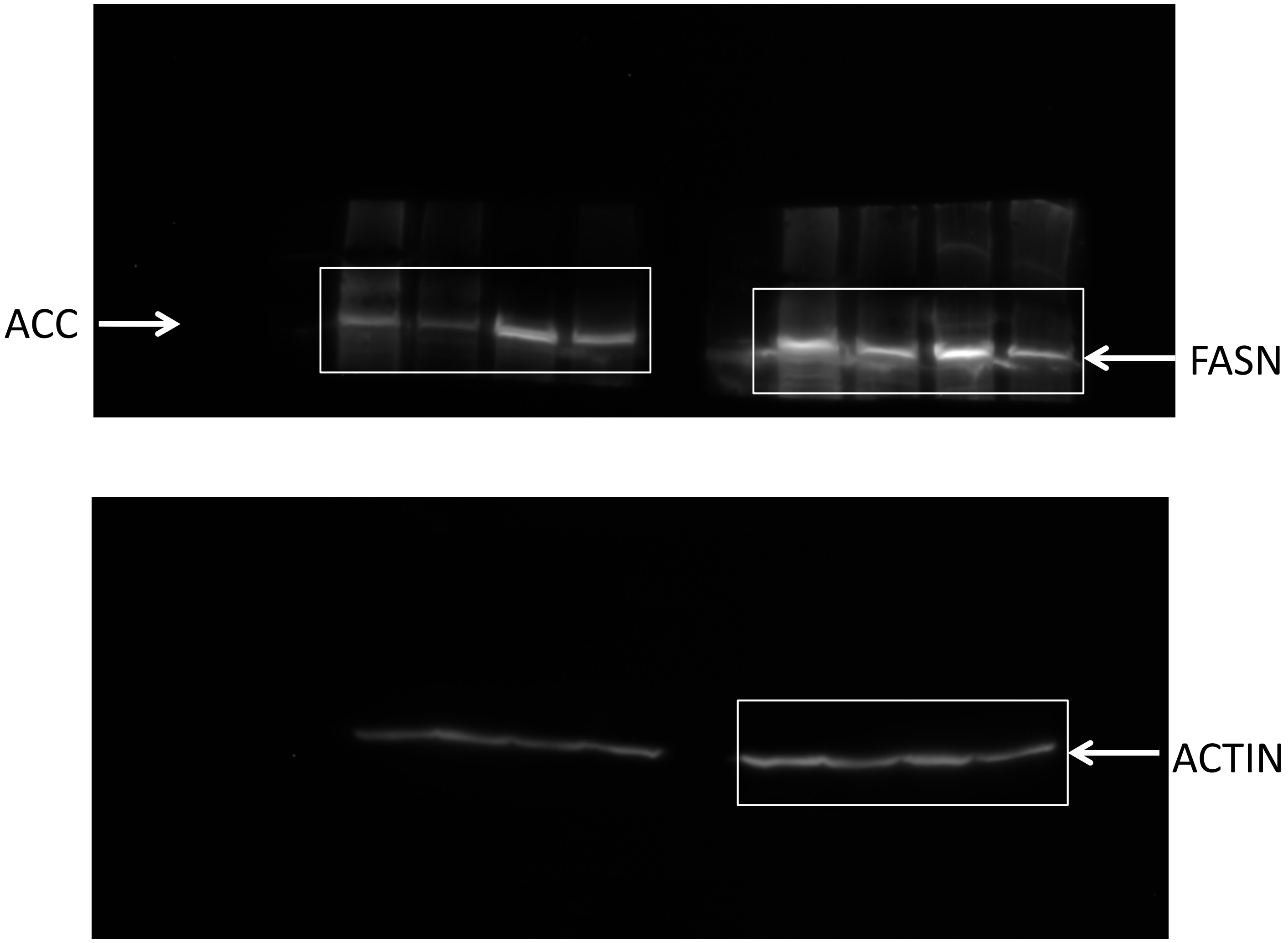

Figure 5a

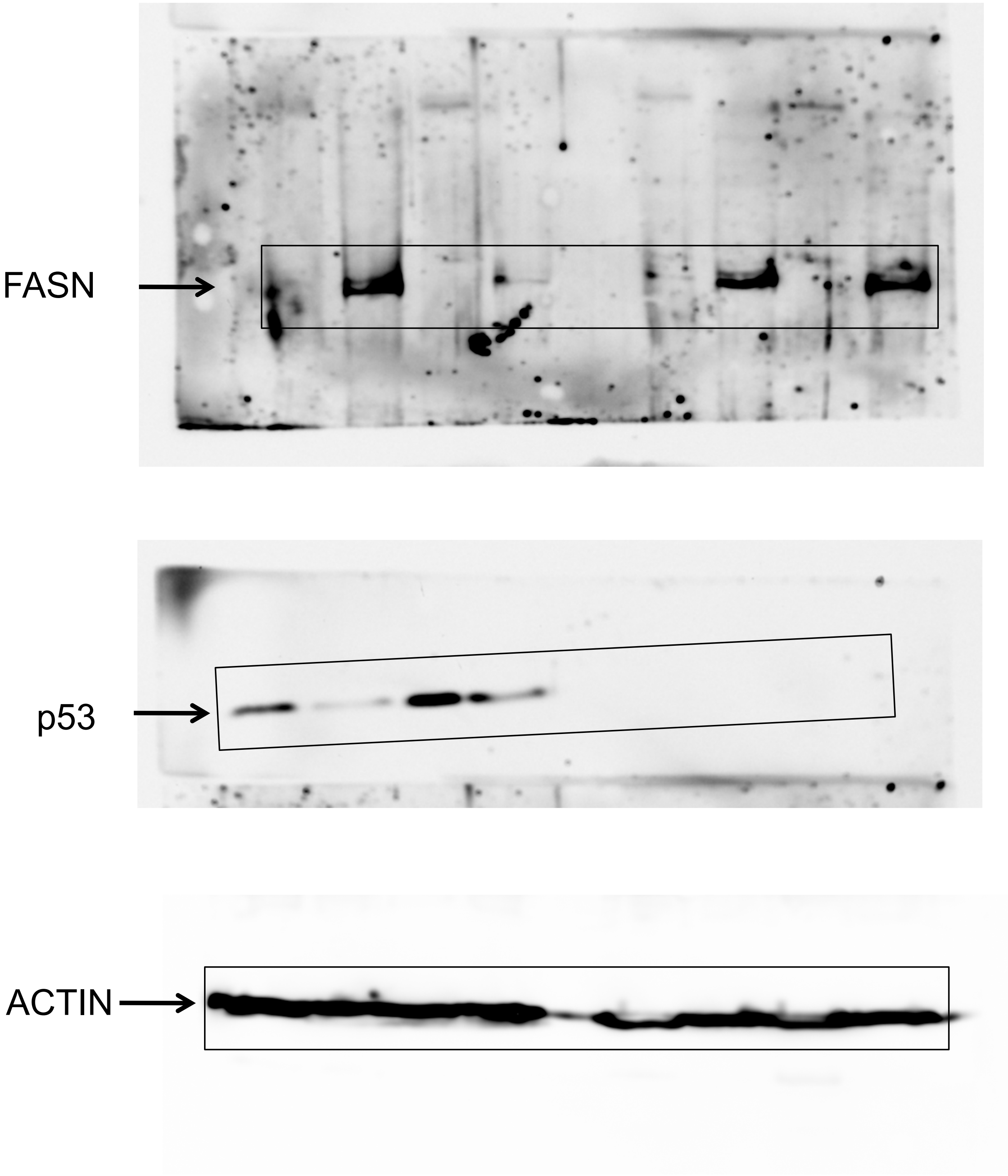

Figure 6a

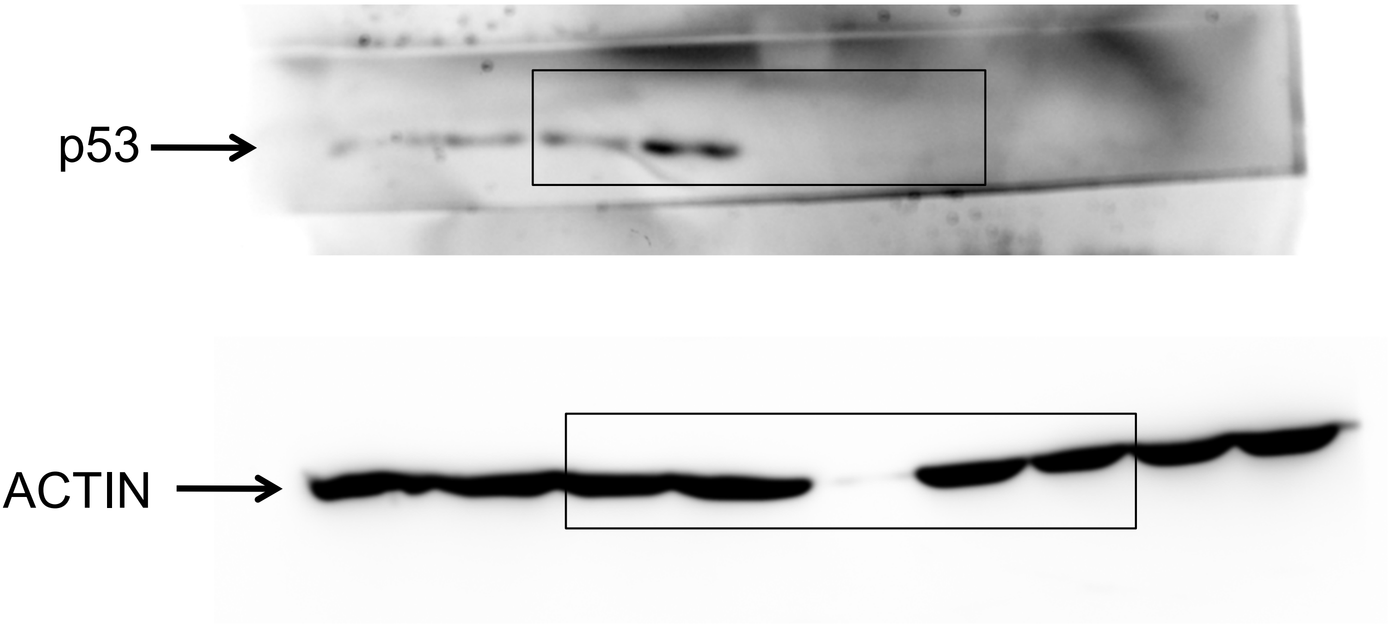

Figure 7b

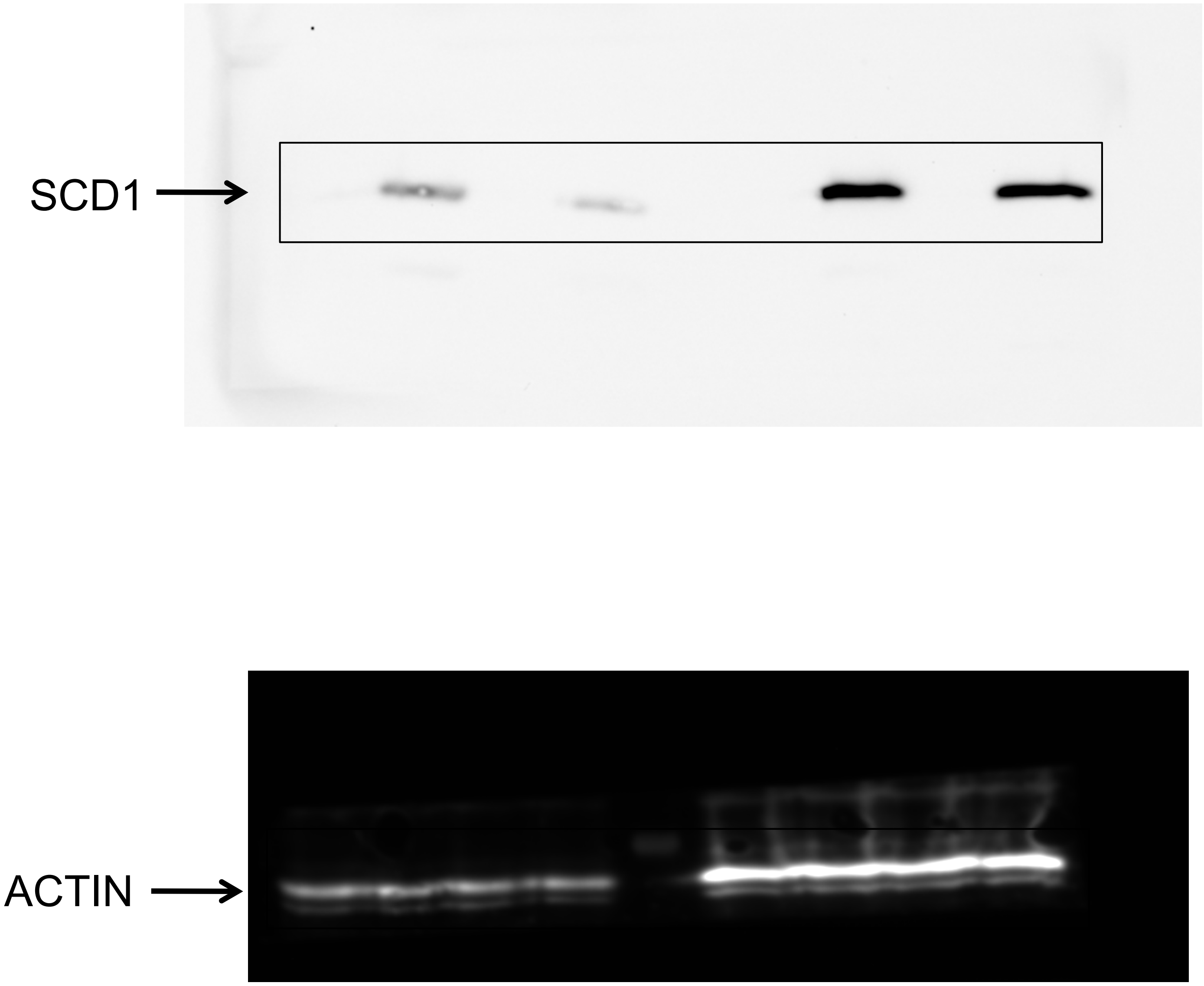

Supplemental Figure 5a

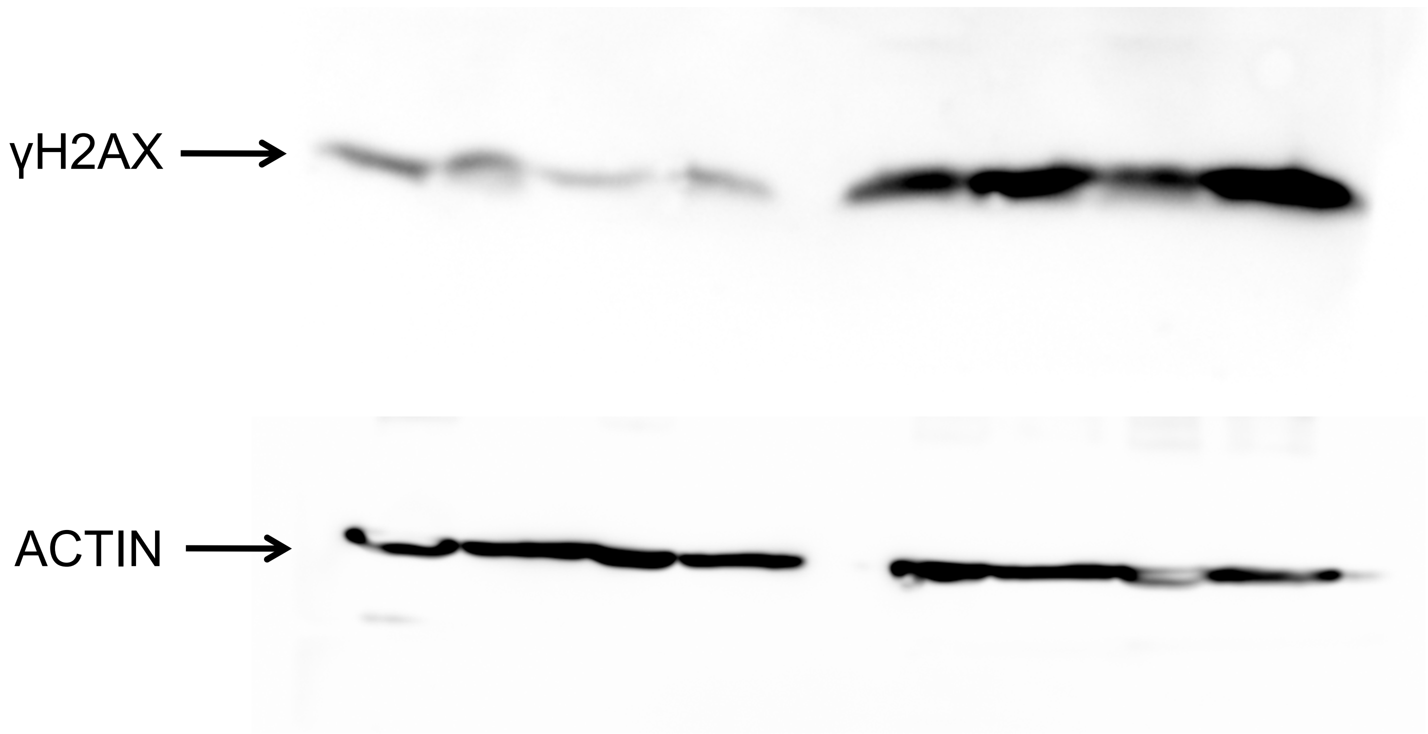

Supplemental Figure 5d

OXYBLOT

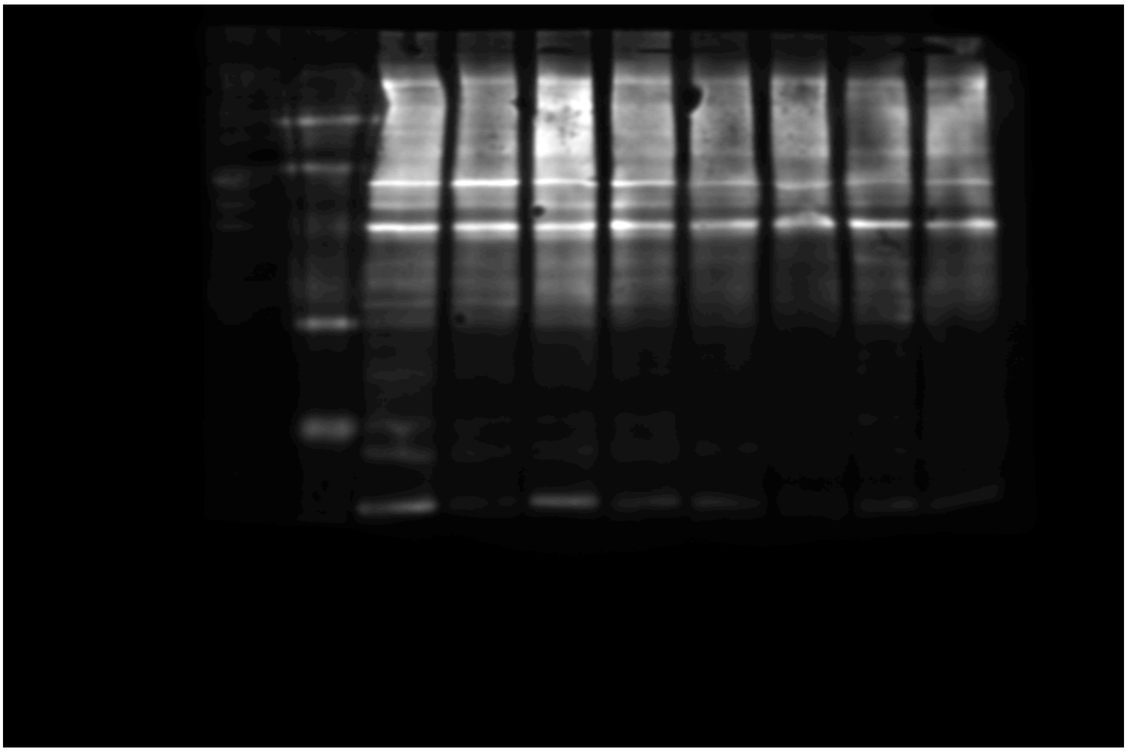

Ponceau

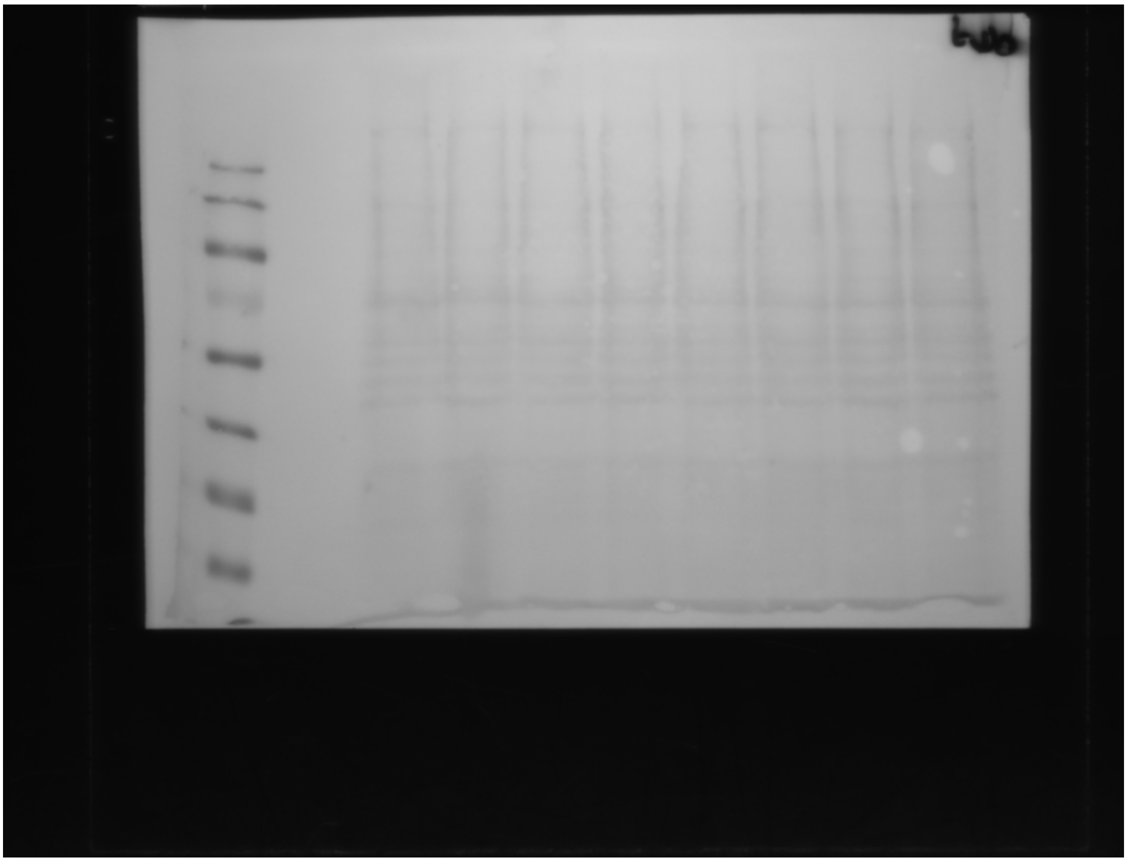

Supplemental Figure 5e

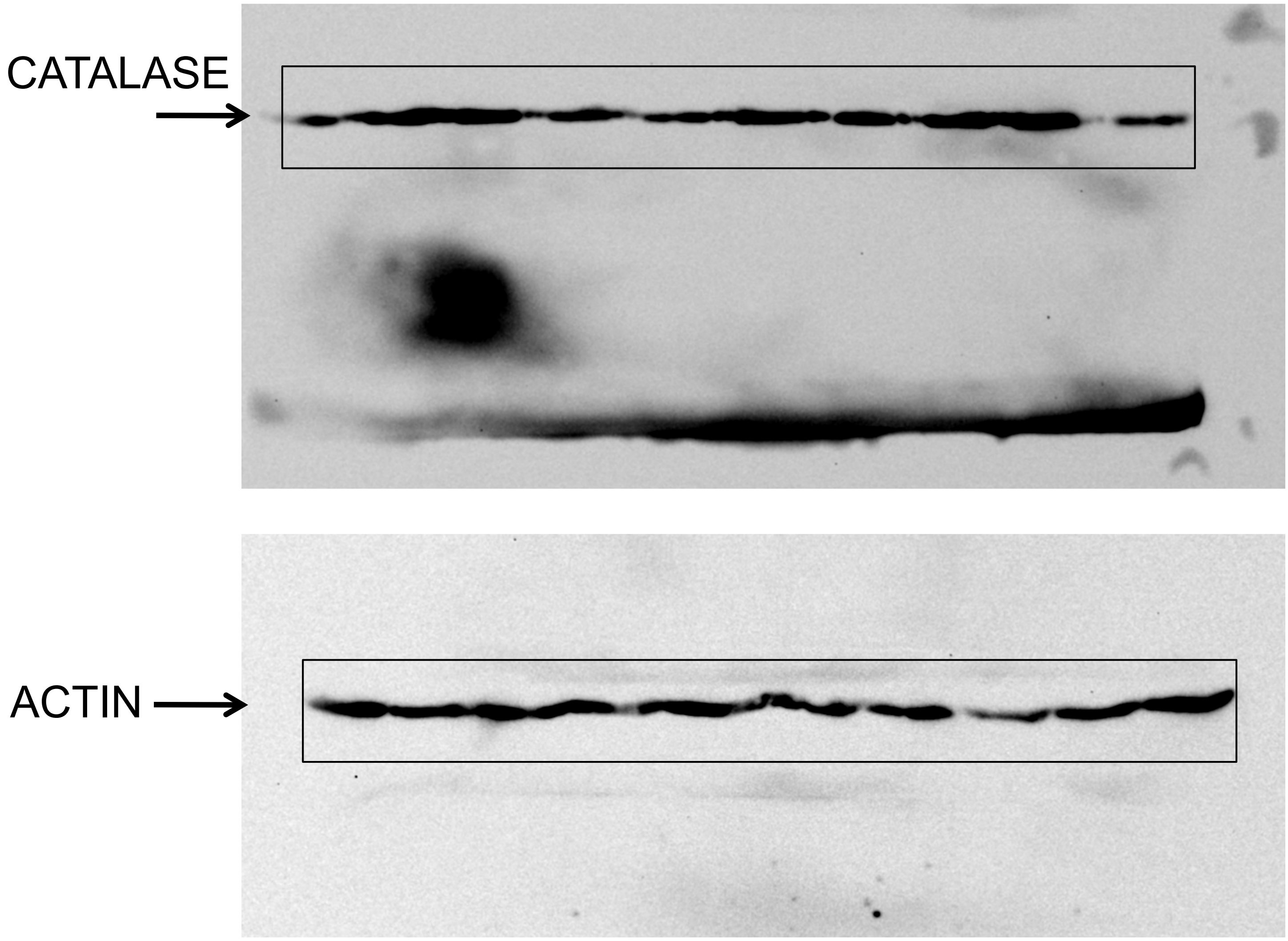

Supplemental Figure 6a

Input E4F1

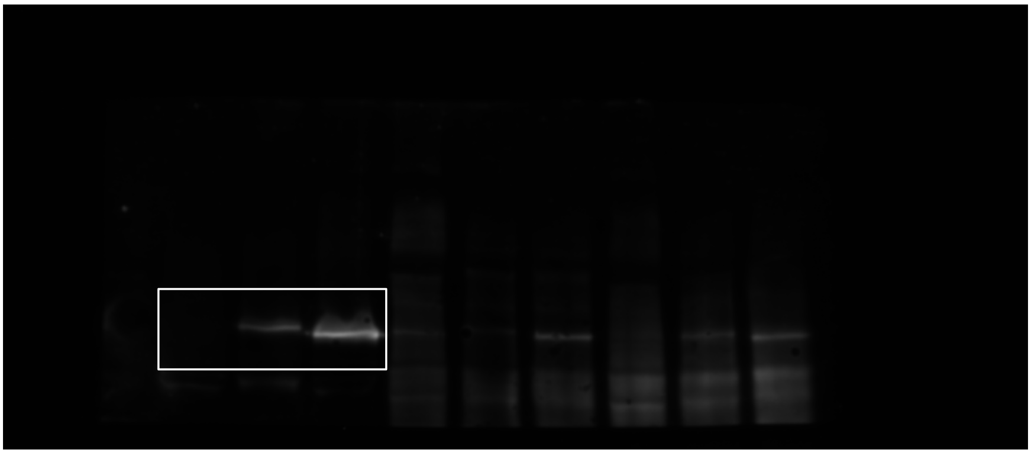

IP Ctl
WB E4F1

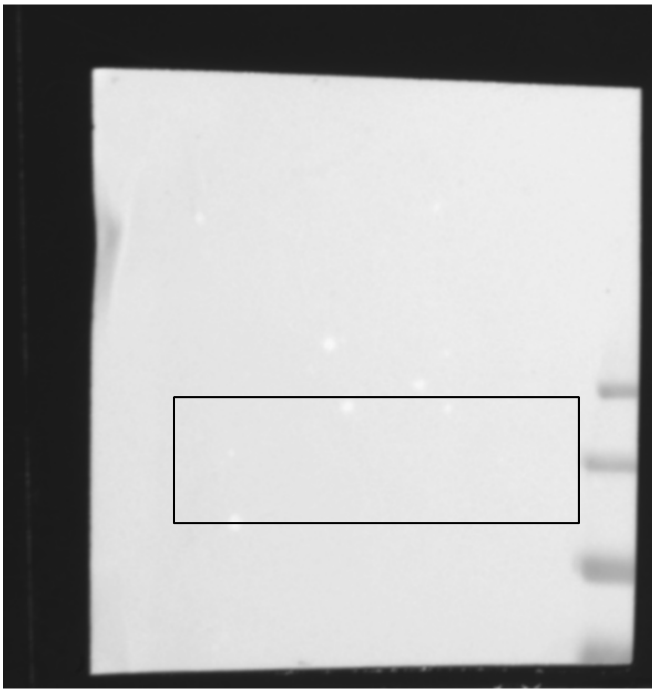

IP p53
WB E4F1

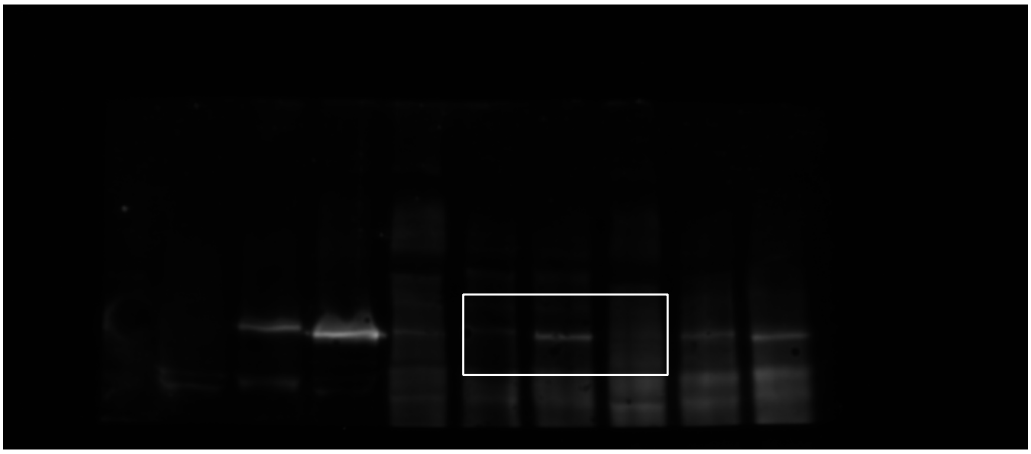

Supplemental Figure 6a

Input p53

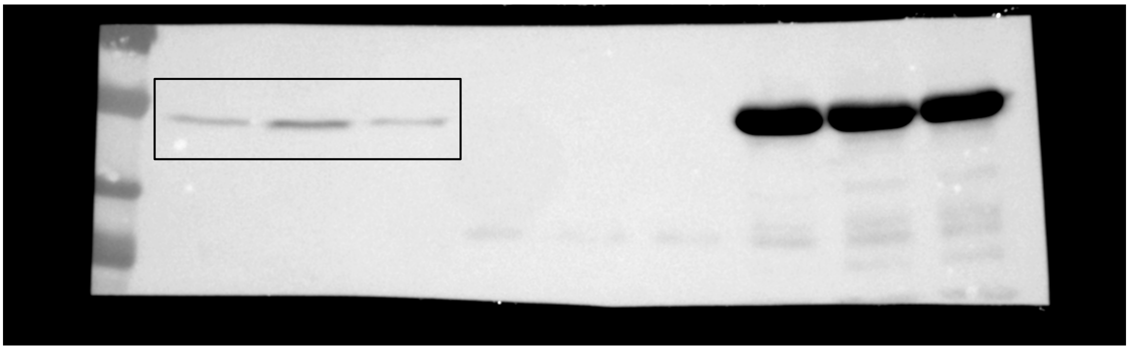

IP Ctl
WB p53

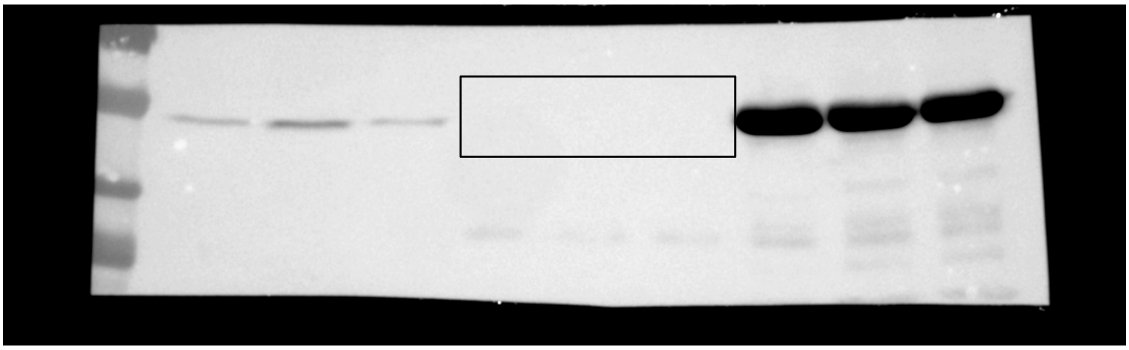

IP p53
WB p53

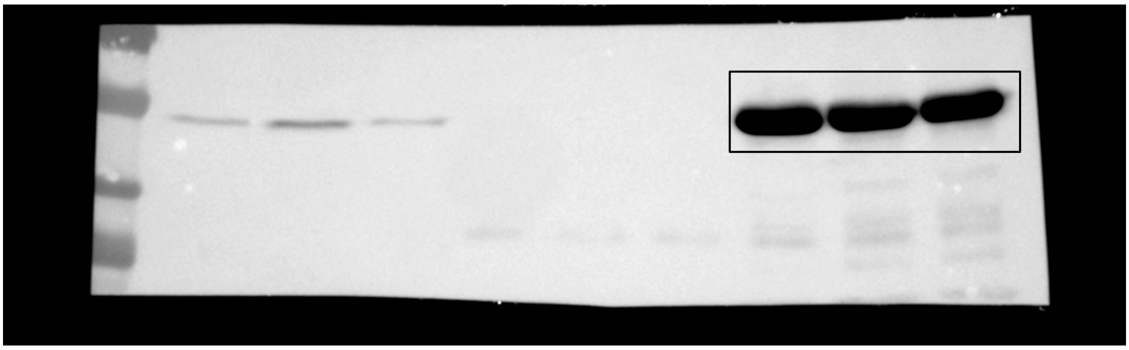

Supplemental Figure 6b

E4F1

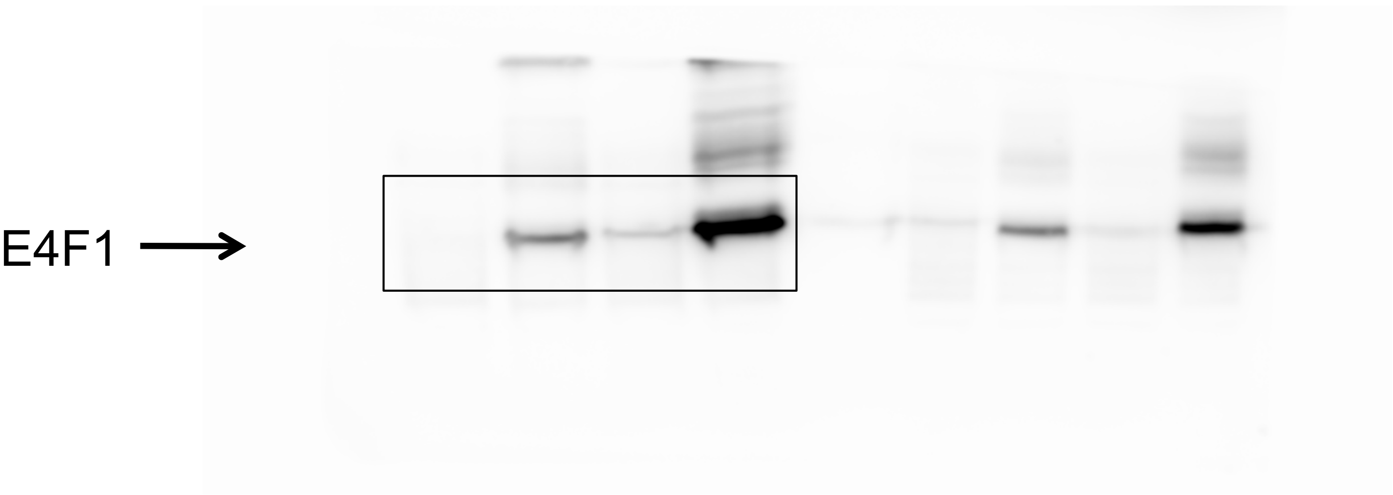

ACTIN

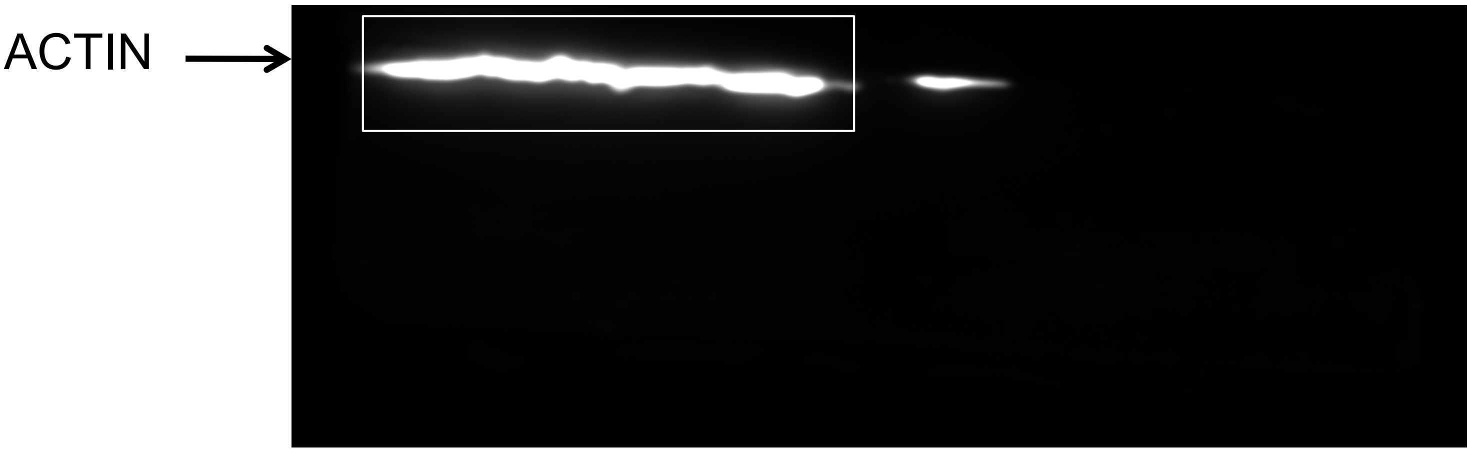

Supplemental Figure 7f

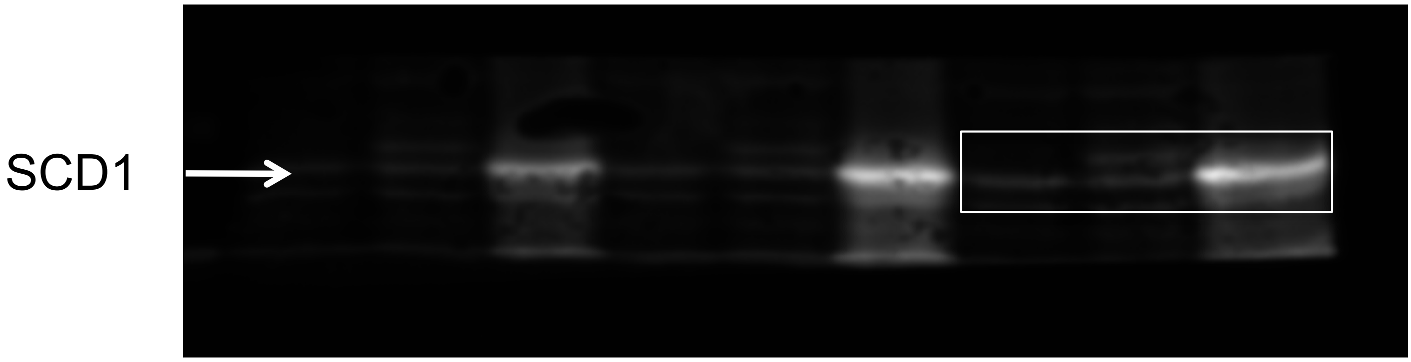

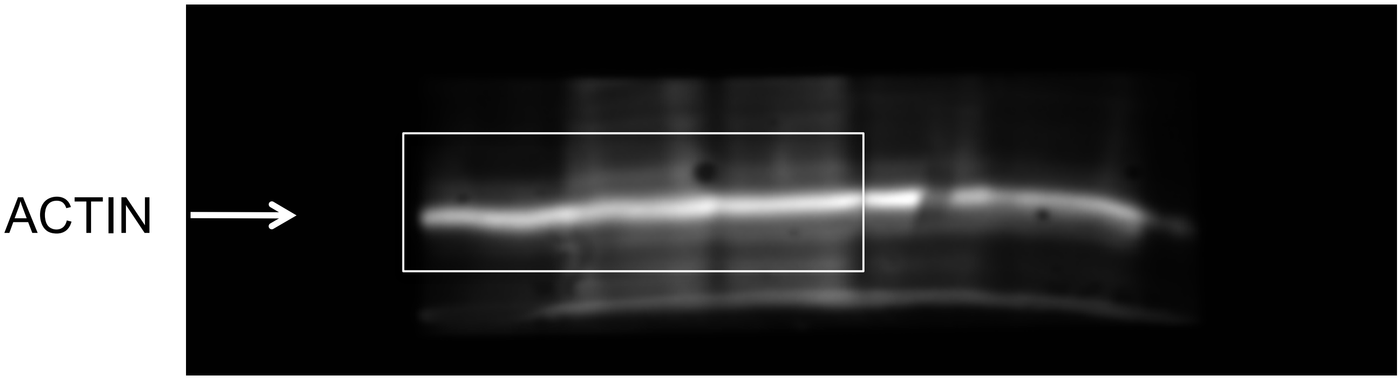

Supplement: Supplementary file 1 — Supplementary Information [file 41467_2021_27307_MOESM1_ESM.pdf]
