# Supplementary material for: The multifunctional protein E4F1 links P53 to lipid metabolism in adipocytes
Source: Nat Commun. 2021 Dec 2;12:7037. doi: 10.1038/s41467-021-27307-3 (PMC8639890; doi:10.1038/s41467-021-27307-3)
Supplement: Supplementary file 2 — Reporting Summary [file 41467_2021_27307_MOESM2_ESM.pdf]

nature portfolio

Corresponding author(s): Dr Laurent Le Cam
Last updated by author(s): 10/01/2021

# Reporting Summary

Nature Portfolio wishes to improve the reproducibility of the work that we publish. This form provides structure for consistency and transparency in reporting. For further information on Nature Portfolio policies, see our Editorial Policies and the Editorial Policy Checklist.

## Statistics

For all statistical analyses, confirm that the following items are present in the figure legend, table legend, main text, or Methods section.

| n/a | Confirmed | |
|---|---|---|
| ☐ | ☒ | The exact sample size ($n$) for each experimental group/condition, given as a discrete number and unit of measurement |
| ☐ | ☒ | A statement on whether measurements were taken from distinct samples or whether the same sample was measured repeatedly |
| ☐ | ☒ | The statistical test(s) used AND whether they are one- or two-sided<br>*Only common tests should be described solely by name; describe more complex techniques in the Methods section.* |
| ☐ | ☒ | A description of all covariates tested |
| ☒ | ☐ | A description of any assumptions or corrections, such as tests of normality and adjustment for multiple comparisons |
| ☐ | ☒ | A full description of the statistical parameters including central tendency (e.g. means) or other basic estimates (e.g. regression coefficient) AND variation (e.g. standard deviation) or associated estimates of uncertainty (e.g. confidence intervals) |
| ☐ | ☒ | For null hypothesis testing, the test statistic (e.g. $F$, $t$, $r$) with confidence intervals, effect sizes, degrees of freedom and $P$ value noted<br>*Give P values as exact values whenever suitable.* |
| ☒ | ☐ | For Bayesian analysis, information on the choice of priors and Markov chain Monte Carlo settings |
| ☒ | ☐ | For hierarchical and complex designs, identification of the appropriate level for tests and full reporting of outcomes |
| ☒ | ☐ | Estimates of effect sizes (e.g. Cohen's $d$, Pearson's $r$), indicating how they were calculated |

*Our web collection on statistics for biologists contains articles on many of the points above.*

## Software and code

Policy information about availability of computer code

| | |
|---|---|
| Data collection | No public datasets were analyzed in this study |
| Data analysis | Adobe Photoshop CS4 was used to crop images from unprocessed images. Adipocyte surface on H/E stained sections of WAT was quantified using ImageJ 1.43u software. Statistical significance was evaluated using two-sided non-parametric Mann-Whitney U tests with the BiostaTGV software from Sorbonne-Paris University (https://biostatg.sentiweb.fr). |

For manuscripts utilizing custom algorithms or software that are central to the research but not yet described in published literature, software must be made available to editors and reviewers. We strongly encourage code deposition in a community repository (e.g. GitHub). See the Nature Portfolio guidelines for submitting code & software for further information.

## Data

Policy information about availability of data

All manuscripts must include a data availability statement. This statement should provide the following information, where applicable:

- Accession codes, unique identifiers, or web links for publicly available datasets
- A description of any restrictions on data availability
- For clinical datasets or third party data, please ensure that the statement adheres to our policy

The source data related (uncroped gels and raw data) to Figures 1-7 and Supplementary Figures 1-8 is provided as a Source Data file. The clinical data from obese patients and lean individuals are provided in table 2. Primers list is provided in supplementary table 1 (in the Supplementary Information file). All data that support the findings of this study are available within the article, its Supplementary Information, or from the corresponding author upon reasonable request. A reporting summary for this article is available as Supplementary Information file.

# Field-specific reporting

Please select the one below that is the best fit for your research. If you are not sure, read the appropriate sections before making your selection.

- [x] Life sciences
- [ ] Behavioural & social sciences
- [ ] Ecological, evolutionary & environmental sciences

For a reference copy of the document with all sections, see nature.com/documents/nr-reporting-summary-flat.pdf

# Life sciences study design

All studies must disclose on these points even when the disclosure is negative.

| | |
|---|---|
| Sample size | For in vivo experiments, the size of the experimental groups analyzed in this study was determined based on previous comparable studies from our team and based on pilot study data (Lacroix et al., PNAS, 2016; Goguet-Rubio et al., PNAS, 2016). All experimental groups included a minimum of 5 animals per experimental group to reach statistical significance with an alpha value of 0,05. In vitro experiments were realized on 3 to 5 independent populations of primary cells of a given genotype as indicated in the figure legends.<br>For in vitro experiements, no statistical method was used to predetermine sample size. |
| Data exclusions | No data was excluded from the analyses. |
| Replication | In vivo studies were performed on a sufficient number of animals per genotype with a minimum of 5 animals per experimental group. These data were reproducible and were represented as mean+/-SEM.<br>The in vitro data were obtained from 3 to 5 independent experiments as indicated in the figure legends. |
| Randomization | Animals (littermates) were randomized in experimental groups after genotyping.<br>Obese patients were included in experimental groups depending on their BMI>47.<br>For in vitro experiments, samples were included in experimental groups dependong on their genotype. |
| Blinding | In vivo experiments were performed blindly by the same researchers without knowing the animal genotype.<br>The same investigator was in charge of genotyping /processing the cells and collecting/analysing data, a procedure that precuded blinding. |

# Reporting for specific materials, systems and methods

We require information from authors about some types of materials, experimental systems and methods used in many studies. Here, indicate whether each material, system or method listed is relevant to your study. If you are not sure if a list item applies to your research, read the appropriate section before selecting a response.

## Materials & experimental systems

| n/a | Involved in the study | |
|---|---|---|
| [ ] | [x] | Antibodies |
| [ ] | [x] | Eukaryotic cell lines |
| [x] | [ ] | Palaeontology and archaeology |
| [ ] | [x] | Animals and other organisms |
| [ ] | [x] | Human research participants |
| [ ] | [x] | Clinical data |
| [x] | [ ] | Dual use research of concern |

## Methods

| n/a | Involved in the study | |
|---|---|---|
| [x] | [ ] | ChIP-seq |
| [x] | [ ] | Flow cytometry |
| [x] | [ ] | MRI-based neuroimaging |

## Antibodies

Antibodies used

anti-p53 antibody (clone 1C12, Cell Signalling, #2524S, lot17, 1/1000 for WB, 1/500 for IHC, 10ul per IP);
homemade anti-E4F1 (Fajas et al., PNAS, 2000, 1/2000 for WB);
anti-SCD1 (clone M38, Cell Signaling, #2438, lot2, 1/1000 for WB, 1/300 for IHC);
anti-FASN (clone H300, Santa Cruz, #sc20140, lotF1110; 1/1000 for WB);
anti-ACC (Cell Signaling, #3662, lot9, 1/1000 for WB);
anti-Catalase (clone H9, Santa Cruz, #271803, lotC1026; 1/1000 for WB);
anti-PPARγ (clone E8, Santa Cruz, #SC7273, lotL3013; 1/500 for WB);
anti-C/EBPα (clone 14AA, Santa Cruz, #sc61, lotA2814, 1/1000 for WB);
anti-aP2 (clone B4, Santa Cruz, #sc271529, lotL3013, 1/1000 for WB);
anti-γH2AX (clone JBW301, Millipore, #05-636, lotNG1904671, 1/1000 for WB);
anti-ACTIN (Sigma, #A2066, lot100M4782, 1/7000 for WB);
anti-H3K4me3 (clone C42D8, Cell Signaling, #9751S, lot10, 10ul per IP);
anti-H3K9me3 (clone 6F12, Cell Signaling, #5327S, lot1,10ul per IP);
anti-H3K27me3 (clone C36B11, Cell Signaling, #9733S, lot8, 10ul per IP);

| | |
|---|---|
| | anti-H3K18ac (clone D8Z5H, Cell Signaling, #13998S, lot1, 10ul per IP);<br>anti-H3K27ac (clone D5E4, Cell Signaling, #8173, lot1, 10ul per IP);<br>anti-Perilipin (ABR, #PA1 1052, lot619111, 1/500 for IHC);<br>anti-Caspase 3 (clone Asp175, Cell Signaling, #9661S, lot45, 1/500 for IHC);<br>anti-F4/80 (BM8, eBioscience, #11-4801-85, lotE026762, 1/1000 for IHC);<br>anti-Mac2 (clone M3/38, eBioscience, #14-5301-82, lot275, 1/1000 for IHC);<br>Alexa488-coupled secondary antibodies (anti-mouse #A21202 lot436938 1/1000 for IHC, anti-rabbit #A21206 lot439378 1/1000 for IHC);<br>anti-rabbit IgG-HRP (Cell Signaling, #7074S, lot28, 1/7000 for WB);<br>anti-mouse IgG-HRP (Cell Signaling, #7076S, lot35, 1/7000 for WB). |
| Validation | We confirmed the specificity of the p53 (1C12) and the E4F1 antibodies for ChIP and immunoblot applications using p53 and E4f1 KO Mefs, respectively.<br>For Cleaved-Caspase 3, F4/80 and Mac-2 antibodies, we used thymus sections as positive control.<br>All other antibodies were validated by the manufacturers:<br>anti-SCD1 (https://www.cellsignal.com/products/primary-antibodies/scd1-m38-antibody/2438);<br>anti-FASN (https://www.scbt.com/p/fatty-acid-synthase-antibody-h-300);<br>anti-ACC (https://www.cellsignal.com/products/primary-antibodies/acetyl-coa-carboxylase-antibody/3662);<br>anti-Catalase (https://www.scbt.com/fr/p/catalase-antibody-h-9);<br>anti-PPARγ (https://www.scbt.com/fr/p/ppargamma-antibody-e-8);<br>anti-C/EBPα (https://www.scbt.com/p/c-ebp-alpha-antibody-14aa);<br>anti-aP2 (https://www.scbt.com/fr/p/a-fabp-antibody-b-4);<br>anti-γH2AX (https://www.merckmillipore.com/FR/fr/product/Anti-phospho-Histone-H2A.X-Ser139-Antibody-clone-JBW301,MM_NF-05-636);<br>anti-ACTIN (https://www.sigmaaldrich.com/FR/fr/product/sigma/a2066);<br>anti-H3K4me3 (https://www.cellsignal.com/products/primary-antibodies/tri-methyl-histone-h3-lys4-c42d8-rabbit-mab/9751);<br>anti-H3K9me3 (https://www.cellsignal.com/products/primary-antibodies/di-tri-methyl-histone-h3-lys9-6f12-mouse-mab/5327);<br>anti-H3K27me3 (https://www.cellsignal.com/products/primary-antibodies/tri-methyl-histone-h3-lys27-c36b11-rabbit-mab/9733);<br>anti-H3K18ac (https://www.cellsignal.com/products/primary-antibodies/acetyl-histone-h3-lys18-d8z5h-rabbit-mab/13998);<br>anti-H3K27ac (https://www.cellsignal.com/products/primary-antibodies/acetyl-histone-h3-lys27-d5e4-xp-rabbit-mab/8173);<br>anti-Perilipin (https://www.thermofisher.com/antibody/product/Perilipin-A-B-Antibody-Polyclonal/PA1-1052); |

## Eukaryotic cell lines

Policy information about cell lines

| | |
|---|---|
| Cell line source(s) | Mouse embryonic fibroblasts (Mefs) were isolated from E13.5 embryos and included in experimental groups according to their genotype.<br>Pre-adipocytes were prepared from 8-week-old mice and included in experimental groups according to their genotype.<br>The 293T cells were used to produce viral particles. |
| Authentication | Cell line authentication is not relevant for Mef primary cells and pre-adipocytes.<br>The 293T cells were not autentified. |
| Mycoplasma contamination | All populations of primary cells and 293T cells were tested negative for Mycoplasma contamination using a commercial kit (Lonza, #LT07-318). |
| Commonly misidentified lines<br>(See ICLAC register) | Not relevant with the present study. |

## Animals and other organisms

Policy information about studies involving animals; ARRIVE guidelines recommended for reporting animal research

| | |
|---|---|
| Laboratory animals | Mus musculus. The following strains were used in this study: E4F1<tm1.1Llca> (MGI:4867860), E4F1<tm1Pisc> (MGI:3050154), Tg(Adipoq-cre/ERT2)1Soff/J (MGI:5568125), Polr2a<tm1(cre/ERT2)Bbd> (MGI:3772332), Tg(Fabp4-cre)1Rev (MGI:2386686), Trp53<tm1Tyj> (MGI:1857263), Lep<Ob> (MGI:1856424). These strains were interbred and maintained on a mix 129Sv/J; C57Bl/6 background. Males and females of 8-to-12 weeks of age were used as described in the manuscript. |
| Wild animals | The study did not involved wild animals. |
| Field-collected samples | No field collected samples were used in the study. |
| Ethics oversight | Studies were conducted in accordance with the ethic committee for animal warefare of the region Languedoc Roussillon (Comité d'Ethique en Expérimentation Animal Languedoc-Roussillon #CEEA-LR-12116 and #17078-20181009101330v2). |

Note that full information on the approval of the study protocol must also be provided in the manuscript.

## Human research participants

Policy information about studies involving human research participants

| | |
|---|---|
| Population characteristics | Age (between 33 to 73 years), gender (men and women), weight (from 59 to 189kg), size (from 154 to 186cm), and Body Mass Index (BMI) of all obese patients and control lean individuals were provided by the clinicians involved in our study (Francisco J. Tinahones and Fernando Cardona) and are described in table 2. |
| Recruitment | All patients who underwent cholecystectomy at the Virgen de la Victoria Hospital (Malaga, Spain) between August and September 2014 were proposed to participate in the study and samples of visceral fat depots were collected from all those who gave informed consent. We outline here no potential self-selection bias that are likely to impact our results. |
| Ethics oversight | The study was approved by the Research Ethics Committee of Malaga (agreement number PI 12/02355). |

Note that full information on the approval of the study protocol must also be provided in the manuscript.

## Clinical data

Policy information about clinical studies

All manuscripts should comply with the ICMJEguidelines for publication of clinical research and a completedCONSORT checklist must be included with all submissions.

| | |
|---|---|
| Clinical trial registration | *Provide the trial registration number from ClinicalTrials.gov or an equivalent agency.* |
| Study protocol | *Note where the full trial protocol can be accessed OR if not available, explain why.* |
| Data collection | *Describe the settings and locales of data collection, noting the time periods of recruitment and data collection.* |
| Outcomes | *Describe how you pre-defined primary and secondary outcome measures and how you assessed these measures.* |
